# *De novo* Assembly, Characterization of Immature Seed Transcriptome and Development of Genic-SSR Markers in Black Gram [*Vigna mungo* (L.) Hepper]

**DOI:** 10.1371/journal.pone.0128748

**Published:** 2015-06-04

**Authors:** J. Souframanien, Kandali Sreenivasulu Reddy

**Affiliations:** Nuclear Agriculture and Biotechnology Division, Bhabha Atomic Research Centre, Mumbai-400085, India; National Institute of Plant Genome Research, INDIA

## Abstract

Black gram [*V*. *mungo* (L.) Hepper] is an important legume crop extensively grown in south and south-east Asia, where it is a major source of dietary protein for its predominantly vegetarian population. However, lack of genomic information and markers has become a limitation for genetic improvement of this crop. Here, we report the transcriptome sequencing of the immature seeds of black gram cv. TU94-2, by Illumina paired end sequencing technology to generate transcriptome sequences for gene discovery and genic-SSR marker development. A total of 17.2 million paired-end reads were generated and 48,291 transcript contigs (TCS) were assembled with an average length of 443 bp. Based on sequence similarity search, 33,766 TCS showed significant similarity to known proteins. Among these, only 29,564 TCS were annotated with gene ontology (GO) functional categories. A total number of 138 unique KEGG (Kyoto Encyclopedia of Genes and Genomes) pathways were identified, of which majority of TCS are grouped into purine metabolism (678) followed by pyrimidine metabolism (263). A total of 48,291 TCS were searched for SSRs and 1,840 SSRs were identified in 1,572 TCS with an average frequency of one SSR per 11.9 kb. The tri-nucleotide repeats were most abundant (35%) followed by di-nucleotide repeats (32%). PCR primer pairs were successfully designed for 933 SSR loci. Sequences analyses indicate that about 64.4% and 35.6% of the SSR motifs were present in the coding sequences (CDS) and untranslated regions (UTRs) respectively. Tri-nucleotide repeats (57.3%) were preferentially present in the CDS. The rate of successful amplification and polymorphism were investigated using selected primers among 18 black gram accessions. Genic-SSR markers developed from the Illumina paired end sequencing of black gram immature seed transcriptome will provide a valuable resource for genetic diversity, evolution, linkage mapping, comparative genomics and marker-assisted selection in black gram.

## Introduction

Black gram [*V*. *mungo* (L.) Hepper] is an important legume crop in Asia, where it is a major source of dietary protein for its predominantly vegetarian population. It is extensively grown in south and south-east Asia. India is the largest producer of black gram, where about 3.26 million hectares are cultivated with a production of 1.74 million tonnes during 2010–11 [1]. Black gram is a self pollinated diploid legume (2n = 2x = 22) with genome size estimated to be 0.59 pg/1C (574 Megabase pair) [2]. The average yield of black gram is low due to its indeterminate growth habit, non-synchronous maturity, narrow genetic base, and losses due to biotic and abiotic stresses. Conventional breeding of black gram has continued entirely without the aid of molecular methods and made limited use of germplasm resources, resulting in a very narrow genetic base in the domesticated species. As a consequence, black gram genetic improvement programs have made relatively little progress in addressing the primary constraints to crop production, which include a range of biotic (e.g. yellow mosaic disease, powdery mildew, pod borer and bruchids) and abiotic (e.g. drought and salinity) stresses. Complete genome sequence information of black gram is not available as on date and the use of genomic resources in this crop largely depend on the sequence information available in the closely related taxa.

A variety of molecular markers like random amplified polymorphic DNA (RAPD), inter-simple sequence repeat (ISSR), amplified fragment length polymorphism (AFLP) and microsatellite or simple sequence repeat (SSR) have been reported for genetic analysis in black gram [3–5]. In the past, SSR markers derived from related *Vigna* species were used to study their transferability to black gram and with the use of such SSR markers, two linkage maps were also developed in this crop [6,7]. However, the use of transferable SSR markers in these maps was limited and only 47 SSR loci were assigned to the 11 linkage groups. Therefore, efforts are required to develop new polymorphic SSR markers in black gram.

SSRs are markers of choice for crop improvement because of their locus specificity, hypervariability, reproducibility and importantly their codominant nature. SSRs are short tandem repeats of 1–6 bases that occur frequently and randomly in the eukaryotic genome [8]. They are widely distributed in non-coding as well in the transcribed regions of the genome, commonly known as genomic SSR and genic or expressed sequence tag (EST)-SSRs, respectively [9–13]. Genic-SSRs are more useful as genetic markers since they represent variation in the expressed portion of the genome. In addition, genic-SSR markers being part of the transcriptome are highly conserved and show a high rate of transferability to related species or genera [14,15]. Moreover, due to their association with coding sequences, genic-SSRs can also lead to the direct gene tagging for QTL mapping of agronomically important traits and increase the efficiency of marker-assisted selection [16].

A transcriptome is the set of all RNA molecules, including mRNA, rRNA, tRNA, and non-coding RNA, produced in one cell or a population of cells. Next Generation Sequencing (NGS) technologies allow the mass sequencing of genomes and transcriptomes, which produces a vast array of genomic information. One of the most important areas of NGS data analysis is *de novo* genome or transcriptome assembly. *De novo* assembly is essential for studying non-model organisms where a reference genome or transcriptome is not available. RNA-seq technology has provided a novel method for both mapping and quantifying transcriptome (RNA-seq). RNA-seq technology has been successfully applied in many crop plants such as rice and soybean [17,18]. The recent paired-end tag sequencing strategy of RNA-seq further improves the DNA sequencing efficiency and expands short-read lengths, providing a better depiction of transcriptome [19].

Transcriptomic information is used in a wide range of biological studies and provides fundamental insight into biological processes and applications, such as the levels of gene expression [20], identification of micro RNAs [21], development of micro RNA based markers [22], SSR and SNP mining [23–26]. Extensive efforts at sequencing of expressed genomic regions from tissues under different conditions and developmental stages have led to a large number of EST sequences being deposited in the public databases for a number of model species as well as economically important plants. Several legume crops have recently been subjected to intensive analyses using NGS, making marker- assisted breeding a reality [27]. Despite black gram being an important crop of Asia, development and use of molecular markers in this crop is still limited due to lack of genomic resources. There are no SSR markers reported for black gram compared with other *Vigna* species such as mungbean and cowpea. Therefore, the present study was carried out with following objectives: (1) development of a large expressed sequence dataset using the Illumina paired-end sequencing technology; (2) to analyze the frequency and distribution of SSRs in transcribed region of black gram; (3) to develop a large set of genic-SSR markers in black gram; (4) to check the transferability of these genic-SSR markers to other *Vigna* species.

## Materials and Methods

### Sample preparation and sequencing

Black gram cultivar used in this study is maintained at Nuclear Agriculture and Biotechnology Division, Bhabha Atomic Research Centre, Trombay, Mumbai, India. It is grown in the experimental field at Trombay, Mumbai (latitude 18:54N, longitude 72:49E). TU94-2 is a released variety of the institute to which the corresponding author belongs and hence permission is not required. Black gram used in this study is not an endangered species. RNA was isolated from 12 immature seeds of 4 different plants of cultivar TU94-2, which were harvested 4 weeks after flowering. Total RNA was extracted from immature seeds using RaFlextotal RNA isolation kit as per the standard protocol in the manual. The RNA quality and quantity were determined using Qubit Fluorometer. The paired-end cDNA sequencing libraries were prepared from the total RNA, as per the protocol of Illumina TruSeq RNA Sample Preparation V2 kit. Library preparation was started with mRNA fragmentation followed by reverse transcription, second-strand synthesis, paired-end adapter ligation, and finally, index PCR amplification of adaptor-ligated library. Library quantification and quality assessment was performed on Agilent Caliper LabChip GX Bioanalyser using DNA High Sensitivity Assay Kit. The libraries were sequenced in a single lane in Illumina MiSeq using paired end sequencing chemistry (Xcelris Genomics Ltd. Ahmedabad).

### De novo transcriptome assembly

The raw data was filtered using Trimmomatic v0.30 [28]. All the reads with adaptor contamination and reads with low quality value i.e. an average QV less than 20 (QV < 20) were filtered. After the quality filtration, high quality data were assembled with CLC Genomics Workbench on default parameters (minimum contig length: 200, automatic word size: yes, perform scaffolding: yes, mismatch cost: 2, insertion cost: 3, deletion cost: 3, length fraction: 0.5, similarity fraction: 0.8). These final assembled transcript contigs were used for further bioinformatics analysis.

### Prediction of coding sequences (CDS) and functional annotation

CDS were predicted from transcript contigs using ORF-predictor. The longest frame out of six frames was selected. Subsequently, the predicted CDS were annotated using BLASTx. The functional annotation was performed using 45,680 predicted CDS by aligning the CDS to non-redundant protein sequence (nr) databaseof NCBI (http://www.ncbi.nlm.nih.gov) using BLASTx against green plant database. Each transcript showing significant similarity (e-value ≤ 1e-5) was assigned the putative function as that of corresponding protein from green plant database. These annotated CDS were mapped on Gene Ontology (GO) database. GO assignments were used to classify the functions of the predicted CDS. GO mapping was carried out using Blast2GO [29] in order to retrieve GO terms with their BLASTx annotation. Mapping of the CDS to their respective pathways were performed through KEGG (Kyoto Encyclopedia of Genes and Genomes) database [30] (http://www.genome.jp/kegg).

### SSR mining and primer design

SSRs were identified from the transcript sequences using MIcroSAtellite identification tool (MISA) [http://pgrc.ipk-gatersleben.de/misa/]. In this study, the SSRs were considered to contain motifs with two to six nucleotides in size and a minimum of 6, 5, 4, 4, 3 contiguous repeat units for di-, tri-, tetra-, penta- and hexa-nucleotide respectively. Mononucleotide repeats were not included in the SSR search criteria. Based on MISA results, primers were designed to SSR motifs using WebSat (http://purl.oclc.org/NET/websat/) online software [31]. For designing PCR primers, optimum primer length was 22 mer (range: 18–27 mer), optimum annealing temperature was 60°C (range: 57–68°C), GC content ranged from 40–80%, and other parameter values as default. Eighteen black gram varieties were selected to validate the polymorphishm of 55 randomly selected genic-SSR markers. All SSRs were analyzed for their frequency of occurrence and relative abundance. Relative abundance was calculated as SSR per kb of sequence. The SSR markers were classified based on the position of SSR motifs in the gene, i.e., whether the SSR was present in the coding sequence (CDS), in the 5′ or 3′ untranslated region (UTR). ORF Finder software (http://www.ncbi.nlm.nih.gov/gorf/gorf.html) was used to identify start and stop codons and the longest one starting with ATG codon was selected as representing the protein encoding transcript.

### Plant material and DNA extraction

Eighteen black gram genotypes (TU94-2, TW, KU96-3, TAU-1, Nayagarh, RBU-38, LBG-17, T-9, TU-40, Pant U-19, IPU-7-3, IPU-94-1, IPU-02-43, EC168200, Pant U-30, PU-31, LBG402, LBG-693) and 9 *Vigna* species (*V*. *mungo* var. *silvestris*, *V*. *radiata*, *V*. *unguiculata*, *V*. *angularis*, *V*. *umbellata*, *V*. *glabrescens*, *V*. *aconitifolia*, *V*. *trilobata* and *V*. *vexillata*) were used in the study. Total genomic DNA was extracted from young seedlings using DNeasy plant DNA extraction kit (Qiagen). The quantity and quality of DNA was checked using Nanodrop ND 1000 spectrophotometer (Thermo Scientific, USA).

### SSR markers analysis

PCR amplifications were performed in a 25 μl reaction volume containing 10 mM Tris—HCl (pH 8.3), 50 mM KCl, 2.5 mM MgCl_2_, 0.08% Nonidet P40, 0.2 mM each of dNTP, 1.5 pmoles of each forward and reverse primers, 75 ng of genomic DNA and 0.5 unit of *Taq* DNA polymerase (Fermentas Life Sciences). Amplifications were performed in an Eppendorf Master Cycler (Eppendorf, Hamburg, Germany). Amplification conditions were an initial denaturation at 94°C for 3 min and 5 cycles at 94°C for 30 s, 55 to 50°C (-1°C each cycle), 72°C for 1 min followed by 35 cycles at 94°C for 30 s, 50°C for 1 min, 72°C for 1 min. Final extension was performed at 72°C for 7 min. To evaluate the allelic variation and cross species transferability analysis, PCR products were resolved on 3% agarose using TBE buffer, stained with ethidium bromide, and photographed in a gel documentation system (Syngene, UK).

### Statistical analysis

Scoring of bands was done as presence (1) or absence (0) for each SSR allele. Allelic variation among black gram genotypes was calculated as the polymorphic information content (PIC). PIC of each SSR marker was calculated by applying the formula of Anderson et al. [32]: PIC = 1−Σ(P_ij_)^2^, where P_ij_ is the frequency of the j^th^ allele for the i^th^ locus. Data analyses were performed using software NTSYS-pc version 2.0 [33]. Jaccard’s similarity coefficients were subjected to the unweighted pair group method with arithmetic average (UPGMA) to generate the dendrogram.

## Results

### Illumina paired end sequencing and *de novo* assembly

**Fig 1:**
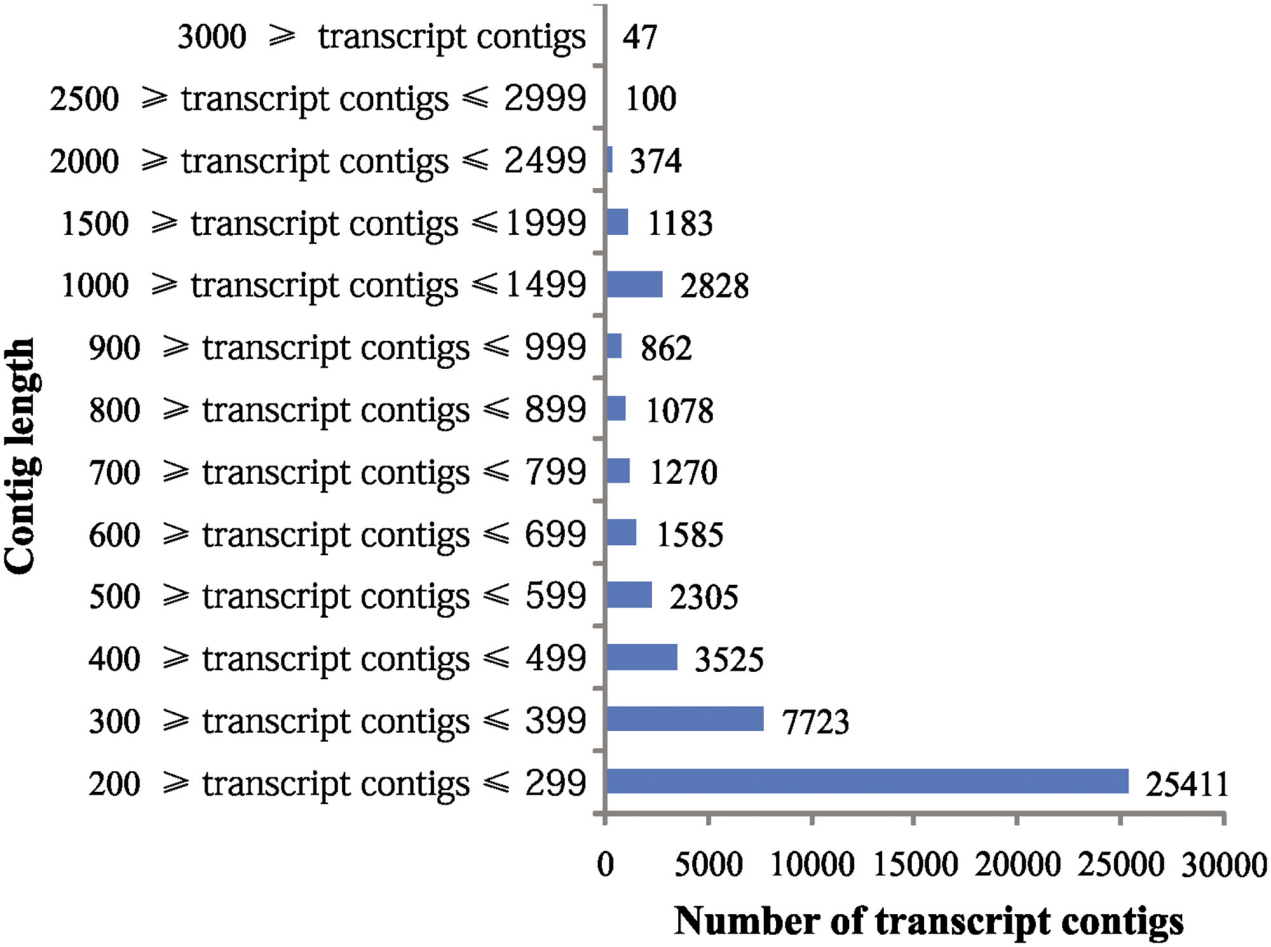
Distribution of transcript contig length assembled from black gram transcriptome sequencing.

Using Illumina paired-end sequencing technology, raw sequencing reads were obtained for black gram seed transcriptome. After stringent quality filtration and data cleaning, approximately 17,254,824 high-quality reads were obtained (base quality more than 20) which resulted in 2, 394,661817 bases (2.39 GB). Illumina reads generated for cv. TU94-2 have been submitted to the NCBI short read archive under the submission ID. SRR 1616991, SRX710526 and study accession SRP 047502. Based on the high quality reads, a total of 48,291 transcript contigs (TCS) were assembled with an average length of 443 bp. The length of contigs ranged from 200 to 6,129 bp (Fig 1). There were 25,411 TCS with length between 200 to 299 bp and 7,723 with length between 300 to 399 bp accounting for 52.6% and 15.9% respectively. TCS with length more than 500 bp accounted for 24.1% (11,632 contigs). There were 4,532 TCS with length more than 1,000 bp accounted for 9.4% of the total TCS.

### Functional annotation

**Fig 2:**
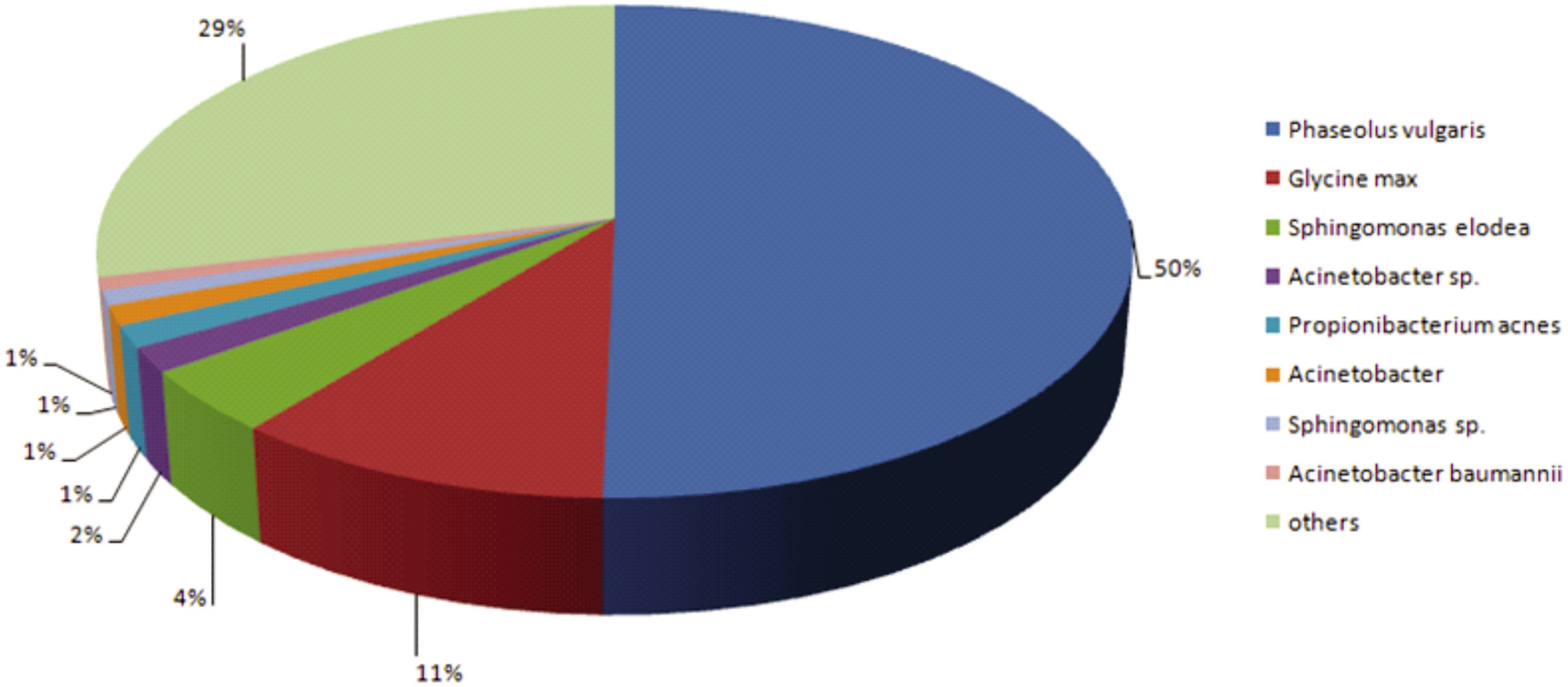
Blast hit distribution of black gram TCS showing similarity with known proteins in non-redundant (nr) database available at NCBI using BLASTx algorithm with an E value threshold of 10^–5^.

Coding sequences (CDS) were predicted from TCS using ORF-predictor. Of the 48,291 TCS, 45,680 (94.5%) were predicted to have CDS. For annotation of TCS, sequence similarity search was conducted against the NCBI non-redundant protein (nr) database using BLASTx algorithm with an *e* value threshold of 10^–5^. The results indicated that out of 45,680 CDS, 33,766 (73.91%) showed significant similarity to known proteins in nr database and 11,914 (26.08%) CDS had no hits in the database. Maximum percentage of CDS showed significant similarity with *Phaseolus vulgaris* (50%) followed by *Glycine max* (11%) (Fig 2).

**Fig 3:**
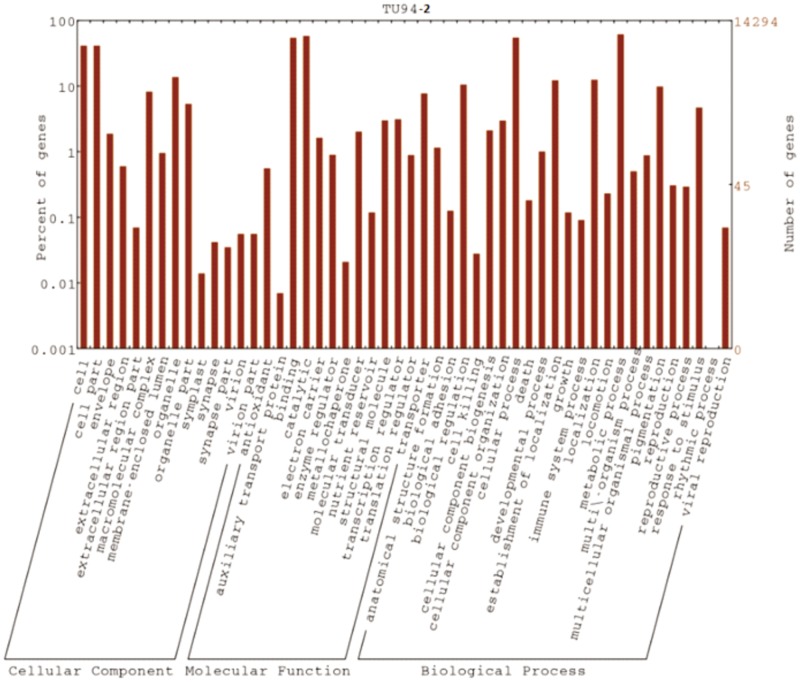
GO Annotation analysis for contigs from *V*. *mungo*.

For functional annotation, the potential coding regions were analyzed by Blast2GO leading to consistent gene annotations, assigning gene names, gene products, EC numbers and Gene Ontology (GO) numbers. Gene Ontology provides a system to categorize description of gene products according to three ontologies: molecular function, biological process and cellular component. In total, 33,766 CDS with BLAST matches to known proteins were assigned to gene ontology classes with 29,564 functional terms (S1 Table). Of them, assignments to the molecular function made up the majority (12,558, 42.5%) followed by biological process (11,039, 37.3%) and cellular component (5,967, 20.2%). Among the assignment made to the molecular function (42.5%), a large proportion of the sequences represented binding and catalytic activities. Under the category of biological process, the majority of the CDS were represented by cellular processes, metabolic processes followed by establishment of localization (Fig 3). For the cellular component category, more CDS were assigned to cell and cell part followed by the organelle. Pathway assignments were carried out according to the Kyoto Encyclopedia of Genes and Genomes (KEGG) pathway database also using BLASTx with an E value threshold of 10^–5^. A total number of 138 unique KEGG pathways were identified, of which the majority of CDS were grouped into purine metabolism (678), pyrimidine metabolism (263), thiamine metabolism (207), starch sucrose metabolism (182) and aminoacyl t-RNA biosynthesis (153).

### Frequency and distribution of SSRs in black gram TCS

**Fig 4:**
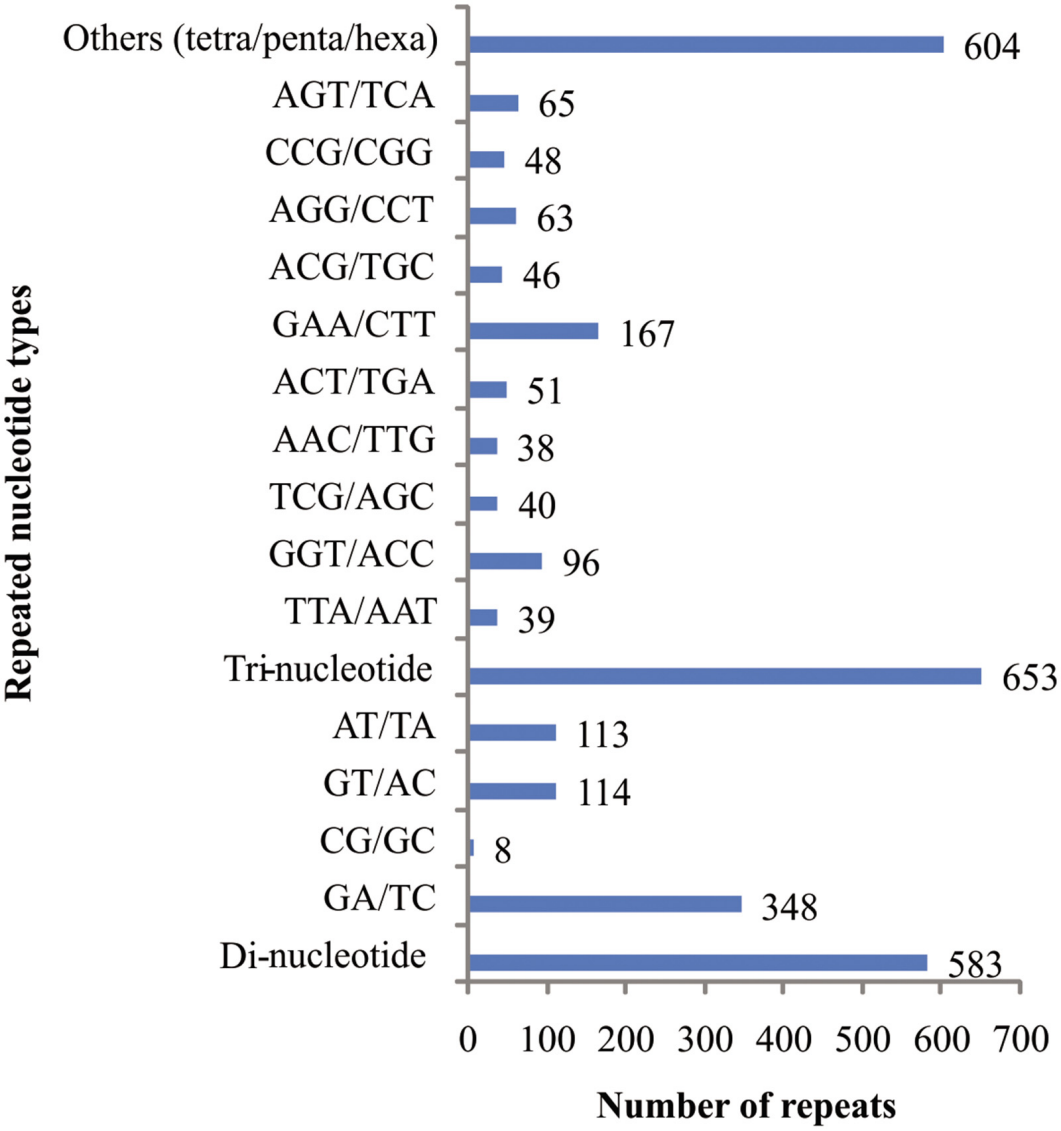
Distribution and frequency of SSR nucleotide classes among different nucleotide types found in the transcriptome sequence.

A total of 48,291 TCS were analysed for SSR motifs, of which, 1,840 SSRs were identified in 1,572 (3.3%) TCS, with an average frequency of one SSR per 11.9 kb. More than one SSR were present in 190 TCS and at least 177 SSRs were present in compound formation. SSR loci with di- and tri-nucleotides constituted 1,236 (67%) of the identified loci. The tri-nucleotide repeats were most abundant (35%) followed by di-nucleotide repeats (32%) among various classes of SSRs (S1 Fig). Hexa-nucleotides were found to be the third largest SSR motif (18%) compared to tetra- (8%) and penta-nucleotides (7%). The number of repeats varied from 6–24 for di-nucleotides, 5–19 for tri-nucleotides, 4–12 for tetra-nucleotides, 4–24 for penta-nucleotides and 3–5 for hexa-nucleotides. Among the various SSRs, GA/TC alone accounted for 59.7% of the total di-nucleotide repeats followed by GT/AC and AT/TA (19.5 and 19.3%). Among the tri-nucleotide repeats, GAA/CTT was found to be the most abundant (25.6%) followed by GGT/ACC (14.7%) compared to other types (Fig 4). Other repeats, AAC/TTG, AAT/ATT, ATC/ATG, AGT/TCA and AGG/CCT constituted 39.2% of the tri-nucleotide repeat.

### Development of genic-SSR markers

**Fig 5:**
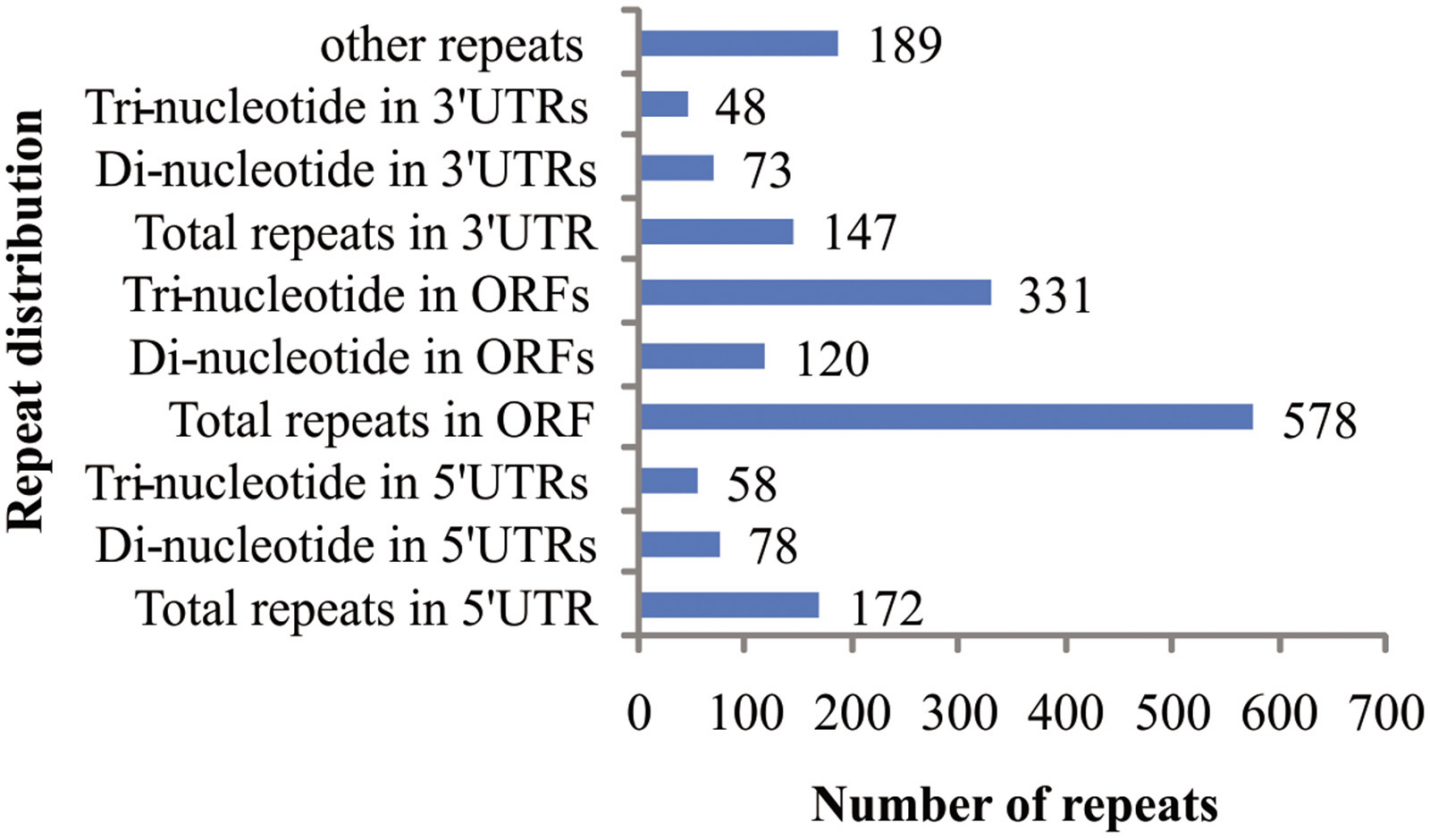
Distribution of genic-SSR motifs in the coding sequence and untranslated region (UTRs) of black gram transcriptome sequences.

Of the 1,840 SSR motif identified, 879 SSRs were found to have flanking region of a minimum of 75 bases. PCR primer pairs were successfully designed for 933 SSR loci. Details about primers sequence and expected product size for 933 genic-SSR markers is provided in S2 Table. Primers could not be designed for the remaining 907 SSR loci because their flanking sequences were either too short or the nature of sequence did not fulfill the criteria for primer design. Sequence analyses revealed that, about 64.4% of the SSR loci were present in the CDS and 35.6% were located in the untranslated regions (UTRs) of the genes (19.2% in 5′ UTR and 16.4% in 3′ UTR). Tri-nucleotide repeats (57.3%) were preferentially present in the CDS, whereas about 47.3% of the di-nucleotide repeats were present in the UTRs (Fig 5).

### Genetic diversity analysis in black gram

**Fig 6:**
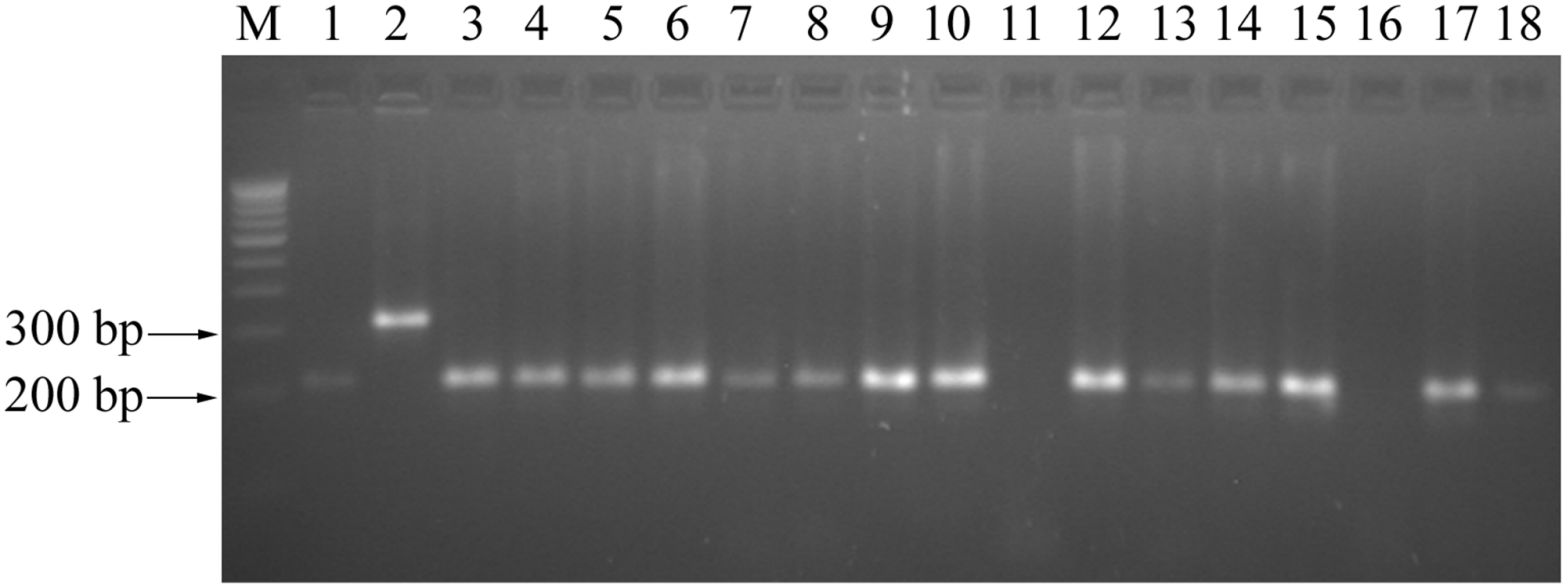
PCR amplification using genic-SSR marker VMgSSR-30 in 18 black gram genotypes.

Fifty five randomly selected genic-SSR markers were used to assess the genetic variability among 18 black gram genotypes. Of these, 32 (58.2%) SSR markers showed polymorphism (Fig 6) and collectively yielded 68 alleles with an average of 2 alleles/ locus. The number of alleles varied from 1 to 3 (S3 Table). Eighteen genic-SSR primers showed null alleles in different black gram genotypes studied. The PIC of individual genic-SSR loci ranged from 0.20 to 0.50, with a mean value of 0.26. Cluster analysis distributed the 18 black gram genotypes in 4 groups (S2 Fig).

### Cross-species transferability of black gram genic-SSR markers

**Fig 7:**
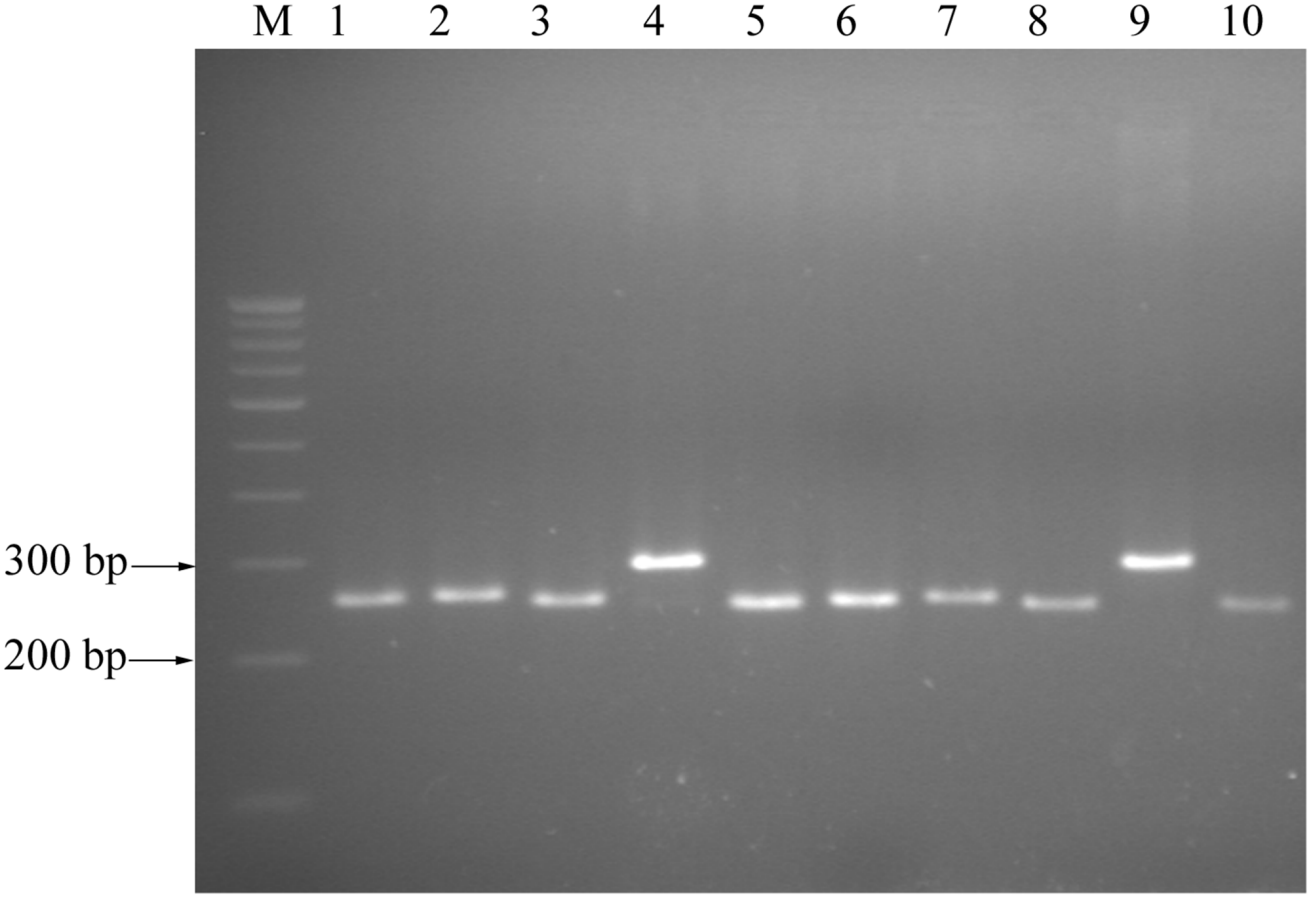
PCR amplification of black gram genic-SSR marker VMgSSR-78 in related *Vigna* species.

Transferability of black gram genic-SSR markers across 9 *Vigna* species was assessed by screening 45 genic-SSR markers. Thirty eight genic-SSR markers (84.4%) were transferable to other *Vigna* species and 7 genic-SSR markers did not produce any amplification. Of those, 13 genic-SSR markers (34.2%) were transferable to more than 7 *Vigna* species, 1 marker (2.6%) was transferable to all 9 *Vigna* species studied (S4 Table). Size variation was observed among different *Vigna* species (Fig 7). Cluster analysis distributed the 9 *Vigna* species into two groups (S3 Fig).

## Discussion

*De novo* transcriptome sequencing allows the study of gene expression in any organism without any prior knowledge of gene sequence. RNA-Seq allows the profiling of the whole population of mRNA in any eukaryotic species and enables mapping and quantification of whole transcripts. Illumina transcriptome *de novo* sequencing and assembly have been successfully used for crop plants such as sweet potato [23], rubber tree [25], *Chrysanthemum* [26], celery [34] and alfalfa [35]. Illumina MiSeq paired end chemistry was used to develop an extensive collection of expressed sequence reads from black gram immature seeds and mined for genic-SSR markers. A total of 17,254,824 high quality sequence reads were assembled to generate 48,291 TCS, which together represented a large fraction of the black gram transcriptome and helped to develop a comprehensive set of genic-SSR markers. Only 73.9% of the TCS showed significant hits in the NCBI non-redundant protein database. Compared with mungbean [24], in which only 41% had BLAST hits in nr database, the higher percentage in this study was partially due to the higher frequency of TCS (11,632 contigs versus 5,254 in mungbean) of long sequences (> 500bp). The longer contigs were more likely to have BLAST matches in the protein databases [36]. Twenty six percent (11,914) of the CDS did not show any hit in the database. Because the significance of sequence similarity depends, in part, on the length of the query sequence, many of the short sequencing reads obtained from next generation sequencing cannot often be matched to known genes [37]. In addition, the genes that were not matched with functional annotations were probably composed of (i) mutants from alternative splicing, novel gene products, or differentially expressed genes [38]; (ii) unique contigs being part of a consensus sequence representing 3'-UTRs; C-termini or 3' sequences which are often less conserved than other transcript regions [39]. Black gram CDS showed more similarity (50%) with common bean. This could be due to the homology between the two genomes as both belong to subtribe Phaseolinae. Previously, genome conservation among different legume genera was detected with DNA markers [6,7,14]. High degree conservation and collinearity between mungbean and common bean was revealed through comparative mapping [40]. In the present study, pathway assignment using KEGG database grouped majority of the CDS (678) under purine metabolism. Synthesis of the purine ring is a central metabolic function of all cells. It is employed in specialized tissues to assimilate and detoxify NH_3_. In tropical legumes such as soybean and cowpea, purine pathway has been found to plays a dominant role in primary nitrogen metabolism [41]. It was shown that this pathway play a major role in nucleotide biosynthesis and degradation in cotyledons and embryonic axes of black gram [42].

### Frequency and distribution of genic-SSRs

Transcriptome analysis provided a powerful tool for the discovery of SSR markers in plants. Large scale genomic and EST sequencing provides the opportunity to evaluate the abundance and relative distribution of microsatellite between transcribed and non-transcribed regions [9]. Using Illumina MiSeq, a large number of transcriptome sequences for black gram have been generated. These sequences provide scope for identification of novel genes and resource for marker development. A total of 48,291 TCS were used for SSR searching and 1,572 (3.3%) TCS contained SSR motifs, generating 1,840 SSRs. Abundance of SSRs in black gram TCS is lower compared to other related *Vigna* species like mungbean (14.6%) [43]. The frequency of microsatellites was significantly higher in ESTs compared to genomic DNA across all species [44]. In this study, the frequency of occurrence for genic-SSRs was one SSR per 11.9 kb compared to 3.3 kb in mungbean [43], 8.4 kb in pigeonpea [45] and 7.4 kb in soybean [46]. However, a direct comparison of abundance estimation and frequency of SSR in different crops is difficult as it is dependent on the SSR search criteria, size of the dataset analysed, database-mining tools and sequence redundancy. Moreover, frequency of SSRs varies broadly depending on the plant species [9,44]. The SSRs were not uniformly distributed in the black gram TCS. Tri-nucleotide repeats were the most abundant (35%), followed by di- (32%) nucleotide repeats. The relative abundance of tri-nucleotide repeats in ESTs sequences has been observed in many other legumes including mungbean [43], cowpea [47], chickpea [48], peanut [49], common bean [50] and *Medicago* [51]. Among di-nucleotide repeats, GA/TC motif was the most abundant repeat (59.7%), followed by GT/AC and AT/TA. The GA/TC motif has been observed as predominant di-nucleotide repeats in the ESTs of many other plants including legumes [47,48,52]. Similarly, GAA/CTT motif (25.6%) was the most predominant among tri-nucleotide repeats and was in close agreement with the observation in cultivated peanut [49] and sweet potato [53]. Among the tri-nucleotide repeat, most common codon repeats (CTC, CTG, CTT, TTA, TTG and CTA) observed were those of leucine- (11.8%) followed by serine [9.9% (AGC, AGT, TCA, TCC,TCT, and TCG)] and arginine [8.1% (AGA, AGG, CGA, CGC, CGG and CGT)]. Similar observations have been made in other studies where most common codon repeats reported were those of serine and leucine in mungbean [43]. Sequence analysis revealed that most of the di-nucleotide repeats (47.3%) were preferentially associated with UTRs, whereas in case of tri-nucleotides, 64.4% of repeats were present within CDS and only 35.6% were present in UTRs. Similar observations have been made in other studies [44,47]. The over representation of tri-nucleotide repeats in the CDS compared with other repeats is expected as need of coding region is to maintain the reading frame. Moreover, it is attributed to absence of frameshift mutations in coding regions when there is a length variation in these SSRs [54].

In the present study, 933 SSR primers were successfully designed from the transcriptome sequence.Transcriptome sequence has been used to develop genic-SSR markers in many legume species including mungbean [43,55], cowpea [47], chickpea [48,56], pigeonpea [45], common bean [50], *Medicago* [51] and alfalfa [35]. As a preliminary study, 55 genic-SSR markers were used to study the genetic diversity in 18 black gram genotypes. The number of alleles ranged from one to three with an average PIC score of 0.26. The polymorphism level in this study was comparable to EST-SSR based studies in other crops including mungbean [43], common bean [50] and sweet potato [53]. In the present study, null alleles were observed for eighteen primers among different black gram genotypes studied. The possible concern with SSRs in general is the possibility of null alleles, which fail to amplify due to primer site variation, and thus do not produce a visible amplicon. Other concern with EST-SSRs is that unrecognized intron splice sites could disrupt priming sites, resulting in failed amplification. Alternatively, large introns could fall between the primers, resulting in a product that is either too large or in extreme cases, failed amplification [57]. UPGMA analysis clearly distributed the black gram genotypes into separate clusters, and most of the genotypes were grouped based on their pedigree. Similar grouping of genotypes based on their pedigree has been reported in black gram using random markers [3]. This indicated that genic-SSR markers developed in this study are highly informative and can be used for genetic diversity and genetic mapping studies.

About 84.4% SSR markers were found transferable to other important *Vigna* species like mungbean, cowpea, azuki bean and rice bean. Most of these *Vigna* species have very limited genomic resources and this is the major constraint in their improvement. Transferability rate of genic-SSR markers to other *Vigna* species appeared to be more or less similar to previous studies. Chaitieng et al. [6] reported that amplification of azuki bean (*V*. *angularis*) microsatellite markers in *V*. *mungo*, *V*. *radiata*, *V*. *aconitifolia* and *V*. *umbellata* was between 68.8 to 90.2%. Whereas, Somta et al. [14] reported that amplification of genic microsatellite markers in 19 taxa of *Vigna* species was between 80% (*V*. *aconitifolia*) to 95.3% (*V*. *reflex-pilosa*). The high amplification rates of black gram genic-SSR markers in *Vigna* species indicate high genome homology among species in this genus and are useful for genome mapping and comparative genomics. Dendrogram generated based on black gram genic-SSR markers clearly separated the nine *Vigna* species into two subgroups. In the present study, *V*. *mungo* and *V*. *mungo* var. *silvestris* showed more similarity and were grouped together. Similar observation has been reported in *Vigna* speciation studies based on transcriptome analysis [58]. Species belonging to subgenus Ceratotropis (*V*. *mungo*, *V*. *radiata*, *V*. *mungo* var. *silvestris*, *V*. *angularis*, *V*. *glabrescens*, *V*. *sublobataand V*. *trilobata*) were placed together with other species belonging to subgenus *Vigna* (*V*. *unguiculataand V*. *vexillata*), which was in complete agreement with taxonomic classification [59]. However, more primers need to be screened to get a clear grouping. These results indicate that genic-SSR markers developed in the study are also efficient to reveal the phylogenetic relationship.

## Conclusion

We have designed 933 genic-SSR markers from the Illumina paired end sequencing of black gram immature seeds. Selected primers were demonstrated for their amplification in black gram and cross species transferability. These developed genic-SSR markers will provide a valuable resource for genetic diversity, evolution, linkage mapping, comparative genomics, gene-based association studies, and marker-assisted selection in black gram. Since these markers were developed based on conserved expressed sequences, they may be valuable for functional analysis of candidate genes. In addition, because of their high transferability, these SSR markers will also provide a platform to expedite the molecular breeding effort in other *Vigna* species. To the best of our knowledge, this is the first attempt to develop transcriptome sequence and develop large numbers of genic-SSR markers in black gram.

## Supporting Information

S1 Fig Distribution of total number of SSRs identified in *Vigna mungo* transcriptome. (TIF)

S2 Fig Dendrogram showing the genetic relationship among 18 black gram genotypes. (TIF)

S3 Fig Dendrogram showing the genetic relationship among 10 *Vigna* species. (TIF)

S1 Table Gene Ontology (GO) classification of the predicted CDS. (XLS)

S2 Table Genic-SSR primers designed from transcriptome sequence of black gram. (DOCX)

S3 Table Number of alleles per locus and polymorphic information content (PIC) value of genic-SSR markers used for studying allelic variation among black gram genotypes. (DOCX)

S4 Table Transferability of black gram genic-SSR markers to other *Vigna* species. (DOCX)
