# Supplementary figures and images for: De novo Assembly, Characterization of Immature Seed Transcriptome and Development of Genic-SSR Markers in Black Gram [Vigna mungo (L.) Hepper]
Source: PLoS One. 2015 Jun 4;10(6):e0128748. doi: 10.1371/journal.pone.0128748 (PMC4456365; doi:10.1371/journal.pone.0128748)

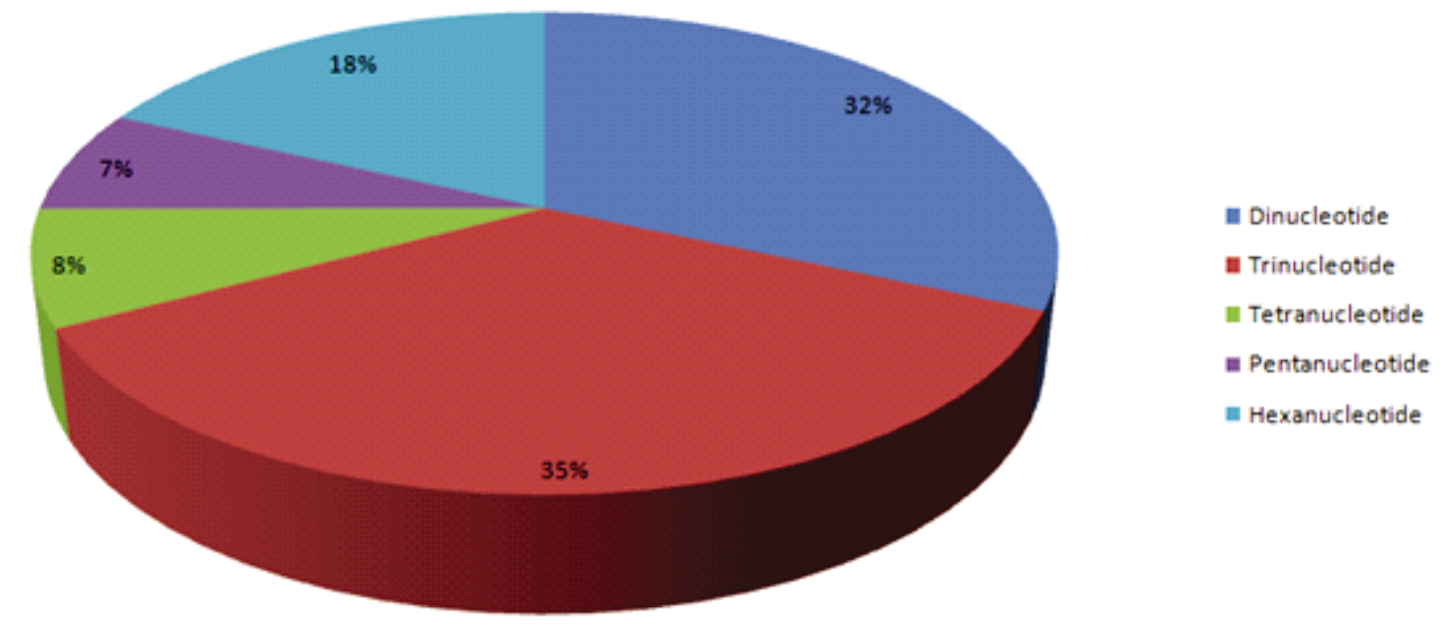

Supplement: S1 Fig — (TIF) [file pone.0128748.s001.tif]

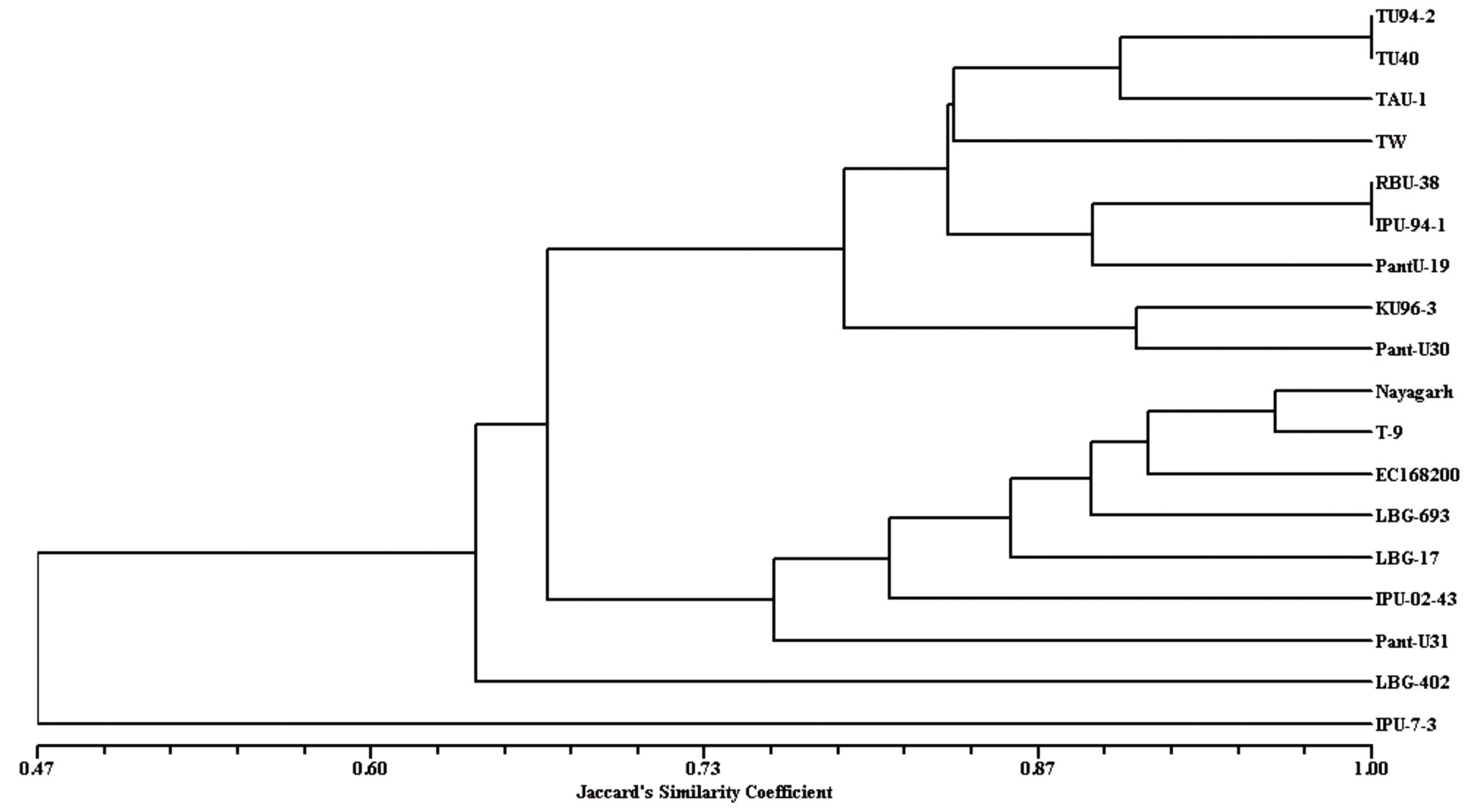

Supplement: S2 Fig — (TIF) [file pone.0128748.s002.tif]

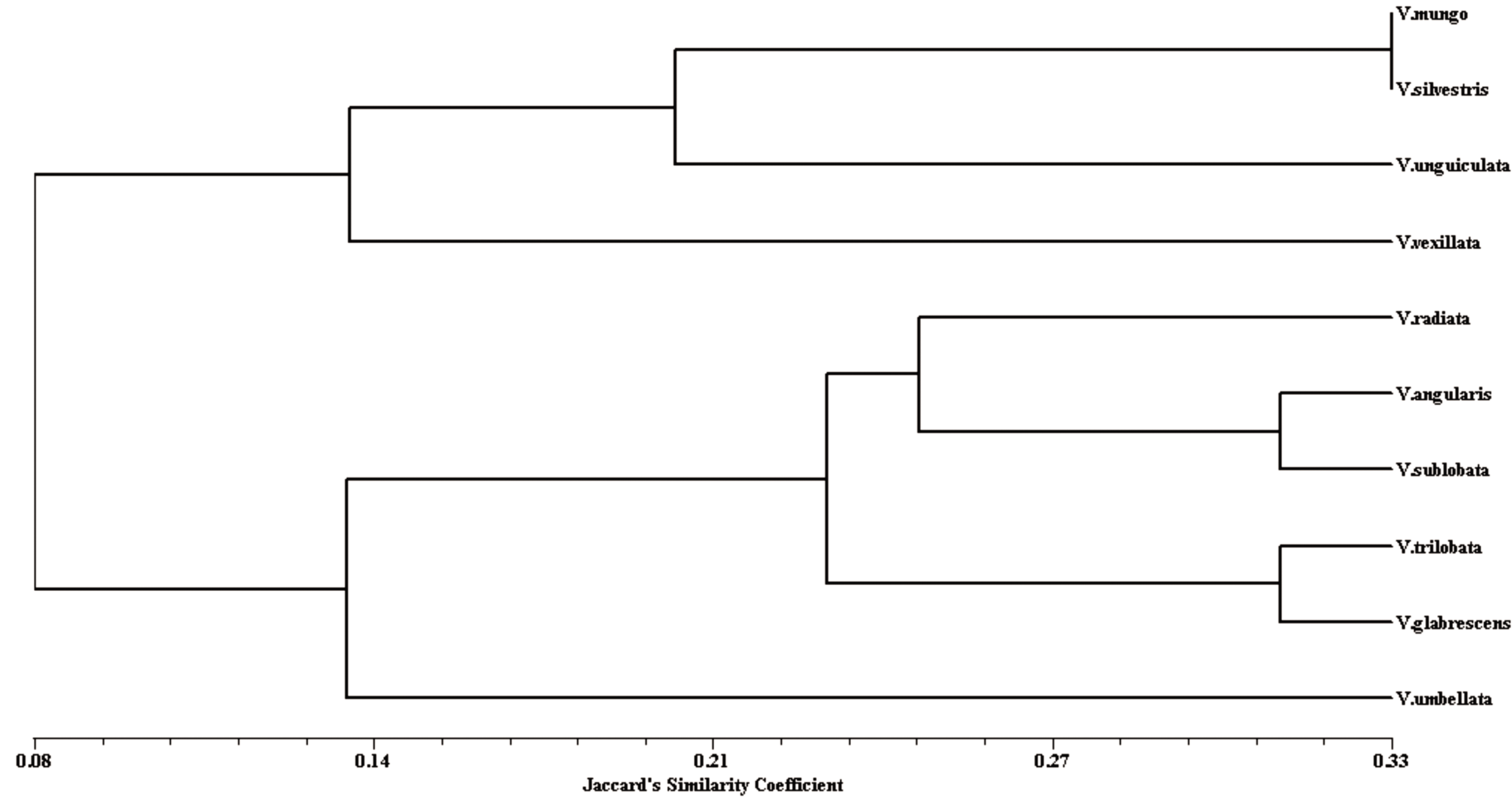

Supplement: S3 Fig — (TIF) [file pone.0128748.s003.tif]
