# Supplementary material for: De novo Assembly, Characterization of Immature Seed Transcriptome and Development of Genic-SSR Markers in Black Gram [Vigna mungo (L.) Hepper]
Source: PLoS One. 2015 Jun 4;10(6):e0128748. doi: 10.1371/journal.pone.0128748 (PMC4456365; doi:10.1371/journal.pone.0128748)
Supplement: S2 Table — (DOCX) [file pone.0128748.s005.docx]

| **Primer code**  **S2 Table**. **Genic-SSR primers designed from transcriptome sequence of black gram**. | **Sequence-ID** | **SSR** | **Forward primer** | **Reverse primer** | **Product size (bp)** |
| --- | --- | --- | --- | --- | --- |
| VMgSSR1 | >CONTIGS_339 | (AG)9 | CATGACCCCATACGGCTAAC | AGTCCCAAAGAAAAGAACCTCC | 279 |
| VMgSSR2 | >CONTIGS_350 | (GA)7 | ACTTCCAAAGCTATATCCGCAA | CAAACCAACCAACCTCTCTCTC | 339 |
| VMgSSR3 | >CONTIGS_778 | (AG)8 | TATCGTAGAGAGAAGGAAGCGG | AATTTACACTTTGGAGGCTTGC | 366 |
| VMgSSR4 | >CONTIGS_745 | (AG)11 | AAGGAAGAAACCCACACAAGAA | TCGATTGAAGAGCAAGAAGTGA | 233 |
| VMgSSR5 | >CONTIGS_1381 | (AG)9 | GACTCAGCGGAAGGAGAAATC | GGACTGGCACCATCAACAG | 279 |
| VMgSSR6 | >CONTIGS_191 | (AG)11 | ATGATCTGCTTCCCATACCATC | AGGAGCTGAAAACCCTTGTCTA | 235 |
| VMgSSR7 | >CONTIGS_196 | (GA)10 | CTATGTAGTCAGAAAGCGCGG | TTTATTGCATGTCGTCAACTCC | 340 |
| VMgSSR8 | >CONTIGS_1533 | (AG)8 | AGATGATAAACACCCTTCCTCG | AACACAAGCCCAGACAAGAAAT | 373 |
| VMgSSR9 | >CONTIGS_2645 | (AG)11 | GATCTCCGAAGGTTGAGAAGAA | TCCAACACACTATTCGCTCTTT | 304 |
| VMgSSR10 | >CONTIGS_3395 | (AG)9 | AGAGAGAGGGAGGGGAGACTAA | GCAATAAGGAGGTTGAGGATTG | 186 |
| VMgSSR11 | >CONTIGS_2933 | (AG)10 | TGTTTGGTAGGGTTTTGGAGTT | TCATTCTCATGTTTGGGATCTG | 300 |
| VMgSSR12 | >CONTIGS_1649 | (AG)10 | GAGAGTTGGTTGAAGGAAAACG | AAGGATCTTGTGAAAGGAGTCG | 373 |
| VMgSSR13 | >CONTIGS_4137 | (GA)14 | AGTATGCCAGAAATCCATGCTT | GGCAGGAAAAGCTCCTTCA | 139 |
| VMgSSR14 | >CONTIGS_5252 | (GA)9 | GGGGAGGAAAGAGAAGAAAGAA | CTGAGAAGAAACAAGGGTGGAG | 123 |
| VMgSSR15 | >CONTIGS_5258 | (GA)8 | GAACTGTATGTAGCAGGGGCTC | AGAGGAGACAAAACGCAGAGAT | 307 |
| VMgSSR16 | >CONTIGS_5301 | (AG)15 | CCCAATAGTACCCTGATTCCAA | CGAGTCCCTAAATAAATCTGCC | 320 |
| VMgSSR17 | >CONTIGS_5241 | (AG)12 | CTGATAGTGATAGCTGTTCTGAGTTCT | GAAGAAGATGGGGATGATTTTG | 196 |
| VMgSSR18 | >CONTIGS_2088 | (CA)8 | TGCATGTCACCATAATAGCCTT | TCTTGGAGCTTTTGGAGGAA | 348 |
| VMgSSR19 | >CONTIGS_3817 | (CA)6 | TGTTGAGAGACTGCTGCTGATT | TATGCTGTGTGTGCCTTCTTCT | 383 |
| VMgSSR20 | >CONTIGS_3092 | (CA)6 | GGGCATTGGTAACTCTTGAGAC | TGTTTCTTGGTCTTGATTCGTG | 242 |
| VMgSSR21 | >CONTIGS_1565 | (CA)6 | GAACAGTGAGAATGAGGAAGGG | ATTTGAGGTTGGAGCAGATTGT | 245 |
| VMgSSR22 | >CONTIGS_3817 | (CA)6 | TGTTGAGAGACTGCTGCTGATT | TATGCTGTGTGTGCCTTCTTCT | 383 |
| VMgSSR23 | >CONTIGS_5215 | (CA)6 | TCGTTGGACCTAGTAACCTTGT | AATTGGTACATCCTCCATCATC | 384 |
| VMgSSR24 | >CONTIGS_7975 | (CA)8 | CTCTGAATTGGCTTCTGGTTTC | TTTTGTTATGGGTCGGTGTAGG | 277 |
| VMgSSR25 | >CONTIGS_9236 | (CA)9 | AGAAAGATTCGCAGTGACAACA | ATCTAATCGGTGTTTGGTTGGT | 388 |
| VMgSSR26 | >CONTIGS_18222 | (CA)8 | ACTTGAGTTCGAGAGGTTCGAT | GTATTCGCCTAAGTTTGAACGC | 198 |
| VMgSSR27 | >CONTIGS_24716 | (CA)8 | AGGTCGTAGAGAATGACGCTGT | CTCTTCACACACAGACCAAACC | 231 |
| VMgSSR28 | >CONTIGS_25905 | (CA)7 | GTCACTGGAGGGGAGCTGT | TGATGGAAAGGTGTGAGAGAAA | 226 |
| VMgSSR29 | >CONTIGS_119 | (AT)6 | CAACCCCATCTCTCATTTTGTT | GTTTGCTTGTGTCGTTTTGAAG | 345 |
| VMgSSR30 | >CONTIGS_88 | (AT)10 | GCCAATCTTATATCCAGTAACC | CTCTTAATGTAACTACTCGGCA | 205 |
| VMgSSR31 | >CONTIGS_154 | (AT)6 | ACTGCTGGAAACCAAGAAATGT | ACAACGAAGGCAAGTACAACCT | 298 |
| VMgSSR32 | >CONTIGS_161 | (AT)7 | CCAACACCACCCAACTAATTTT | TTACATGCAGAGGAGTACCCAA | 236 |
| VMgSSR33 | >CONTIGS_241 | (AT)6 | CTTCTGTTGTCCACCATTCTTG | CCGTGCTAGTGAAACGAATTTT | 278 |
| VMgSSR34 | >CONTIGS_307 | (TA)10 | AGAGGGGTAAAGCCATGTGTTA | GGGATTATAGAGGACGAAAGGG | 195 |
| VMgSSR35 | >CONTIGS_758 | (AT)12 | CATGGAATGTGTTTGTCGAGAT | GAAAGCCTCAAAAGTCTCCCA | 396 |
| VMgSSR36 | >CONTIGS_838 | (AT)6 | CACTATGCGCCATCTAAAACAA | TCTCCTTGGAAATCACACTTCA | 274 |
| VMgSSR37 | >CONTIGS_1064 | (TA)6 | TCAGCTCAACAACACAACACAA | AAATGAATATAGCGGAGGATGC | 249 |
| VMgSSR38 | >CONTIGS_193 | (TA)8 | CTTGGCTTAGTTCAAAGTTGGG | CCAATCAGGTAAGGACAACACC | 391 |
| VMgSSR39 | >CONTIGS_1867 | (TA)9 | AAGAGAAGAAAGGGAATACCGC | GCCTATTATTTTGCTACACGCC | 372 |
| VMgSSR40 | >CONTIGS_2244 | (AT)6 | AGTCAGATGCAGACCAAACCTT | AGATTTCACGAGATGGAAGCAT | 363 |
| VMgSSR41 | >CONTIGS_3455 | (AT)11 | TGGAAAGAAGGAATGAGTGAGA | TCACAAGTCATCAAAACTCTGC | 240 |
| VMgSSR42 | >CONTIGS_3210 | (AT)7 | TCATAGTGGTTGAATGGAATGC | GAATGTAGGCTCAGTTGTGCTG | 369 |
| VMgSSR43 | >CONTIGS_4384 | (TA)6 | CGGTATGAAAGAATGAAGTCCC | CCATAGACAACCAAACATCCAA | 241 |
| VMgSSR44 | >CONTIGS_4566 | (AT)8 | GAAGAAAAGCAACAACAACGC | CCACCAAACCTAAAACACAAGA | 264 |
| VMgSSR45 | >CONTIGS_4015 | (TA)7 | ATCTTGGCACTTACACTGAGCA | CCTGAGAGAGGAGCAAACAAAA | 380 |
| VMgSSR46 | >CONTIGS_4788 | (AT)6 | GGACCGTTGTTCTGGAATCTTA | GCTATGCCCGTAATTTCAACAG | 323 |
| VMgSSR47 | >CONTIGS_88 | (TG)6 | GCCAATCTTATATCCAGTAACC | CTCTTAATGTAACTACTCGGCA | 205 |
| VMgSSR48 | >CONTIGS_241 | (CT)8 | TTTCTCTCCTGCTCCTCTTGC | CTCCGCTCTTAAATCATCGTTC | 234 |
| VMgSSR49 | >CONTIGS_254 | (GT)8 | TATCAGGGCAAAAGTCTGTGTT | TGTAGGTTCATTCACTGCCTCT | 220 |
| VMgSSR50 | >CONTIGS_353 | (TC)8 | TCTTCACACGTAAACACGCTTT | CTCAGAAGGGTACAAAACCCAC | 278 |
| VMgSSR51 | >CONTIGS_394 | (TC)11 | TCTCTGCAACTTCAACCTCTTG | AAGTGGATTCTTACTGGACGGA | 273 |
| VMgSSR52 | >CONTIGS_560 | (TC)18 | TTTCTCAGCGCCTCTCCTT | TGTCTTCAATGCTGTATCCGAG | 309 |
| VMgSSR53 | >CONTIGS_869 | (CT)12 | TAGAACCACCATAACCCCAATC | GAACGAAGCTGGAGAAGTTGTT | 331 |
| VMgSSR54 | >CONTIGS_1032 | (CT)6 | CTTCATGCGGTAAATCTTAGGG | GAAAGGTTAGGGGTACTTTGGG | 247 |
| VMgSSR55 | >CONTIGS_1189 | (TC)7 | TCACAACACAACACAGCACAG | AAAAGTATCCATAGAGGCGCAA | 398 |
| VMgSSR56 | >CONTIGS_1235 | (CT)6 | GACCCCTCTGTTTCCTCTCTTT | ACTTCGACATAGTCCCCAACAC | 275 |
| VMgSSR57 | >CONTIGS_1341 | (TC)6 | CTTGGAAGAGGTAGTTGCCTTG | TCCAGAAATTAGAGGTGTGAGTGA | 220 |
| VMgSSR58 | >CONTIGS_1352 | (CT)9 | GACGAAGCAATTCCAAACACTT | GTGATGCTGACGAAAGTCTCAA | 249 |
| VMgSSR59 | >CONTIGS_5452 | (TC)7 | CACAAAGTGGGATTTACTCTGC | ATACTCCGTAAGACACCGAAGC | 262 |
| VMgSSR60 | >CONTIGS_5468 | (TC)8 | AAGTTCCCCACCACAGAGAAG | AAAAGATGAGGGTGTGCAGTTT | 276 |
| VMgSSR61 | >CONTIGS_196 | (CT)6 | CCCCATGCTAATAAAACCACAT | AGTCGGTCGAATCTTCTCTGTC | 337 |
| VMgSSR62 | >CONTIGS_1701 | (CT)14 | ACAACCCAACCAACTAACATCC | GTGGAGACGTAAATGTATGCGA | 210 |
| VMgSSR63 | >CONTIGS_1732 | (TC)6 | AGATTACAGGATCTGGGTGTGG | TGCCTCAACAAGGGAAATTACT | 353 |
| VMgSSR64 | >CONTIGS_1846 | (GT)8 | AGCAACTACCACATACTGAACGA | CTCTGGCTCAAAGAAATCTCAA | 302 |
| VMgSSR65 | >CONTIGS_2052 | (CT)6 | TCGTGTAAGTCTCTCGCATGTT | GAAACAAGGCAGTACGAAAAGC | 350 |
| VMgSSR66 | >CONTIGS_2054 | (TC)8 | CTGCAAAGCATTCCTGGTT | AAGTAGTGCCTCCACAAAGGAC | 339 |
| VMgSSR67 | >CONTIGS_2799 | (TC)6 | TTGCATATCACTGTTGTCCCTC | AAATAAGCTCCTGCTTGACCTG | 347 |
| VMgSSR68 | >CONTIGS_2280 | (CT)8 | ATGTAGACACGAACGGGTTTCT | AAGGAGAAGAAGCGGTGATACA | 241 |
| VMgSSR69 | >CONTIGS_2428 | (CT)6 | TAAAAGCCTCCGAAACAGAAAG | TCTCTTGATGAACGAAATGCAG | 240 |
| VMgSSR70 | >CONTIGS_2943 | (GT)7 | ACCCTCTTACAGATGTGATGCC | TGTGACGGAGAGAATGATATGC | 278 |
| VMgSSR71 | >CONTIGS_2969 | (CT)7 | TCCACACAAGTCTCAACTGGAA | TGTGAATCCTACATTCCTCCCT | 395 |
| VMgSSR72 | >CONTIGS_3049 | (TC)7 | TGTGTTCGTAATCCATGCTCTT | AGCGAAGCCTATTTTGTTTCAG | 231 |
| VMgSSR73 | >CONTIGS_3412 | (TC)8 | TACCCGTTGTAAGACGAATGTG | GCAAATAACAAAGAGGGGTCAG | 277 |
| VMgSSR74 | >CONTIGS_1565 | (TC)10 | GAACAGTGAGAATGAGGAAGGG | ATTTGAGGTTGGAGCAGATTGT | 245 |
| VMgSSR75 | >CONTIGS_4191 | (CT)6 | TTGATTCCACAGAGTGCCTAAT | TGTAAGAGAAGTTGAAGGAGAGAGA | 301 |
| VMgSSR76 | >CONTIGS_4755 | (TC)7 | TTTAGGTGTCCTTGGTTTGCTT | TATGGTTGGCTTGTCTACATGG | 345 |
| VMgSSR77 | >CONTIGS_3976 | (TC)8 | TCAATACCTTTTCTCTTTCCGC | GGCGAACTCAACTCCTATGGTA | 328 |
| VMgSSR78 | >CONTIGS_124 | (ATC)7 | AAGCAAGCAAAGATCAGAGAGC | TGTTTCCCATGTTCAAGTTCTG | 257 |
| VMgSSR79 | >CONTIGS_338 | (GAA)7 | CACAGCAGAAAGAAGAGAGCAA | AGAAAGTAGAGTGCAGCCAAGG | 117 |
| VMgSSR80 | >CONTIGS_392 | (ATA)6 | GTACCAGTGAACATCGACCAAA | CAACTCAATCGCACTCATTTTC | 342 |
| VMgSSR81 | >CONTIGS_510 | (CAT)7 | TAATAGCAGCAAATCCCTCGAC | CTCCTTCCTATGATCCTTCACG | 348 |
| VMgSSR82 | >CONTIGS_545 | (TCA)7 | GCCAGGATCACATCATACAGAA | GTGCCAACACCAGTAACAAAGA | 283 |
| VMgSSR83 | >CONTIGS_885 | (TCT)9 | AATATCCAATCCGTCTGGTGTC | CTGTGATTTCCTTTCCTTTTGG | 182 |
| VMgSSR84 | >CONTIGS_1038 | (TTG)7 | GGAGATACGGTCAAATGTCCAG | ACAACACCACCACCACCAC | 149 |
| VMgSSR85 | >CONTIGS_1463 | (CGG)7 | CAACCCTAACCACTCCAAAGAC | CTTAGCAATAAGCGGTGCATTT | 380 |
| VMgSSR86 | >CONTIGS_1833 | (TCA)8 | GCAGGAAAACAAAGAACTGTGA | TGCAAGGAAGTTGGTAAAGTGA | 352 |
| VMgSSR87 | >CONTIGS_2026 | (CAA)19 | AACAACAGCAACAGCAGCA | GTAAAACTTGCCCAGAAGGTTG | 324 |
| VMgSSR88 | >CONTIGS_2215 | (ATC)6 | GCTGCCACAGACTCACATAGTT | AAACCAAACTGGACTTTACGGA | 340 |
| VMgSSR89 | >CONTIGS_2342 | (ACC)7 | CATCATCAAACTCCTCGTCAAA | CAACCAGAACAACAAAGGTCAA | 380 |
| VMgSSR90 | >CONTIGS_2644 | (CTT)8 | CGCAATATGACCCTTCTTCTTC | CTCCATCAGGTAGATCCCAGTT | 393 |
| VMgSSR91 | >CONTIGS_2708 | (TGC)6 | AATCATCCTTTGCAGGAGTTTG | TGTCAGTGGATTTGGATTTCAG | 332 |
| VMgSSR92 | >CONTIGS_2822 | (GGA)6 | CCAGAGTGTGCAAGATAGGAGA | TCAAGAAGGTTCACAGTTTTGC | 340 |
| VMgSSR93 | >CONTIGS_2916 | (GGC)6 | TAGGAAACACCCTCATCTCGTT | CAAATCAAGTTCGACATCCTCA | 328 |
| VMgSSR94 | >CONTIGS_1565 | (GAA)10 | GAACCTCCAGATCAAAACCAAC | AACATCGTTAAGCTCGAATGGT | 385 |
| VMgSSR95 | >CONTIGS_3367 | (AGA)7 | TCCTCTCTTCTTCTCTTTCCCC | CAAACCCGTAACTCTGGATCTT | 256 |
| VMgSSR96 | >CONTIGS_3432 | (ACC)7 | CAAAAGCAACAACACATGGTCT | AAGGTCAAGGTGGTGGTAGTGT | 384 |
| VMgSSR97 | >CONTIGS_3607 | (TCA)7 | TTTAACCTCCAATGCTCCCTTA | CCTTCTTTTGCTTGAGTTCCAT | 342 |
| VMgSSR98 | >CONTIGS_3730 | (GCA)6 | TCCCTACCAACATGAAGAGACA | TCATCAATCACACCTCTCAACC | 307 |
| VMgSSR99 | >CONTIGS_4142 | (TGC)6 | TGGTAGGTTCAGTTTTGTGTGG | TTGGTGTGCCTTATGGACTATG | 361 |
| VMgSSR100 | >CONTIGS_4549 | (CTC)8 | CTTGCTTCACATCCTCACAAAC | TGGCTTGTCTCTACATTCATGG | 306 |
| VMgSSR101 | >CONTIGS_4881 | (GAA)8 | GGCAGAGAACAACAACATCAAA | CACACTCCAACCAACATCACTT | 375 |
| VMgSSR102 | >CONTIGS_4905 | (CAT)7 | TGTCGGATTGGATAATGAACAG | TGCAATTCCTGCAACCAGTA | 177 |
| VMgSSR103 | >CONTIGS_4932 | (AGA)9 | TGTGGGAAAGAGAAGAAGAAGG | AGGCGTTCAGTCATAATCCAAC | 325 |
| VMgSSR104 | >CONTIGS_5181 | (GAT)7 | TGAAATGATGTTGGAGGATGAG | TTTGGATAGCAGACATAGGCAC | 380 |
| VMgSSR105 | >CONTIGS_5330 | (ACA)6 | AACGACCACAGCAAGTCCTAAT | GGCTGAGAAGCAGAAGAAGAAG | 354 |
| VMgSSR106 | >CONTIGS_5638 | (CCG)5 | TGGAGAGAGAGGATTTGGATGT | TTGAATTGAGTGGGAGTGAAGA | 179 |
| VMgSSR107 | >CONTIGS_4028 | (ATG)7 | ACAACAAGCACACAACGAGAGT | ATCATCCAATAATGCTCCCATC | 363 |
| VMgSSR108 | >CONTIGS_109 | (AG)6 | AAGCTGAATGGTATGCTGTTGA | CACTTTGATTTCCCATCTCCAT | 221 |
| VMgSSR109 | >CONTIGS_599 | (GA)7 | CCCAGTGCCAACAGCACA | GGGATTGATGACGGAGAAATTG | 115 |
| VMgSSR110 | >CONTIGS_823 | (GA)6 | TTGGAGAAAGAGAGAAGCCAAG | TTCATCAACACAAGGCGTAGG | 196 |
| VMgSSR111 | >CONTIGS_921 | (GA)7 | GGTGAAGAGGAATACGAGGATG | GTGCTATCCAACCAACACCAA | 337 |
| VMgSSR112 | >CONTIGS_1443 | (GA)6 | ATCTCCAAAGCTACCCTTCTCC | ATGTTTAATCCAACGACCCTCA | 242 |
| VMgSSR113 | >CONTIGS_5395 | (AG)6 | TTCGGCTACAGTGAGTGAAAGA | TGAATCGAAAGAGTTACCGACA | 277 |
| VMgSSR114 | >CONTIGS_1870 | (GA)6 | CAAGACAGGCTACTGAGGAACA | TTTATCCCTTTCACCTTGCAGT | 191 |
| VMgSSR115 | >CONTIGS_1979 | (GA)6 | TACTGGCTACCTCTTTGGGTGT | TCTCTCTCCCACCTTCTTCTTG | 300 |
| VMgSSR116 | >CONTIGS_4071 | (AG)6 | AATGCTGCTGAAGAACAGAACA | GAAAAGGATAATTGCACAAGCC | 389 |
| VMgSSR117 | >CONTIGS_4080 | (AG)7 | TTATCGCTCTTATTCGTCGGTT | TTTACTGCTCCGTGTTATTCCC | 168 |
| VMgSSR118 | >CONTIGS_4330 | (GA)6 | AATGGCTAGGGACACAAAACAC | GACCCTGATTTTACATGGGAAG | 249 |
| VMgSSR119 | >CONTIGS_3497 | (AG)8 | GTTTGGTTAGAACAGGCTCCAG | ATTTCTCTGCATCTCCCACATT | 231 |
| VMgSSR120 | >CONTIGS_3430 | (GA)7 | GGGAAGGTTCTATTCCATTTCC | TTACACTGCTCTTTCGCTCTTG | 195 |
| VMgSSR121 | >CONTIGS_5677 | (GA)6 | CCATGTGTTATTTCGTCTGCAT | AGCCTTTCCTCCTCCAATACTC | 183 |
| VMgSSR122 | >CONTIGS_13076 | (CA)6 | GCAGGTACTGGTGTAGAATGATATG | GTTCCCGATGTGTGTTTCTGTA | 159 |
| VMgSSR123 | >CONTIGS_16401 | (CA)8 | GACAACCAACATACATCTTCCAG | CCTCTCCTGCATCTTTCTCTCT | 135 |
| VMgSSR124 | >CONTIGS_25047 | (CA)7 | ATGTCTCTGTCTCTGTCACTCTTTCT | TCTGTTCAGGAGCTGTGTGAAT | 101 |
| VMgSSR125 | >CONTIGS_649 | (AT)11 | GGTCAGATTAGTACGGGCAGAA | CAGCATCATCTTCCTTCTCTCA | 127 |
| VMgSSR126 | >CONTIGS_3467 | (AT)8 | AGCTCCCTCCCAAATTACAAA | GGTTCAAAGAGGACAAAGAAGG | 166 |
| VMgSSR127 | >CONTIGS_4200 | (AT)6 | TTCTCTCCTCGACTATCTTCGC | AACACAGAATATCACCACGCAT | 118 |
| VMgSSR128 | >CONTIGS_431 | (TC)7 | CCTAGTCCTCCAAAGCCTCTCT | TGCCTTCTCTCTCCTTTCTTTCT | 384 |
| VMgSSR129 | >CONTIGS_624 | (TG)8 | GGCCAAGGGAGAGAAAGATAGT | AGTGGTAGGTGAAGGAAACCAA | 137 |
| VMgSSR130 | >CONTIGS_642 | (CT)8 | TACAAAACCCTAATTTGCCTCC | TCTCTATCACTTGCCTTGACGA | 176 |
| VMgSSR131 | >CONTIGS_694 | (GT)6 | TAAGGTCAGAAGAAAGGCAAGG | GCAGAGCATAAGAGAAGGCAAT | 107 |
| VMgSSR132 | >CONTIGS_701 | (CT)6 | CCAGAAACACACCCTCTTCTCT | CAATTCCTCCACACACAGAAAC | 126 |
| VMgSSR133 | >CONTIGS_802 | (TC)6 | ATCATTCCCACCCCTATCCTT | AGCAGAGAGTTTGACCTTGGAG | 273 |
| VMgSSR134 | >CONTIGS_805 | (TC)6 | TCTTCGCTTCTCTCTCTACGCT | TTCCACAGTCTCCACATTGAAC | 256 |
| VMgSSR135 | >CONTIGS_816 | (CT)6 | CCGCACACATACACATTCTTTC | CATTTCTCCTCCTCCTCACG | 172 |
| VMgSSR136 | >CONTIGS_1513 | (CT)5 | TCGAACTGGTAGGTCTGGAAGT | CATAACGCCATAGGAGAAGGAC | 335 |
| VMgSSR137 | >CONTIGS_1475 | (TC)5 | GAAGAAAGTGGTGGGGATTGTA | TTCGAGAGAGAGAGAGAGAGCAA | 243 |
| VMgSSR138 | >CONTIGS_1465 | (CT)5 | CACATTAGGAGCAGTTTTGCAC | GATCTGGTGTTGGTGTCAGTGT | 341 |
| VMgSSR139 | >CONTIGS_2154 | (TC)6 | AAAGAGAGAGAAAGAGAGCGCA | GGGCAAAGGAAGAGAAAAGATT | 204 |
| VMgSSR140 | >CONTIGS_1903 | (CT)6 | GAATGCTCAGGAGACGAGAGTT | GGTCGTTGGAGAGAGAAACAAG | 189 |
| VMgSSR141 | >CONTIGS_2333 | (CT)8 | CATTTTCTTCGCTCTTCTCTCT | AGTATCCCAAACCCAGAACTTT | 157 |
| VMgSSR142 | >CONTIGS_2847 | (CT)8 | AATGTCTCCGTTATCGAGGGT | AACACCAAGAAAAGCGAGGAG | 151 |
| VMgSSR143 | >CONTIGS_3648 | (CT)6 | CCCTGTTTGTCTCTTTCTCTGG | GATAACCTTGGTGGAAGAATCG | 136 |
| VMgSSR144 | >CONTIGS_4809 | (CT)14 | CTTCCAGATCGCTTTGTTCTTT | ATTGGAGGTTGAGTTGAGGTGT | 193 |
| VMgSSR145 | >CONTIGS_4498 | (CT)8 | CTCAGTCGATTTGCAGTTTCAG | CTGGTTTCTGCCTATACCGAAG | 188 |
| VMgSSR146 | >CONTIGS_176 | (ACA)5 | ACAGTTGCCTTTGTTTGAAGGT | TGTATCCTCAGCCACATAGCAG | 186 |
| VMgSSR147 | >CONTIGS_159 | (TAA)5 | TCAACATCCTCGGTCTCCTATT | TCGTACTCTTGTTTCTGCTCCA | 391 |
| VMgSSR148 | >CONTIGS_267 | (GAT)5 | ATTGATGAGAAGGGGATGATTG | GATTTGAACTTGCACTCCATGA | 331 |
| VMgSSR149 | >CONTIGS_273 | (GAG)5 | CTTCTCAATCTGCTGAACCTCC | GTTGCTGTTGTTGCTCTTCATC | 177 |
| VMgSSR150 | >CONTIGS_348 | (GAT)5 | GGCTGGAATTTGCTGTAGAGTT | AGACAGAGGTGACGCCATACTT | 347 |
| VMgSSR151 | >CONTIGS_390 | (AGA)6 | TAAAAGAAGAGCACGGAAGAGG | GAACAGAGTTGGGCTGTATTCC | 397 |
| VMgSSR152 | >CONTIGS_391 | (GAT)6 | AGACTCAAGGAAGGTGAAGCAG | GAGCAGCATCAAGTGACGTATC | 253 |
| VMgSSR153 | >CONTIGS_433 | (TGC)5 | CTAATGAAGGAGGAGGAAGGGT | GAATACGGTTCCAGTAAGGTGG | 246 |
| VMgSSR154 | >CONTIGS_439 | (CGT)5 | ATATCATCAGCATCACTGCCAC | TACAGACTCATTTGTTGGGCTG | 225 |
| VMgSSR155 | >CONTIGS_556 | (TCA)5 | TTGGACCGTGTGATTACTTTCA | GAATCTGTGGGTGGAATTTGAT | 240 |
| VMgSSR156 | >CONTIGS_599 | (GCC)5 | CGGTAGCGTGAGGAATGATAGT | GCCAAGCAGGATACAGTTCAGT | 338 |
| VMgSSR157 | >CONTIGS_649 | (TTG)5 | CTTGGAAGAGTCAAACAACACG | GGAGGATGAACAAGGTGAAGAA | 356 |
| VMgSSR158 | >CONTIGS_662 | (GAC)5 | TCCCTTCCAAAATAGTGTCAGC | GTGAGAAACGAGGAGAAGACGA | 112 |
| VMgSSR159 | >CONTIGS_729 | (TCG)5 | ATACTGGCAAGGAAGGATTTCA | AACCCTAAACCTAAACCCCAAA | 197 |
| VMgSSR160 | >CONTIGS_771 | (CTG)7 | TATTTAGATGCGTCCTGTTTGC | CATGTCTCGCCATAACAACACT | 148 |
| VMgSSR161 | >CONTIGS_819 | (GCA)5 | GTGGGAGTGAAAGAAGAAAGCA | TTTTCGGTAACTTCAATGCTCC | 220 |
| VMgSSR162 | >CONTIGS_894 | (CTC)5 | AGAACTACCACCACCACCAAAG | TGCCACTGAAGATGAAATCAAG | 268 |
| VMgSSR163 | >CONTIGS_905 | (GCA)6 | TATTCATGTTACTTCCCACCCC | TGAACGAAGAGAACCCCATAAC | 383 |
| VMgSSR164 | >CONTIGS_911 | (GAA)5 | CTGGAGGATTGGACTCAGAAAC | ATCACATGCACCTTCAAAAGC | 394 |
| VMgSSR165 | >CONTIGS_1040 | (GAA)5 | TGTGATTCTCTCTCCCTCTTGA | GATATTCTCTCCCATCTGCACC | 148 |
| VMgSSR166 | >CONTIGS_1044 | (CCT)5 | CATCATCCACTGAAGCAAATGT | CTGCTGAGAGATTCCATTACCC | 336 |
| VMgSSR167 | >CONTIGS_1048 | (GCT)5 | GGTCGAACTTTCAGCTCTAGGA | CTTCAGCGTCTCCATTTTCAC | 245 |
| VMgSSR168 | >CONTIGS_1086 | (GTT)6 | AGAAAATTGGAGAAGCAGGACA | AGCTTGCAGTGAGCCAAGAT | 397 |
| VMgSSR169 | >CONTIGS_1149 | (AGC)5 | CAAGCTCAAGAAGCTCGGTATT | GCAAGAGATAAATGGGGTGAAA | 264 |
| VMgSSR170 | >CONTIGS_1156 | (CCT)5 | CAATCCTTACCAACAACAGCAA | CATCACCACCCATTACTCTTCA | 233 |
| VMgSSR171 | >CONTIGS_1161 | (CTG)5 | AAGAGAAGTGGATCGGTTGAAG | CTCTGTAGGATCAGGCTCAGGT | 216 |
| VMgSSR172 | >CONTIGS_1162 | (TCC)5 | ATCTGCAACCACAAACCTTCTT | GCGTATGACTTCACAACTCTGG | 288 |
| VMgSSR173 | >CONTIGS_1193 | (GAG)5 | GGCGTTCTAATCGAGACCATAG | GGAATTGGTGAGACAAGAGACC | 176 |
| VMgSSR174 | >CONTIGS_1229 | (ATC)5 | TTCATTCACCCCACTCTCCTAT | AGGGAAGGGAAATATCGAAAGA | 169 |
| VMgSSR175 | >CONTIGS_1230 | (ATT)6 | GTGTGAAAATCAAAAGGGCTTC | AGCAGTGATCCAGTTATGACCC | 396 |
| VMgSSR176 | >CONTIGS_1253 | (TGG)5 | GCAACTGCATCCAATACCATAG | TGGGAGTTTCTGTCTGATGATG | 269 |
| VMgSSR177 | >CONTIGS_1331 | (CAA)5 | TCGCTCATGGAGTATTTGAATG | GGAAGAGGAAGAAACACAATGG | 260 |
| VMgSSR178 | >CONTIGS_1337 | (CAT)5 | CAATTTCTCTGCTTCTTGGTCA | TGCATCAGCCAACTCTGTAGAT | 237 |
| VMgSSR179 | >CONTIGS_1378 | (AAG)6 | AACAGAGTCGAAAGCTGATAGTGA | CTGAAGAAGAACATGACCGTTG | 325 |
| VMgSSR180 | >CONTIGS_1392 | (TGT)5 | ATAGGGCAGTACGTGGACAAAG | AGACAAGGGAATGGAATCATCA | 336 |
| VMgSSR181 | >CONTIGS_1416 | (ACC)5 | GCCAAGCAAATTAGACCAAATC | GCACCTTGCCTTATCTAGCTGT | 248 |
| VMgSSR182 | >CONTIGS_5371 | (CAT)5 | CTTCATCATCCTTGTCAGCAAC | CTGCCTAAACCTCCCACTCTAA | 364 |
| VMgSSR183 | >CONTIGS_5425 | (TCC)5 | CATCAACGCTGCTCTGTAACTC | CTTTTCTATTCGCTCAAATGGC | 378 |
| VMgSSR184 | >CONTIGS_5432 | (TCC)5 | CCCTGGTTACTATTCTGGTGCT | GCCAGTAGGAGTTGTTTGGTTC | 324 |
| VMgSSR185 | >CONTIGS_222 | (CTT)5 | GCCCTTCAAACTCAAAAGAAGA | ACAGCCTTTCTGTCTCCAACTC | 348 |
| VMgSSR186 | >CONTIGS_222 | (CTG)5 | AGAGTTGGAGACAGAAAGGCTG | ACCGACAAAACACGAAGAAACT | 208 |
| VMgSSR187 | >CONTIGS_1445 | (GAT)5 | CCCAGGAACAGTATCAGCCTAC | ACTTTGAGTTTTACGAAAGCCG | 214 |
| VMgSSR188 | >CONTIGS_1462 | (TTC)5 | CACTCTGGATAAATACCACCACAA | CCCATACAGGAGAAAACGAGAG | 266 |
| VMgSSR189 | >CONTIGS_1466 | (GAA)5 | TTAAAGCGATCTGGAATAGGGA | GACATGGAGTTGGTTGACAGAA | 187 |
| VMgSSR190 | >CONTIGS_1496 | (GGA)5 | GGCCCTTTTGTGTCTGATTCTA | TGTGTAGGGTCTCTCCTTCTCC | 152 |
| VMgSSR191 | >CONTIGS_1508 | (GCT)5 | GTAAAGCCATCCAGATTCCAAA | AACGAGAAGAGGACAAACAAGC | 159 |
| VMgSSR192 | >CONTIGS_1658 | (GGA)5 | TATAAGCGGATTGATGCGAGTT | CATCTTCTTCTTCTCCAGCACC | 345 |
| VMgSSR193 | >CONTIGS_1735 | (ATC)5 | GATTAGGAACCTTTGAGGGCTT | CTTGTTGATAACCGGGAAAGAG | 256 |
| VMgSSR194 | >CONTIGS_1736 | (CTT)5 | TCCGAGAATAGTAACGCTCGAT | GTGACTGTGCGAAAGAAGAGC | 151 |
| VMgSSR195 | >CONTIGS_1766 | (CTG)5 | AATGTGAAGCGTGGTGACATAG | CTCGAACCTCTGGATTAAGCAT | 280 |
| VMgSSR196 | >CONTIGS_1768 | (AGC)5 | CTTTTCCATTTCTGTCCAATCC | CTACTACACACTTCACTGGGCG | 280 |
| VMgSSR197 | >CONTIGS_1834 | (GTA)5 | GCTTCATTCTGCTAGTTGGTGA | GACTCTTTCTTGGCTGCTTCAT | 196 |
| VMgSSR198 | >CONTIGS_1911 | (AGC)5 | AGTCTCCTACCAACTGTCCTGC | CATCTTCGTCCTCACCTTCTTC | 174 |
| VMgSSR199 | >CONTIGS_1911 | (GAG)5 | AGTCTCCTACCAACTGTCCTGC | ATCATCCAAGCAACCATCATCT | 191 |
| VMgSSR200 | >CONTIGS_1913 | (TAT)8 | ATAAAGCTGTGCGAGGAAGAAG | TCAAGAAATGGAGTGTTCCAGA | 374 |
| VMgSSR201 | >CONTIGS_1935 | (AGG)5 | ACCTGTCTCAACCACTTCCTTC | AACCTTTCCCAGAGGATTCATT | 275 |
| VMgSSR202 | >CONTIGS_1967 | (TGA)5 | GCCCCATAAATATCAGCAGTGT | TCTATCAAGACAAAGATCGGCA | 360 |
| VMgSSR203 | >CONTIGS_1984 | (GCT)5 | AAGAGCAAGGAGAGAATCGAGA | GCTGGGACACCTTCTTATCATC | 264 |
| VMgSSR204 | >CONTIGS_1985 | (GTG)6 | AACAAATGTGGTGCTCCTTCTC | ATTGTATGCAGGCGGATAGTTT | 285 |
| VMgSSR205 | >CONTIGS_2080 | (GAA)5 | TATCCTTGCCTCCAATACATCC | GAGCCCTAACACCTTCATCATC | 189 |
| VMgSSR206 | >CONTIGS_2106 | (ATC)5 | GACACAGAAAAGAACGGCATTT | TCATCTGCTGCCACAATACTCT | 157 |
| VMgSSR207 | >CONTIGS_2237 | (GAT)6 | AAGAAGCAACTCGAAAGTGAGG | AAGCACTCAGAAACAGACATGG | 127 |
| VMgSSR208 | >CONTIGS_2251 | (GAT)5 | TGAAGAGCCTGATTGAAGATGA | ACTCCACTCCAAAAGTATCCCA | 371 |
| VMgSSR209 | >CONTIGS_2253 | (CTT)5 | CTTCAATCGCTGAATCGAACTA | CCAAATATCAACCTTCCTCCAA | 378 |
| VMgSSR210 | >CONTIGS_2305 | (TTC)6 | TGCTGCTTGTCTTTGTTCTTGT | TTAAGGTTGGAATCTGGAAGGA | 360 |
| VMgSSR211 | >CONTIGS_2420 | (GGT)5 | CACCATAGGGCACAAGAATGTA | CCAAATGTGAAGAAGACCCTTG | 174 |
| VMgSSR212 | >CONTIGS_2453 | (TCT)5 | CGATTTACCCAAACATGGTCTT | CGCTATATGTGAACAAACGGAA | 237 |
| VMgSSR213 | >CONTIGS_2454 | (ATG)5 | CAAGTACAAGACAAAAGCACGG | GTTATTGAGAACACAGGCAGCA | 388 |
| VMgSSR214 | >CONTIGS_2491 | (TCG)6 | CGTAACAGGTTTTAGTCCCAGC | TCGTAGAGGATGTGAAAGACGA | 167 |
| VMgSSR215 | >CONTIGS_2502 | (TGG)5 | ACGGAGTTGATGACCAGTCTCT | TCTCAAAACTAAGCACGAGCAC | 344 |
| VMgSSR216 | >CONTIGS_2526 | (AAT)5 | GCCAATCAGTTTACCACATTCA | GGCAAGTCACACCAACAATAAC | 282 |
| VMgSSR217 | >CONTIGS_2542 | (TTA)5 | ACTCCTCGTCAAAATCCAATGT | AGAGAACACCAAATCCATGCTT | 198 |
| VMgSSR218 | >CONTIGS_2562 | (GAA)6 | GTGGTGGAAGAATTGAAGGAAG | TGATTAGCAACTGGCACACTCT | 203 |
| VMgSSR219 | >CONTIGS_2590 | (TCG)5 | TTCATTATCTTCCTCCTCAGCC | GATGGTTTCGATTTGTCCACT | 174 |
| VMgSSR220 | >CONTIGS_2598 | (CCA)5 | TACTGTTGATTGGGTGCAAAAG | CACCATACGAGTTCTTGGTTCA | 303 |
| VMgSSR221 | >CONTIGS_2643 | (CTT)5 | TTAGAGTGGTTCATCGCAGAAA | CTGCCTTCCCTTTATCCTTCTT | 390 |
| VMgSSR222 | >CONTIGS_2723 | (CTT)5 | CCTTCTCTAGCCCCAAATTCTT | CCTACACCACACCTTTCTCTCC | 328 |
| VMgSSR223 | >CONTIGS_2726 | (TTC)7 | AAGGTTAATGGGAGCCAAGAAT | TACCTGGAGTGAAGGGAAAAGA | 314 |
| VMgSSR224 | >CONTIGS_2858 | (CTT)5 | TGTAAGAACCCAACCAAATCCT | TGCCAAGAAGATAAGATAGGCA | 349 |
| VMgSSR225 | >CONTIGS_2891 | (TGG)5 | AAGCAACAGAAGCAAGGATAGG | AGTCCTCCCTGAACCTCCATA | 272 |
| VMgSSR226 | >CONTIGS_2899 | (TCA)5 | GCTCTTCCACGTAATGCTTCTT | CTGTTATGGACAAGGGTGTGAA | 255 |
| VMgSSR227 | >CONTIGS_2994 | (TTC)5 | ATCATAGTGTGCGGGAAGAGAT | TTCACCTACTTGCGATTGAAGA | 297 |
| VMgSSR228 | >CONTIGS_3051 | (AGA)6 | ACAAAGTCCCCGTATATGCAAG | AGCCTGCTTTACTTCCTCCTTT | 377 |
| VMgSSR229 | >CONTIGS_2971 | (TTC)7 | CCTGTTCCCCTTTCCTCTATTT | GGGGTTCTTGAGGTTGTACTTG | 128 |
| VMgSSR230 | >CONTIGS_3418 | (TCC)5 | GGAGGAGAAAGAGAAAATGGGT | TGTGAGGAGAAGAAGATGACGA | 147 |
| VMgSSR231 | >CONTIGS_1573 | (GGT)6 | CTTCTTTGACTCGCTCTCTTCC | GGCTCTTGCCCTCATAACTAAA | 321 |
| VMgSSR232 | >CONTIGS_1622 | (ATC)6 | GTGTCCCTCCCAATTCTATGAC | AAGGAAGTTGAAGCTAAGAAGCC | 340 |
| VMgSSR233 | >CONTIGS_1628 | (TGA)5 | AAGATAGGAGCGTCTGTGCTTC | CCTACATTAGTCACCCCACGAT | 286 |
| VMgSSR234 | >CONTIGS_3195 | (TCA)5 | CACAAAGTTATGAGAAGCCACG | GCGTGATTTGAGGAGGTTAGTT | 348 |
| VMgSSR235 | >CONTIGS_3224 | (TGG)5 | AGAGGAGTCTGGCCCTAAGAGT | GGGTAAATCCTTCCCCAAATTA | 307 |
| VMgSSR236 | >CONTIGS_3296 | (CAC)5 | GCACCATAAACATCATAAGCGA | ATGAACAACGACACACAAGAGG | 392 |
| VMgSSR237 | >CONTIGS_3600 | (GAT)6 | CTCCCTAACATCATCCACCAAT | ACTGCCTCAAAAGTCTCTCCTG | 176 |
| VMgSSR238 | >CONTIGS_3624 | (CAT)6 | GAAAGGGAAAGGGATAAGGATG | AGAGTGTAGTGCAGAGAGCACG | 382 |
| VMgSSR239 | >CONTIGS_3654 | (TTC)7 | GTGAAGAGGTTTCGATCTCTCG | GTGGGTTCAATTTCTCAGCTTT | 192 |
| VMgSSR240 | >CONTIGS_3667 | (CAG)5 | GATGTCACCAAGAAGTGTGGAA | AAGCTCGTCTCCGTTTACTGAC | 226 |
| VMgSSR241 | >CONTIGS_3781 | (GGA)6 | ACAAATGATAGAGGACGAGGGA | CACTTTGGAGAGAGATGGAACC | 114 |
| VMgSSR242 | >CONTIGS_3842 | (GGC)5 | GTGTCTGCATTGAGTGGTCCT | CAGGGGACAAAACTGAAACATT | 213 |
| VMgSSR243 | >CONTIGS_3136 | (GCT)6 | ACCACCAGAAAATGGAACAACT | ATGGAGAGTGTACCCAGGAGAA | 235 |
| VMgSSR244 | >CONTIGS_4050 | (CGA)5 | TCTCCATTCTCTTTCCCTAACG | CTTGTTGAATCCGTAAGACGTG | 303 |
| VMgSSR245 | >CONTIGS_4074 | (GAA)5 | GGTTTGGTTGTCTAGGAACTGG | ATCGACTTTGGATCTTGAGGAA | 198 |
| VMgSSR246 | >CONTIGS_4093 | (CAT)5 | TTCGAGGGTTTGGTCTTGTAAT | AAGAGGGTTTGGAGATTTTGGT | 369 |
| VMgSSR247 | >CONTIGS_4133 | (ACC)5 | CCACGGTCTCTACTACCTCCAT | GAGAGGATTTGGATTTGTGACC | 351 |
| VMgSSR248 | >CONTIGS_4143 | (CCT)5 | TCCCTTTCCTTCTTCTCGAAAT | GAGCCTGGAGTGTTTGTTGAG | 348 |
| VMgSSR249 | >CONTIGS_4190 | (AGC)5 | TTCTCATCATCCTCCTCCTCTC | CTCCACACTTACAACCTCACCA | 273 |
| VMgSSR250 | >CONTIGS_4206 | (AGC)5 | TTAACAGCTCTGGCCCTTTTAC | AATGCTTCCATTGATGTACGTG | 349 |
| VMgSSR251 | >CONTIGS_4421 | (TTC)6 | GTTTCCCCGTGGTATTCTCTTT | CTTCTGCTCCTGTTCCTTCAGT | 183 |
| VMgSSR252 | >CONTIGS_4430 | (ACC)5 | CAGTTCCTGCACCACCATATC | GAGGTGATCCTTCTCTTGGGTA | 240 |
| VMgSSR253 | >CONTIGS_4454 | (TTA)5 | AACTCCTTCTCTTCTGCCATCT | CTGCTTTCTCAGGCCAAACT | 262 |
| VMgSSR254 | >CONTIGS_4499 | (CTC)5 | CTCCTTCTCTCTTCCTTCCCTC | ACGTTCCTAATGTGTTTGAGCA | 271 |
| VMgSSR255 | >CONTIGS_4515 | (GCA)6 | CTCTCACTTTCTCTCTCACCGC | AACCGAGGGTGGTTCGTAG | 314 |
| VMgSSR256 | >CONTIGS_4519 | (TAA)5 | CTGCTTCAGCCAAGAATGTAAA | ATCCCAGGAGAGTGTTGAAAAT | 201 |
| VMgSSR257 | >CONTIGS_4540 | (AAG)7 | CTTTGGGAGTAGGTGGTGAAAG | ACGCGAATCTTCCATTCTACAT | 148 |
| VMgSSR258 | >CONTIGS_4598 | (GCG)5 | AGCGACTTCCTCATCTTCTGAC | TTGTTCCATTCACTCTTTCTGC | 391 |
| VMgSSR259 | >CONTIGS_4625 | (TTC)6 | GGGGCTGTCAAGGGTAGAGTA | CTCACGGTTCTAAGGTTTGGTC | 158 |
| VMgSSR260 | >CONTIGS_4647 | (CCG)6 | GCCTTCAACTTCCTTCTTCGTA | TGTTCTCTCTGCTTTTGCTTTG | 338 |
| VMgSSR261 | >CONTIGS_4726 | (TAT)5 | AACAGAAGCAGCAGTCACAGAG | ACCCAGCCAATAAACATCAATC | 388 |
| VMgSSR262 | >CONTIGS_4881 | (GAT)7 | GCCCTCATCATTATTTGGATGT | ATCTGCTTCACTCCTACCGATT | 262 |
| VMgSSR263 | >CONTIGS_4881 | (CAA)5 | CCACCACTACTCCTCCTAATGC | TGTGAAGATGATGAACCAAAGC | 376 |
| VMgSSR264 | >CONTIGS_4950 | (GAG)5 | GCTTCAAAGAGAGCAGGAAGAG | CCCTAATTTTCCAAGCCTCATA | 386 |
| VMgSSR265 | >CONTIGS_5027 | (GCT)5 | CCCTGAATTGGTATCCTTGTGT | GGGATGTGGAGCAGGAAGTA | 185 |
| VMgSSR266 | >CONTIGS_5040 | (TGA)5 | ATAACAGATTGGGGTTGGATCA | GAGCTTGTGAAGTGATTGTTGG | 382 |
| VMgSSR267 | >CONTIGS_5083 | (CTC)5 | AATGTTGTCCAAAGGTTCCAAG | GTTTGACCCGGATATTTACACG | 203 |
| VMgSSR268 | >CONTIGS_5086 | (GCT)5 | CGAGATTGAGTTCTCTTTTGGG | CTAAACCGGAACAACAACAACA | 325 |
| VMgSSR269 | >CONTIGS_5164 | (GAA)5 | TCAAACAGCCCCAGTTGAAT | GTCGCTAGGAGAGGATGAAGAA | 149 |
| VMgSSR270 | >CONTIGS_5215 | (CA)6 | TCGTTGGACCTAGTAACCTTGT | AATTGGTACATCCTCCATCATC | 384 |
| VMgSSR271 | >CONTIGS_5225 | (CCG)5 | GACATTAACACCAGCCTTCTCC | CCACTTCTTCCATTTCTCCAAC | 365 |
| VMgSSR272 | >CONTIGS_5318 | (CCT)5 | ATCCTCACATACCTCGTCGTCT | GTCAGAAACATGAAAGCCACCT | 344 |
| VMgSSR273 | >CONTIGS_5501 | (GAA)5 | CCAGTTGAATCCTCACCTTGAT | TCAGAGCACCACTCACTCACTT | 222 |
| VMgSSR274 | >CONTIGS_5504 | (CTT)5 | GCCGCTACAGCCATCAAC | CAACTCCTTCCATCATCATCAA | 196 |
| VMgSSR275 | >CONTIGS_5624 | (TAT)5 | CACCTTCTCTTCACATGGTTGA | GTCCGAACAAACCTACAGGAAG | 264 |
| VMgSSR276 | >CONTIGS_5638 | (CCG)5 | TGGAGAGAGAGGATTTGGATGT | TTGAATTGAGTGGGAGTGAAGA | 179 |
| VMgSSR277 | >CONTIGS_5700 | (AAG)7 | TGGTGGAAAGATGAGTGTTTTG | CGGTTGTTTCTGCTCCTACTCT | 173 |
| VMgSSR278 | >CONTIGS_3952 | (CTT)6 | TAATGAGCCAACCTCTGTGATG | GAGGAAGATTTGTGGGTCTACG | 377 |
| VMgSSR279 | >CONTIGS_3988 | (TTC)5 | TTGTACGTTGTCCCTGTTCTTG | TTGAGGGGAATAGTGGGTTATG | 364 |
| VMgSSR280 | >CONTIGS_3989 | (CTT)6 | AAATGCAGCTCTTCTCGATTTC | CAAATACAAGGCTCTTCACTGC | 260 |
| VMgSSR281 | >CONTIGS_3852 | (TGC)5 | AGTTCGTTGACTGATTTGCTGA | GACTCGAAGTATGATGGGTTCC | 382 |
| VMgSSR282 | >CONTIGS_3882 | (GTTT)6 | CTCGGTGAGGAGGCTAAGAATA | CAAGTGATGATGAGGCAGAGAA | 305 |
| VMgSSR283 | >CONTIGS_3904 | (TTC)5 | ACGTACATCCAAAACCAGAAGC | AACACTGCAAAGAGGAGGAGTG | 288 |
| VMgSSR284 | >CONTIGS_3784 | (TCCAC)9 | ACAGGATCTCACTCTGTCACCC | ATGGAATGGAATGGAATGGA | 290 |
| VMgSSR285 | >CONTIGS_2944 | (AGAA)6.5 | CAGAGCAAGACTCCATCTCAAA | CATACACACACACCCACCTCTC | 222 |
| VMgSSR286 | >CONTIGS_1001 | (AAGA)9 | AGGAGAATCGCTTGAACCTG | AGGGTGAGCGTTATGAGGTAGA | 349 |
| VMgSSR287 | >CONTIGS_166 | (TTAT)4 | AGCAGCCACCGCCTCTTC | GGTTTGGGTTTGGAGTTGGAAT | 187 |
| VMgSSR288 | >CONTIGS_134 | (CAGCTG)3 | TGCTTGTCTAAAAGGCTCACAA | GCCCTTATTGCAGTTGGATTAC | 293 |
| VMgSSR289 | >CONTIGS_147 | (GTGACC)3 | AAACTGATGAAAAGACAACGCC | GGAAGGTGTTTGGAGAAGAAGA | 365 |
| VMgSSR290 | >CONTIGS_1079 | (AAGAGA)5 | TAGTTGTTGTCCTCTTGTGCGT | TCCTAGTACCCCATTCCTCTCA | 365 |
| VMgSSR291 | >CONTIGS_1121 | (AAAG)6 | TAGGGAAAGTTCAAAGGCAGAA | AGAGCGCAAAGCTCAACTAAAA | 209 |
| VMgSSR292 | >CONTIGS_1494 | (GAAGA)5 | GGAGGATAGGATGAAGAAGAGGA | GTTGATATTGGGGTTCCAAAAC | 261 |
| VMgSSR293 | >CONTIGS_1634 | (AATA)8 | GCCATGTCCTCTTCTGATTTCT | CTTCCTGGGTTCAAGTGATTCT | 173 |
| VMgSSR294 | >CONTIGS_3189 | (ATTCC)5 | ATTCCGTTCCATTACATTCCC | AGCGGAGTGGTGTCAAATG | 148 |
| VMgSSR295 | >CONTIGS_5468 | (TC)8 | AAGTTCCCCACCACAGAGAAG | AAAAGATGAGGGTGTGCAGTTT | 276 |
| VMgSSR296 | >CONTIGS_5482 | (GCGGC)4 | CAACAAGCTGATCCCATGATAA | CTAACTCCGTCGTACAGGCTTC | 196 |
| VMgSSR297 | >CONTIGS_5501 | (GAA)5 | CCAGTTGAATCCTCACCTTGAT | TCAGAGCACCACTCACTCACTT | 222 |
| VMgSSR298 | >CONTIGS_5504 | (CTT)5 | GCCGCTACAGCCATCAAC | CAACTCCTTCCATCATCATCAA | 196 |
| VMgSSR299 | >CONTIGS_5551 | (ACGGCG)3 | TTCATCTTTACGACACTCCTCG | GTGTTCTACCTCGTCTTCTCCG | 343 |
| VMgSSR300 | >CONTIGS_5553 | (GTA)5 | GAGTTCAGTCAGAGGAGGAGGA | ACAAGCATTAACCCACACTTGA | 363 |
| VMgSSR301 | >CONTIGS_5605 | (AAATTG)3 | TGTCCTCAATCCATCGTTGTAA | AGTGTCCTCACCGTCTCAAAGT | 400 |
| VMgSSR302 | >CONTIGS_5616 | (CATC)4 | CACTTCTCAACCCACATTTTCA | AGTGAATAGCCTCCTTGTTGGA | 186 |
| VMgSSR303 | >CONTIGS_5624 | (GATATT)3 | CACCTTCTCTTCACATGGTTGA | GTCCGAACAAACCTACAGGAAG | 264 |
| VMgSSR304 | >CONTIGS_5633 | (CTCTCA)3 | AGAAGGAATCGGAGGTCTTGAT | GAGAAGAACAGAGGCATGAGGT | 265 |
| VMgSSR305 | >CONTIGS_5635 | (GAGGGC)3 | GTTATTCATGGAAGGAAGAGCG | CTTCGGAGAGAGTCAAAGTGGT | 356 |
| VMgSSR306 | >CONTIGS_5638 | (CCG)5 | TGGAGAGAGAGGATTTGGATGT | TTGAATTGAGTGGGAGTGAAGA | 179 |
| VMgSSR307 | >CONTIGS_5638 | (AGA)6 | CGTAAGAGATAAGACCGGCATC | TCTCCATAGCGTGTTCTTTGG | 231 |
| VMgSSR308 | >CONTIGS_5677 | (GA)6 | CCATGTGTTATTTCGTCTGCAT | AGCCTTTCCTCCTCCAATACTC | 183 |
| VMgSSR309 | >CONTIGS_5700 | (AAG)7 | TGGTGGAAAGATGAGTGTTTTG | CGGTTGTTTCTGCTCCTACTCT | 173 |
| VMgSSR310 | >CONTIGS_5752 | (TTTTTC)3 | CCACCCTCTCTAAAACACCAAC | AGACTTACACTCGGATACGGGA | 224 |
| VMgSSR311 | >CONTIGS_5766 | (AAG)8 | CAGAAAACATCATTGCCTCGTA | GTCTTGATAGCTGTTGCCCTCT | 180 |
| VMgSSR312 | >CONTIGS_5788 | (GCA)5 | CATTGTATTCCTGTTCAAGGCA | GAAAATGGGCAAGGTGGTATAA | 261 |
| VMgSSR313 | >CONTIGS_5806 | (GAGCG)4 | TTACAGAAATGGCAGGACAGAA | CGGATGATAGGGAATATGAGGA | 172 |
| VMgSSR314 | >CONTIGS_5817 | (GAG)5 | CACCATCATACCCTCATCTGAA | AGTTCCTCCTCCTCCATTTCTT | 274 |
| VMgSSR315 | >CONTIGS_5842 | (TC)8 | ATCCCCAACACAATACAACACA | GGATTCATAATTCACCTACTCCAAG | 247 |
| VMgSSR316 | >CONTIGS_5849 | (TGG)5 | CGAATCCTTCTTCATGCTTTCT | TGTAGTTTTGGTCATGGTTGGA | 228 |
| VMgSSR317 | >CONTIGS_5853 | (CAAGAA)3 | CAATATGGTCTCCTTTGCTTCC | CTATGGGGTCTCTCGTTGTCTT | 324 |
| VMgSSR318 | >CONTIGS_5930 | (CAT)5 | GCCATGCCTTGTTATCTCTTG | CATTGCTGTCATTCTTCTCACA | 280 |
| VMgSSR319 | >CONTIGS_5947 | (ATC)6 | TAATCAAACCACCAAACACAGC | TGCAAATCACGAGGATATTGAG | 116 |
| VMgSSR320 | >CONTIGS_6013 | (CGT)5 | CCAAGTCATTCTCTTTGTTCCC | TATGCTAAAAGTCGTCTGGGGT | 305 |
| VMgSSR321 | >CONTIGS_6014 | (CCACCG)3 | GAAGGTAGAGCCGAAGTTGTTG | TCCCTTATTCATTTGTTCCCAC | 208 |
| VMgSSR322 | >CONTIGS_6023 | (TCAC)5 | ATGTGATGGGTCTCTGTCTCCT | GGTTCCAAAGAAGAACTGATGG | 383 |
| VMgSSR323 | >CONTIGS_6041 | (GAA)5 | CTCTTTGGTACAGGGAAGAAGG | GCCAGGGATACTGATATTCTGC | 354 |
| VMgSSR324 | >CONTIGS_6072 | (CCACAA)3 | TCGGAGAAGCCATAGAATTGTT | CCGAGAACTTGCTATTTACGGA | 241 |
| VMgSSR325 | >CONTIGS_6097 | (GGAGGC)3 | GTGCTATCAGTGTGGCGATTT | CAATACCCTGTCATCTCCCATT | 345 |
| VMgSSR326 | >CONTIGS_6126 | (AG)8 | TCTGAACGAGATCAACCACTTC | ATTCCCTATCGCACTCACTCTC | 278 |
| VMgSSR327 | >CONTIGS_6154 | (GA)6 | TCCTTAGCTCCTCACTCTTTCG | AGGGGCATGTCAGTATCCTCTA | 294 |
| VMgSSR328 | >CONTIGS_6166 | (CT)9 | CCGGAAAAGGGAAAACTACATT | GCAGAACAGCAGAAACCTCTTT | 295 |
| VMgSSR329 | >CONTIGS_6184 | (GAA)6 | AGACAGCAACAAGAAAGAACCC | TTTTCAACTCTCCAAACCTTCC | 373 |
| VMgSSR330 | >CONTIGS_6187 | (TTCTCT)3 | GGACCATACAAAGATCCTCCAG | GCAGAGAAGAAGGAAAACCAGA | 319 |
| VMgSSR331 | >CONTIGS_6230 | (TC)6 | GAGAGCCAAGTATATCCAACGG | GTGTTGCTTAGTGGTGCAGTTC | 286 |
| VMgSSR332 | >CONTIGS_6234 | (TC)6 | GGCAGATAAACACATACACACATCT | AAGGGTTTTCGGATTAGCTTCT | 141 |
| VMgSSR333 | >CONTIGS_6272 | (TCT)6 | AGAGAGTGAAAAGAAAAGCCCA | AAACCTGTTGAGATCGAGAAGC | 221 |
| VMgSSR334 | >CONTIGS_6282 | (CT)10 | AGGTTTTCTTCACATTCTCTGG | TTTGAGGTCCAACTTACAGTCC | 379 |
| VMgSSR335 | >CONTIGS_6284 | (TGA)5 | GGATAAGCATTCAAAATCACGC | GCCATCATCTCCATGTGTCTTA | 364 |
| VMgSSR336 | >CONTIGS_6291 | (GAC)5 | ATTTTCCTCCTCTCACGCTTC | CTCCTTTTCCATTATCTCGTGG | 328 |
| VMgSSR337 | >CONTIGS_6297 | (TATGGT)3 | GAGCAGAATGAGAAGGTCCAAG | ATGAAGACGAAGACGAAGATGG | 234 |
| VMgSSR338 | >CONTIGS_6302 | (TTG)5 | GTGCATAAATAAGTGGTGCCAA | GGAAGCTGATGAGAGATCGTTT | 400 |
| VMgSSR339 | >CONTIGS_6316 | (CGC)5 | GTTTGAGTAATTTGCGAGGGAC | GCAAGTAGAAGTGAATGTCCTTGTT | 207 |
| VMgSSR340 | >CONTIGS_6320 | (GTG)5 | TAGCCACACTATTCCCCTTGTT | CACTCATTCACACGATTGTCCT | 238 |
| VMgSSR341 | >CONTIGS_6338 | (GGA)5 | TCTTCAGTTTCTGGTTCTCGCT | CCAAGGATTTGCTTCCTAAATG | 369 |
| VMgSSR342 | >CONTIGS_6352 | (ATC)6 | GAACCTGAGACTAATCGAACCG | GATGAAGACAATGGTGAGCAGA | 283 |
| VMgSSR343 | >CONTIGS_6354 | (GA)7 | AGGGTTTTCGGGAGAGAAATAG | TCCTCTGTAGCCCTCTTCAAAT | 349 |
| VMgSSR344 | >CONTIGS_6358 | (AT)14 | TTCTCATCCAGGTACTGCTGAA | CAGAAACCCAGAAACAAACACA | 309 |
| VMgSSR345 | >CONTIGS_6392 | (TC)10 | TCTGGTGATTTTGAGTTAGCGA | GAGATCCAATTTCTGGTTGTCC | 277 |
| VMgSSR346 | >CONTIGS_6399 | (CAT)7 | GTGTTTGGGGAAATAAAAGGGT | GCGGAAGGAGAAAGAGGACTTA | 270 |
| VMgSSR347 | >CONTIGS_6466 | (ACC)5 | CCAGACCCACACCCAGAA | GTGAGATTTACAGCGAATAGAGCA | 160 |
| VMgSSR348 | >CONTIGS_6492 | (GAT)5 | GTTTCATCTTCTTCCCCATTCA | CATACTCTTCCTCACCCTGTGC | 237 |
| VMgSSR349 | >CONTIGS_6507 | (AGC)5 | CCTAGCATTGTGATCTGTGGAA | GCCAAGAAGAAGGAAGCACTTA | 273 |
| VMgSSR350 | >CONTIGS_6512 | (GT)7 | GATACAAGACTCGCTGCAACC | TCTTCCTCTGCTTTGCCTTC | 159 |
| VMgSSR351 | >CONTIGS_6529 | (TTGCTC)4 | TGGCTATTGGAGAGGATTCATT | TCGATTTGCTTCCTAAACCAGT | 304 |
| VMgSSR352 | >CONTIGS_6604 | (TCA)5 | TGCAAGTATGTTTCCTGTTGCT | AACAATCAAGGTATCGGCTGTT | 238 |
| VMgSSR353 | >CONTIGS_6611 | (AGGAAG)3 | AACCCTGCAACAAGAAATCACT | GGTGTCTCATCACCTTCTCCTT | 212 |
| VMgSSR354 | >CONTIGS_6621 | (CGG)5 | TACAATGCAAAATAGCACCCTG | TTCTAAAAGGTACGCCGAAAAG | 314 |
| VMgSSR355 | >CONTIGS_6759 | (GGAGGC)3 | CACCCTCTCAACCTTGGACTAC | CCGAAAGATCCTCAAAGAAAAC | 361 |
| VMgSSR356 | >CONTIGS_6775 | (TA)7 | TGCTTCTGTGGCAAATTCTCTA | CAGTTAAGTTCATGGCAACCAG | 350 |
| VMgSSR357 | >CONTIGS_6796 | (CT)7 | GATTGTTGAGAGTGCAGATGGA | TGTTGAAGAAACAGTGGGAATG | 257 |
| VMgSSR358 | >CONTIGS_6820 | (TGC)5 | AAACCTGAAGGAGTTTGTTCCA | GTGATTGCATGTGGAGAAGAAG | 305 |
| VMgSSR359 | >CONTIGS_6830 | (TAG)6 | ACCAATTCTCCACCACCATAAC | ATGCTACGACTACGGCTACTCC | 259 |
| VMgSSR360 | >CONTIGS_6872 | (CTC)5 | TGACAGGGAGTATGGAGGCGTT | AGAGCGCCGGGAATGGAG | 337 |
| VMgSSR361 | >CONTIGS_6881 | (TC)10 | GCAGTTTGCTTGTCTTTCCTTT | TGATGCTCTCACTGGTATAGCG | 242 |
| VMgSSR362 | >CONTIGS_6895 | (CT)12 | ATCCTTCCTTCTTCCTCTGCTT | GCTTTGGGGTGTTATCTTCTTG | 162 |
| VMgSSR363 | >CONTIGS_6932 | (ATTTCA)3 | TTTTATCCCCTACACATACGCC | AATTGTCTCTCCAGAAGCATCC | 388 |
| VMgSSR364 | >CONTIGS_6968 | (AAGTGA)3 | AGGAAGGAAGTGAGGAGCTACA | TCTGATAATTCAAACGACACCG | 157 |
| VMgSSR365 | >CONTIGS_6977 | (GAA)6 | ACCTTCTTATTCATCACACCCG | ACTCGCTTCAAAACTCGTTCAT | 107 |
| VMgSSR366 | >CONTIGS_7034 | (AGCT)4 | TATCCCAGAGCAGCACAACTTA | AGTAGGTTTTACACGCAGGAGC | 170 |
| VMgSSR367 | >CONTIGS_7043 | (GAG)6 | CTGTGGTGGAAGTAGCATACCA | GAAGAACGCAAGAAAGAGGAGA | 360 |
| VMgSSR368 | >CONTIGS_7069 | (GAT)9 | AAGACGAGGTTTCTGGTTCAAA | CAGCAGCAGTCATAAGTTCCTG | 349 |
| VMgSSR369 | >CONTIGS_7119 | (GTGGCG)3 | TTTAATGAATCGAGGTTGACGG | TACCAAAAGATGGGCTGACG | 102 |
| VMgSSR370 | >CONTIGS_7132 | (GCTGCA)3 | AACAACAACTGCTGCTTCACAT | GGGGATTAAGTTCACAGGTTCA | 210 |
| VMgSSR371 | >CONTIGS_7136 | (AG)9 | ATTTGACAGTTGGGAAACCCT | TCTCACACTTCTTCTCTGCACC | 216 |
| VMgSSR372 | >CONTIGS_7156 | (AG)8 | AGTCATCGTAGGAGCAGAGGAT | AAGTAACGACGCAGCATAGGAT | 233 |
| VMgSSR373 | >CONTIGS_7187 | (CTC)6 | TGAGAGTGTTGTTGCATGTGAG | GGTTTTAAGATCCGGTGAGGA | 204 |
| VMgSSR374 | >CONTIGS_7241 | (TGC)5 | ACCTTGTGTACGCCTTGAAAAC | CACTGGACGGAGACCTAAGAAG | 216 |
| VMgSSR375 | >CONTIGS_7264 | (ATC)5 | TATTCTTTCCATGCCCTTCACT | GATACAGTGCCTTGGTTGAGGT | 385 |
| VMgSSR376 | >CONTIGS_7281 | (TGTT)4 | CAGTGAGAGGGTGGTAGAAAGG | GACGGTGGATTTTGTGTGTATG | 206 |
| VMgSSR377 | >CONTIGS_7391 | (TCA)5 | CATAGGAGAAGCCATGTTAGGG | TGAGGAACCAGAAAATCAACCT | 273 |
| VMgSSR378 | >CONTIGS_7402 | (GA)7 | TGGTACAATCACCATGTCAACC | CACCCTTCTCACTCTCTTTCTCTC | 364 |
| VMgSSR379 | >CONTIGS_7430 | (GAA)5 | AACGTGTGCTCTTCCGATCT | CTAGTGGGAGACAGAATCAGGG | 294 |
| VMgSSR380 | >CONTIGS_7454 | (TCA)5 | CAACTTCTTCCTCTTCGATGCT | ATGCTACCGAGGCTAACTGAAG | 257 |
| VMgSSR381 | >CONTIGS_7459 | (GCA)6 | GATAGTAGTTTACAAGCGCGGAA | AGTTGTGGTTGGAAGGATTAGC | 356 |
| VMgSSR382 | >CONTIGS_7478 | (TGCTGG)3 | AGTTCAGTGGAGAAGGAGGTTG | AGTTCCAACGACAGAGGTTCTT | 116 |
| VMgSSR383 | >CONTIGS_7548 | (ATC)5 | AAAACAGAGGCACAAGAGAAGG | TATGATGGAGGAGGTAGCGACT | 218 |
| VMgSSR384 | >CONTIGS_7573 | (GCAACA)3 | GCAGAAGATACAACGAAGCAGA | AGAGAGAGAGAGAGAGGGGAGG | 114 |
| VMgSSR385 | >CONTIGS_7613 | (AT)6 | TTAAGATAGTTTGGGGCACGTA | ATGTTGGTGGCTAAAGACAAGT | 138 |
| VMgSSR386 | >CONTIGS_7643 | (CT)11 | CAAAGATCAGTGTTTCCCACAA | AACCTTGTCGTGTTCAATCCTT | 204 |
| VMgSSR387 | >CONTIGS_7693 | (GCT)8 | AGAACAAGGAGTTGTGGTGGTT | GCTTCGGATGACAAAAGTTACC | 393 |
| VMgSSR388 | >CONTIGS_7755 | (GAA)5 | AAGTGAGGTTGGTTATGCCTGT | CTTGGCCTATGTCTCCTTCAAA | 162 |
| VMgSSR389 | >CONTIGS_7813 | (CAG)6 | TCCTTCTTTAATTCCAGGCTCA | TGTTGCAGTAAACCATTTCCAC | 132 |
| VMgSSR390 | >CONTIGS_7816 | (ATT)6 | TTACCACTTCAGGAGGACCATT | TGGAGAAATGACTTTGGAGGAT | 353 |
| VMgSSR391 | >CONTIGS_7843 | (GAAATG)3 | GCATTCCCTTTCTTTTGTTGTC | CTCTGCCCTTCAATTACCCAT | 391 |
| VMgSSR392 | >CONTIGS_7903 | (ACT)7 | AGGGACAGGATGACAGAAGAGA | CAACGGAAAGAAAATCGTTAGG | 308 |
| VMgSSR393 | >CONTIGS_7928 | (CGC)5 | AAACATGAACCTCTCCACCCTC | GTTGCTGATGCTGGCCCT | 269 |
| VMgSSR394 | >CONTIGS_7936 | (TCC)5 | CTTCATTTTCACCGCATTACAC | GTACACTGGAGAGGGAATGGAC | 190 |
| VMgSSR395 | >CONTIGS_7975 | (CA)8 | CTCTGAATTGGCTTCTGGTTTC | TTTTGTTATGGGTCGGTGTAGG | 277 |
| VMgSSR396 | >CONTIGS_7985 | (GAA)5 | AGAGAGAGAAGCAGAGCCTGAA | AAAGGGGTAGCAAACATCAGAA | 347 |
| VMgSSR397 | >CONTIGS_8056 | (ATC)5 | AATCACAATCATCAGGACCCTT | AGCAATGAAATTACCCATCACC | 281 |
| VMgSSR398 | >CONTIGS_8111 | (ACCATC)3 | TATCTAATCCTGTTTGCTCCCC | CGTTCTGATGTTGCTGATGATT | 278 |
| VMgSSR399 | >CONTIGS_8123 | (GCG)5 | AAGCACCAGAAAGTGTGTTTCC | ACTACAATTCCCCTGAATCCAA | 279 |
| VMgSSR400 | >CONTIGS_8142 | (TACTTT)3 | CGAACCTATCAACCTCCTCATC | GAGAGACATGGAGAAATCTGGG | 206 |
| VMgSSR401 | >CONTIGS_8236 | (GGC)5 | GACATCTCAACAACAGGAGCAG | AGGACAACAGAAATCGAACGAG | 264 |
| VMgSSR402 | >CONTIGS_8313 | (TC)9 | CCCACTTAACCACCCTATCGT | AACCTTGCTATCTGGATCTTGC | 201 |
| VMgSSR403 | >CONTIGS_8323 | (GTA)5 | AGCTGAACCTGGACTGAGATTT | AGGCTTTAGTTTTCCCCTTACA | 217 |
| VMgSSR404 | >CONTIGS_8336 | (TTCTGC)3 | CTACTGGCTTGCTACCACACAG | TCAGCCTTTCCTCTTCTTCTTG | 369 |
| VMgSSR405 | >CONTIGS_8338 | (ATA)6 | AGTGCTTAATACCTGTGGTGCC | CTTCATGGTGGTGCTGTAGAAA | 312 |
| VMgSSR406 | >CONTIGS_8406 | (AAG)5 | AGTAAGTGCGTTTTATCCACGC | GAACCACAACCACAACTCCAA | 101 |
| VMgSSR407 | >CONTIGS_8410 | (CGGCGA)3 | CTCTCTTCTCCTCCCGCAC | CGTCTCTACCTCCTTGGAATCA | 168 |
| VMgSSR408 | >CONTIGS_8414 | (AT)7 | CGGCCCTAATGAATTGTAATC | GTGGTAGCTCTCAAAACAGTGC | 315 |
| VMgSSR409 | >CONTIGS_8514 | (CTT)5 | ATTACTCTCTTTCCCGGTAGCC | GCTGTTCAAGTTCCTAACGACA | 129 |
| VMgSSR410 | >CONTIGS_8548 | (TAC)5 | CGTAATTTCCATAACTCGCTCC | AAATCAAGAACGATCCAAGTCC | 349 |
| VMgSSR411 | >CONTIGS_8572 | (GAT)6 | AGTGATGATGATATTGTTGGCG | TTTCTCTCCTAGTACGGCTTGG | 232 |
| VMgSSR412 | >CONTIGS_8688 | (GAT)5 | TCATCATTTCACAAAGGCTCC | GTGGTGCAGGAGTACGAAAAT | 360 |
| VMgSSR413 | >CONTIGS_8742 | (TTC)5 | GATAGAAAATCTGCCACCCAAG | GAAAATGGTGAGAATCCGAAAG | 164 |
| VMgSSR414 | >CONTIGS_8796 | (ACC)5 | CTGATGCTTTTCCAACAGAGTG | TGTTCATCTTCCAACACAGGAG | 373 |
| VMgSSR415 | >CONTIGS_8827 | (CTT)5 | CCATTGTCTTGACTGGTAGCTG | GAGAAAGAGGAAGGTAGAGGGC | 204 |
| VMgSSR416 | >CONTIGS_8858 | (GGAATG)3 | GATAAAATCATCAGCAGCAGCA | AGAGCCCATACTAATCCATCCA | 156 |
| VMgSSR417 | >CONTIGS_8874 | (TGA)6 | CACAACAAGGTGAAATCTCTCG | GAAGTTGGTGCTGATGGTTTCT | 365 |
| VMgSSR418 | >CONTIGS_8879 | (AGTGGC)3 | ATAACTCTTGCCCCGTTTGTC | TGACCACGCAAAATAACATCTC | 387 |
| VMgSSR419 | >CONTIGS_8915 | (ATC)5 | TACAACTCCATTTTCACGTTGC | GAGGCTAATGAGGGAACAACAC | 254 |
| VMgSSR420 | >CONTIGS_8937 | (AGGC)4 | GTGATCTACTCAGCTTCAGCCC | GTTCTCCTGCTTTATCAGTGCC | 131 |
| VMgSSR421 | >CONTIGS_8996 | (TCA)7 | AAATGTGTCTCCCAAAGTCTGC | AGGATTGCTGATTGCCTAGTGT | 122 |
| VMgSSR422 | >CONTIGS_9018 | (AG)6 | AGGTGGAATGTGGGCTACTATG | TGGCTTTGTCTGGTAAACTCAC | 398 |
| VMgSSR423 | >CONTIGS_9039 | (CCT)5 | CACCAGCTCCACCTCCAAT | AAGAATGTAGGGTTTGGGAAGG | 229 |
| VMgSSR424 | >CONTIGS_9078 | (TC)6 | CAAGTTAAAATGCCCACCGTAT | ACCCAAGTAATTCTGCGTTTGT | 360 |
| VMgSSR425 | >CONTIGS_9112 | (AG)6 | TGGCTACCATATCTTCATTACACC | GCAGGCTTAGATTTTGACACCT | 339 |
| VMgSSR426 | >CONTIGS_9142 | (CCATCA)3 | AGAGACATAAAGCCTGCCAATC | AGCATCAACAAAGGAGCAGAAT | 339 |
| VMgSSR427 | >CONTIGS_9173 | (TTCC)4 | CAATGAACACAAGGACGACAAG | ACAGGATAGGAGAGGACACCCT | 102 |
| VMgSSR428 | >CONTIGS_9191 | (CCGACA)3 | CTCAATTTCCTTCTTCAAACCG | ATCTTCGCTCTCTCTCCACATC | 292 |
| VMgSSR429 | >CONTIGS_9194 | (TTC)6 | ATCAAAACAACCCTGCTCTTGT | TGAGTAGACGGTGGGAAGAAAT | 169 |
| VMgSSR430 | >CONTIGS_9218 | (TC)16 | TGTAAGAGCAGAAAAGGCATCA | GAACTGGACAACACAGGTTTCA | 249 |
| VMgSSR431 | >CONTIGS_9236 | (CA)9 | AGAAAGATTCGCAGTGACAACA | ATCTAATCGGTGTTTGGTTGGT | 388 |
| VMgSSR432 | >CONTIGS_9299 | (GTA)7 | TGAGGAGTAACAGTGCCTGAGA | GGAAATCCGTGCTAGAGAGCTA | 223 |
| VMgSSR433 | >CONTIGS_9302 | (GGCTT)4 | AGCTGGGGACACTTTTGACTAC | GATTTTGTAAACCATCGTGGG | 342 |
| VMgSSR434 | >CONTIGS_9329 | (CAT)5 | CCCTGCATACTTGAATAGTTGTCTTC | GGGAGTGGTGATCTCTGCAA | 215 |
| VMgSSR435 | >CONTIGS_9344 | (GAA)6 | CCGAGATTAAAGTGGGACAAAA | ATTACATACCCGCGCTTGAG | 227 |
| VMgSSR436 | >CONTIGS_9376 | (GCCTTC)3 | TTTCGCAATCGTTGTAGTTGTC | GCAGAAAACCGCTCTTACTATTCT | 301 |
| VMgSSR437 | >CONTIGS_9422 | (GCA)5 | GGGGTGAACTCAAAGTCAAAAC | CAGATAACGGGAAGCGAACTAC | 124 |
| VMgSSR438 | >CONTIGS_9458 | (GA)17 | ATGGAAATTGTGTGTGTGTGTG | GCTCAATAGATACCGCTGACCT | 188 |
| VMgSSR439 | >CONTIGS_9490 | (GAAAAA)3 | CAGACTAACCACAGACCCACAA | ATCTACTGAAGCGGGAAGACAA | 319 |
| VMgSSR440 | >CONTIGS_9494 | (CAAGAA)3 | AAATGGGAAGTGTATGGGTCTG | AAGTTCCTTGGTGGAGGGAAT | 263 |
| VMgSSR441 | >CONTIGS_9528 | (CTT)7 | GGTTGGAGCATAGAAGAAATCG | AAGAAAGAGAGCGTCGTTGAAG | 282 |
| VMgSSR442 | >CONTIGS_9531 | (GAT)5 | GCTGAAGAGGAGGAAGCATCTA | AGACTATGCACCAAGCATTTGA | 368 |
| VMgSSR443 | >CONTIGS_9607 | (TTCTCT)3 | TCAGCATTCTCTCGTTGCC | TTGATTTCCTTGATGAGCTGTG | 358 |
| VMgSSR444 | >CONTIGS_9675 | (AT)6 | GTAAAGATCAGGCACACAAACA | CACCCTGCAAATTCCTGTA | 399 |
| VMgSSR445 | >CONTIGS_9701 | (ATC)6 | ATCAATCTTGTATGCCCAATCC | CAGTTTCTGATGGTTATGCGAA | 311 |
| VMgSSR446 | >CONTIGS_9706 | (GGC)5 | CGACAGGAGTATGGCAGTGTAG | AAGATTTGTATCACATGACCCG | 179 |
| VMgSSR447 | >CONTIGS_9776 | (GATGAA)3 | AGACTCAAGTGCTCCCAATCTT | CTCCTTCATGCCTTCATTTTCT | 241 |
| VMgSSR448 | >CONTIGS_9782 | (TC)7 | AGTGTTGCTTCTTTGCCCTC | TACAACCTGTGATGAGGAATGG | 215 |
| VMgSSR449 | >CONTIGS_9842 | (ACC)6 | GGAGGAATACCAGCATCTTTTG | CTCGAAGAGTTTTGCAGTGTTG | 143 |
| VMgSSR450 | >CONTIGS_9854 | (ACC)5 | AAGCAGATGGAGGAGAGTGAAG | AGAGCAGAGAAGGAGCAACATT | 400 |
| VMgSSR451 | >CONTIGS_9896 | (ATTGAA)3 | CTATCTGGGTGGCCTGGTATT | GTGACCCTTACCTGAAGAGCTG | 217 |
| VMgSSR452 | >CONTIGS_10072 | (CAA)5 | ATGAGACCTTCATCTGTGGGAT | CTCACTGGCTGAACTACACTGG | 392 |
| VMgSSR453 | >CONTIGS_10091 | (CT)10 | TACACTACTCTTTCCCTCGCCA | GCCATTTATAGCCACCAACAAC | 333 |
| VMgSSR454 | >CONTIGS_10111 | (CAGCAA)3 | GAAAACCAACAACCATCACTCA | CACACTTCCGTCTATGCTCAAG | 388 |
| VMgSSR455 | >CONTIGS_10112 | (GAA)5 | GCTACAGAGCAGCATCAGAGAG | AGACAGACTTTGCATCAGTGGA | 264 |
| VMgSSR456 | >CONTIGS_10138 | (ATGAAG)3 | ACAAGGTAATATGGGCAGTTGG | CGGCCTTTTCAATTTCTACATC | 171 |
| VMgSSR457 | >CONTIGS_10143 | (ACC)5 | CTGTAAGGCACGCTCACAAA | GATAAGGGTTCGACAGGGACT | 245 |
| VMgSSR458 | >CONTIGS_10178 | (AG)6 | GAAACGAGATCAAACAAGGGAA | GGAATCGACAAGCTCTATCACC | 312 |
| VMgSSR459 | >CONTIGS_10203 | (GA)6 | CATTCAGTCGAAACTTGGTCAG | AAAACTTGGAGAGGGAGAAAGG | 315 |
| VMgSSR460 | >CONTIGS_10235 | (TA)15 | CACTTCATCACATTCTCCTCCA | GAATTTACTGGCTTCGTTGTCC | 384 |
| VMgSSR461 | >CONTIGS_10241 | (TGC)5 | ACCTCCTTCCCAAGCTATTGTT | AGCATTCTGTCAACCCTCTGAT | 154 |
| VMgSSR462 | >CONTIGS_10259 | (TA)8 | TCCAAAAGGGTCGAAAGTGTAT | CTGAGATAAGTTCCCACTAAAGAGGT | 239 |
| VMgSSR463 | >CONTIGS_10268 | (TTG)5 | AGGGTCCAACGTAGTTCTCAAA | TAGCTCTTCAACATGACAGGGA | 346 |
| VMgSSR464 | >CONTIGS_10293 | (TC)9 | TGAACTTGATTGCTTCCAGATG | GTTTTCCATTGTTAGCGAGTGC | 253 |
| VMgSSR465 | >CONTIGS_10343 | (CAC)5 | GTGGAGGCTAAGTATTATGCGG | GGAAACCAAGCCAGTAAAGAAA | 330 |
| VMgSSR466 | >CONTIGS_10387 | (CAC)5 | TTGGGAGATGCTATTGCTTACA | CAGCTCACCTTTACTACCACCC | 197 |
| VMgSSR467 | >CONTIGS_10420 | (TTCATC)3 | CATGTGGATCTCCTGGGAA | TGGTGAGGATGTTATGTTGAGC | 115 |
| VMgSSR468 | >CONTIGS_10451 | (TTG)6 | AGTGCTGGGATTACAGGTGTG | GAGGTGGAGGTTGCAGTGAG | 154 |
| VMgSSR469 | >CONTIGS_10452 | (TCT)11 | TGGGCTTTTGTGGAAGAGTAAT | AGAGACCAACGAGATGACCAGT | 194 |
| VMgSSR470 | >CONTIGS_10485 | (GAA)5 | GGAGTAAGCCATGTGAGAAAGC | AAACTGGAAGCAACGATGGTAT | 294 |
| VMgSSR471 | >CONTIGS_10520 | (CAT)6 | TCTGTTATCTGCAACTTCCGGT | CTTCGAGGTCTACGAGAACGAC | 375 |
| VMgSSR472 | >CONTIGS_10601 | (CAT)6 | AATCCTCCCACACTGGTATTAGA | CTGATGCAAGAAATTGACACAC | 233 |
| VMgSSR473 | >CONTIGS_10625 | (TA)7 | CACAGCAATTCTCAATCGGTTA | CCCATCTACTATCTTGTGCATCTG | 189 |
| VMgSSR474 | >CONTIGS_10630 | (CTTTCT)3 | TTCGTTCCTCAGCTTCTTCAAC | CTCCTCCTCCCGCATTTG | 284 |
| VMgSSR475 | >CONTIGS_10679 | (TC)7 | ATCTTTGAACTGAAGAAGGCCA | TCTGAAGCCGTAGTTGAGACTG | 239 |
| VMgSSR476 | >CONTIGS_10790 | (CT)6 | ACCGCCACAGAAATTAGAAATG | AAGAGTGGAAGGAGGAAGAAGG | 118 |
| VMgSSR477 | >CONTIGS_10811 | (ACAT)4 | GGAACCTGAAAACCAAAGGTAA | GGCAGTGTAGGAGAAGAATGGT | 143 |
| VMgSSR478 | >CONTIGS_10946 | (GTT)5 | AATAACGGGAATGGAGATTGTG | AGAGCAGTTCCACCTCTTCAAC | 365 |
| VMgSSR479 | >CONTIGS_10955 | (CT)6 | TCCCCTCTCTTCTCTAACTCCC | TAGATAGCTCCACAACATCCCA | 282 |
| VMgSSR480 | >CONTIGS_10991 | (GGT)8 | TAGATCCGTGTCAGAGGTTGTT | CAAGACAAATGACCCACCTAGT | 254 |
| VMgSSR481 | >CONTIGS_10993 | (TC)10 | GCGCATGAGAGTTAGTGATTGA | TGGGATTTTAGAGAGAAGGCAG | 165 |
| VMgSSR482 | >CONTIGS_10995 | (TGA)5 | AGCAGAGCAGCATCAAGAGTAA | ATGAAACCTTGCCATCTGACTT | 289 |
| VMgSSR483 | >CONTIGS_11017 | (GGTGAC)3 | ACAGTTGCAGATGAAAACCTCA | CACCACCACCAGTATCAGTGTC | 393 |
| VMgSSR484 | >CONTIGS_11072 | (GAAGAC)3 | GAAGACCGTACATCAAAGCCA | AGAAGATTCCAGCAAGAAATGC | 289 |
| VMgSSR485 | >CONTIGS_11160 | (CCAAAA)4 | TCACCGCAAAATACTCAACTCA | CACACCATCGTATCCTAGACCA | 222 |
| VMgSSR486 | >CONTIGS_11209 | (AAAAAG)3 | AATCCTAAAGCCGAAGCAAGTA | TCCTTTGGTGTTCAAATCCTCT | 228 |
| VMgSSR487 | >CONTIGS_11248 | (GAA)6 | AACTAGCAGAGGACCAAACTGC | CTTCTTAACTCTCGCCTGGAAA | 278 |
| VMgSSR488 | >CONTIGS_11252 | (TC)10 | ATGGATGCACAACTCAAAACAC | GCAGATAGAAAAGCAGGGAGAA | 166 |
| VMgSSR489 | >CONTIGS_11261 | (CTT)6 | CTCTACAAACCCTAACCCGTTG | GAGGAAGAAGACGAGAAGGTCA | 102 |
| VMgSSR490 | >CONTIGS_11312 | (TTC)5 | ATTCCTTCCCTTCAAACTTCCT | AGTAACAGTGGATGGTTCGTGA | 244 |
| VMgSSR491 | >CONTIGS_11313 | (GTT)6 | GAGGGGAAGAGAGTGAAAAGGT | GATAAGGGAAAGGGAATTGGTC | 227 |
| VMgSSR492 | >CONTIGS_11499 | (TCTTCA)3 | CACTTTTATCCCAAACCACCAT | CCCTGAATCCACAGTACAATGA | 339 |
| VMgSSR493 | >CONTIGS_11507 | (CTCAAC)3 | TCATCCCCAGGACTTGTTTTAC | GGAATCTCCCAGTGTTTACGTG | 389 |
| VMgSSR494 | >CONTIGS_11556 | (TG)7 | TAAAAGACGGCTGTGTCGTAAG | CTCTGTACTGCAAAACCCCAAT | 349 |
| VMgSSR495 | >CONTIGS_11593 | (GCA)5 | ACAGAGCCAACACCTGAATCTT | TTAGGCGCTACAGCTAATCACC | 344 |
| VMgSSR496 | >CONTIGS_11681 | (GCA)5 | ATGGAGATCAACAGACAGACCA | TTGGAAGAAACTGAAGGAGGAG | 180 |
| VMgSSR497 | >CONTIGS_11768 | (GAA)6 | AGAGCTTTTGACGAAGAAGTGG | AGACTGAAGATGGCTACCGTGT | 178 |
| VMgSSR498 | >CONTIGS_11777 | (AAG)5 | CGATTGTTCTAAGCCCATTGTT | CGGGTAGCCTTATTTTCTGTTG | 219 |
| VMgSSR499 | >CONTIGS_11801 | (ACCCAA)3 | CTTACAAAGCCCTCAAAAGCAC | CTGTTGTTGCTCCTTCTCTCCT | 224 |
| VMgSSR500 | >CONTIGS_11846 | (TCT)5 | CTGATCCCTTTCTCTTTCCCTT | AAGCAATGATGGTAACGTCTCA | 385 |
| VMgSSR501 | >CONTIGS_11850 | (TTTGAT)3 | CTATTTCTTACTGCCCCGTGTT | CCCAGTCTAAACCTTCACATCA | 250 |
| VMgSSR502 | >CONTIGS_11903 | (CAT)5 | GGTCTCTCACATGCTCTATGGC | GAGTGGTTATTTGCACCATTGC | 355 |
| VMgSSR503 | >CONTIGS_11920 | (TCA)5 | CAGTGAAGAGAGCTACGCTGAA | CAGTGGTGGAGTATGTGAGGAA | 246 |
| VMgSSR504 | >CONTIGS_11960 | (CGCCGG)3 | GGAGATCCAGTTCCTCAAATCA | TATTCACACTCAGAAGCAGGCA | 309 |
| VMgSSR505 | >CONTIGS_12034 | (CGT)6 | TGGATGCCTTCTCTTAGCAAAT | CTCTCTCCGATTCTTACCGCT | 363 |
| VMgSSR506 | >CONTIGS_12173 | (CGA)6 | TGAAGAGGAAGAAGAAGAACTGG | ACCACCTACTTTTGACCCTGAA | 115 |
| VMgSSR507 | >CONTIGS_12285 | (CAGCCA)3 | CTCTGCATAAAGTGATGGAACG | CCGTGTAAAAGCTCTGGAATCT | 262 |
| VMgSSR508 | >CONTIGS_12373 | (GA)9 | CCTGGGTGAGAGAGAGAGAGAG | AAGGCCAGAGAGTGTAATCGAG | 341 |
| VMgSSR509 | >CONTIGS_12374 | (TTTCTT)3 | AGTATTTCCGTCATTCGTTGCT | AATCCTTACGTTATCGCACCAG | 323 |
| VMgSSR510 | >CONTIGS_12397 | (GAG)7 | GGAAATAGATTCGCCAAAGATG | ATGAAGATCCAACCCATACCTG | 390 |
| VMgSSR511 | >CONTIGS_12403 | (GT)18 | ACCCAGCAACTACGAACACAA | TGTCAGGAGATACAGAGGCAGA | 148 |
| VMgSSR512 | >CONTIGS_12405 | (CT)6 | TTCATACATCACGCATTCCTTC | CTGAATCCAAATCCCTCTTTTG | 258 |
| VMgSSR513 | >CONTIGS_12448 | (GTTTTT)3 | AGAGGCAAATGGCAATAGAAAG | TAAACTCGCCAACCTGTGAGTA | 381 |
| VMgSSR514 | >CONTIGS_12497 | (GA)6 | GTCTGTCCCTACTTGGCTTCTG | GAGGTGCCTGAGATATTGATCC | 223 |
| VMgSSR515 | >CONTIGS_12625 | (GAG)8 | AATCCCTCTTGAAACCCAATTC | TTGATGTTCCTGTGAAGAATGC | 343 |
| VMgSSR516 | >CONTIGS_12779 | (AATTTT)3 | AATAGAGGAGATGCTGACGTGG | CATTACACGAACAAACCTACATGC | 336 |
| VMgSSR517 | >CONTIGS_12856 | (CTTGGA)4 | AAGCAACACACGACAAGACAAC | GAAGGAAAGACAGAGGAGCAAA | 349 |
| VMgSSR518 | >CONTIGS_12929 | (TGA)5 | AAGCTGGAGAAAACAAACAAGC | GCCAAGATCAAGGAAACATACC | 309 |
| VMgSSR519 | >CONTIGS_13025 | (ATAG)4 | CATCTTCAGGAGGTATCCCAAG | AGACCATTCCGATTTTCATCAC | 186 |
| VMgSSR520 | >CONTIGS_13036 | (GAT)5 | TAGGGCAAAATCAGGAGAGAAA | ACAACCTCCCTTAGAAACCGAT | 387 |
| VMgSSR521 | >CONTIGS_13076 | (CA)6 | GCAGGTACTGGTGTAGAATGATATG | GTTCCCGATGTGTGTTTCTGTA | 159 |
| VMgSSR522 | >CONTIGS_13141 | (GTTGAG)3 | ACCCAGAGTGAGAATTGTAGGC | CACCAGTGCTTTCATGTACGTT | 316 |
| VMgSSR523 | >CONTIGS_13179 | (CTT)5 | CCCCAAAACGTAAGAACGATAG | CATTCCTCTCCGTGTGTATGAA | 181 |
| VMgSSR524 | >CONTIGS_13185 | (CAAAAA)3 | AACAGGATAAATGTCGTCCACC | GCAAATGGAATGGGATATGAGT | 360 |
| VMgSSR525 | >CONTIGS_13255 | (CAT)5 | CATCAAGATTAACCGACCCATT | CTGGTGATTTGGAAGTGAGGAT | 165 |
| VMgSSR526 | >CONTIGS_13261 | (CAG)5 | GAGTTTCTTGGGTTTTGAATCG | AGCTGGTGGAATCTTGAGTCTT | 348 |
| VMgSSR527 | >CONTIGS_13330 | (GA)7 | TCGTATCTGTGTCCTTCTGGTG | GTTCTTCACACTCTTCTGTGCG | 140 |
| VMgSSR528 | >CONTIGS_13438 | (AGG)5 | GCAATGGTGATTCCTCTCTTCT | TTTTGCAGTCTCATTCTCCTCA | 383 |
| VMgSSR529 | >CONTIGS_13485 | (GAA)5 | AGGTTATTGGAATGGAAGAGCA | GGTGGTGGAGAGAGAAGAGAGA | 227 |
| VMgSSR530 | >CONTIGS_13498 | (TC)8 | TAGTTGGTTGTGGTGACGGTAG | GCCAAATGGTAGTGCAGTGATA | 103 |
| VMgSSR531 | >CONTIGS_13514 | (TCCACC)4 | ACAGTCCCACTATCAGGACCAT | TAAGTTCTTCACCTGGCATCAA | 163 |
| VMgSSR532 | >CONTIGS_13548 | (CAG)5 | GAGGGAAGATGACAAATGTTGG | GCTAAAATGTGCAGAACTGTCG | 355 |
| VMgSSR533 | >CONTIGS_13558 | (CCA)5 | TGCAAGGAAACTACCACAGAAA | CTACTTGGTTTGGGTTCCACTC | 367 |
| VMgSSR534 | >CONTIGS_13571 | (GAT)5 | ACTGAAGAGTGCAATGACAAGG | TATCTTTGATCCTATGGTGGGG | 190 |
| VMgSSR535 | >CONTIGS_13601 | (ATAA)4 | ACAGTCAATCCAACACTCCCTG | GTTTTGTGCGCTTGGGGT | 168 |
| VMgSSR536 | >CONTIGS_13742 | (AGA)5 | TGAAGAAGTGTAGGGAAGCGAT | AGTGTATGAGCTAAAGCCTGCC | 215 |
| VMgSSR537 | >CONTIGS_13845 | (AGAATC)3 | GATTCCGATTATTCCTTTTCCC | GAAGGGAGTTTGCTTATCATGG | 321 |
| VMgSSR538 | >CONTIGS_13864 | (GA)6 | GTCTCGTCCTTTGACCTTGTCT | AGTTATCTTCTCCGTCCAAACG | 170 |
| VMgSSR539 | >CONTIGS_13888 | (CAT)5 | ACCATCTTGTATCCCCTCCTCT | ATCCTTCATATCTCCCAAGCAA | 260 |
| VMgSSR540 | >CONTIGS_13914 | (GCCATT)3 | TTCCAGTGAAGGGACAAACTCT | GAAGAGATAACAAACGCAGAAGC | 244 |
| VMgSSR541 | >CONTIGS_13919 | (TC)6 | AGGTTTTCTTGCATCTGTTGGT | TCCCCTTAGCCTTATTCAGTTCT | 308 |
| VMgSSR542 | >CONTIGS_13928 | (AG)8 | GCTACCGCAGTCTATCTCATCC | CACCTGGGTTTGATCCCTC | 251 |
| VMgSSR543 | >CONTIGS_13952 | (TCTTCC)3 | GTGAGTGGAGGAACATGAACAA | GGCAATGGAGGATGGTATAGG | 343 |
| VMgSSR544 | >CONTIGS_14015 | (CAT)5 | CAAATGGGCATAGAGTTTCACA | GGCTAAAGAAATGGCAGAAAGA | 374 |
| VMgSSR545 | >CONTIGS_14023 | (GAA)8 | GAATAGGGTTAGGGGAGCATTT | GGATTGGGTTGTTGTTTTCTCT | 261 |
| VMgSSR546 | >CONTIGS_14172 | (CCGTCG)3 | ACGGAGGTTAGTTTCTCAGTGG | TCTGATGTTATCGGTGTGGAAG | 209 |
| VMgSSR547 | >CONTIGS_14292 | (CAT)7 | GCCAAGTTTTGACCGATATGTAG | TTATCACTTCCAAGACGAAGGC | 382 |
| VMgSSR548 | >CONTIGS_14310 | (TCAACT)3 | AAACCATACACCCAATTCCTGA | CTAAAGTGACCCGTTTGATTGC | 229 |
| VMgSSR549 | >CONTIGS_14332 | (CCA)5 | AGGAACCAGAAGCACCAAATTA | CAAGGACTCAAACCTCACATCA | 329 |
| VMgSSR550 | >CONTIGS_14334 | (GGAAAA)3 | TGATCGGTGATAATTGCTGAAG | GTAGACGAGGTTAGGTGCCTTG | 323 |
| VMgSSR551 | >CONTIGS_14347 | (AATT)5 | GTCATAACCATCTCTGTGGCAA | GAATTTTATTTAGCGTAGCGCC | 344 |
| VMgSSR552 | >CONTIGS_14355 | (AT)6 | ACTGGGTGAGTCTATTGTGCCT | ATGGGGACATAACCATTTACGA | 335 |
| VMgSSR553 | >CONTIGS_14374 | (TTGAAA)3 | CAGTGTGAGAACAAAGAAAGGTG | AGCAAAAGATGGCTGGATAAAC | 386 |
| VMgSSR554 | >CONTIGS_14408 | (GTTGCT)3 | GACCAGAAAACTGGCTTAATCG | TGACAATACAACACCTTCACCC | 377 |
| VMgSSR555 | >CONTIGS_14418 | (GAA)5 | GAGAAAGAAACAATGGTGGGAG | GGCATAGTAATCGTCATCGTCA | 255 |
| VMgSSR556 | >CONTIGS_14446 | (TTTA)4 | GAAACAATGGGGTAACTGCTTC | AACTCCGTTCAAATCTGTGGAT | 383 |
| VMgSSR557 | >CONTIGS_14466 | (TGA)7 | CAGATATGCCACAATCACGTTT | TCTCTTCTCTGCTTCTTGCTGA | 294 |
| VMgSSR558 | >CONTIGS_14752 | (TGATT)4 | TTCGTTGAATCTGTTGAAGGTG | AATTCTGATGGTGATGCTGAAG | 163 |
| VMgSSR559 | >CONTIGS_14755 | (CGG)5 | CAGCAGATCACAACCTTCAGAC | AGAACGGGAAATAAGGGACAAT | 318 |
| VMgSSR560 | >CONTIGS_14765 | (TGGAA)8 | GGAGTGGAGTGGACTGGAGTAG | TCAGCACACATTTCTTTTCACC | 122 |
| VMgSSR561 | >CONTIGS_14772 | (GGGTTC)3 | AGCCCGTAACCAAGCCTATACT | ACGAGAACAGAGAGAAGGTTCG | 376 |
| VMgSSR562 | >CONTIGS_14824 | (ATTT)4 | CCCTCCACATAATCTCATCACC | ACACAGTTTTGTAGCCGTTGTCT | 278 |
| VMgSSR563 | >CONTIGS_15030 | (AG)11 | ATCTTGGGTGAATATGACGGAG | GTAGAAGTGGGTTTTGACCAGC | 315 |
| VMgSSR564 | >CONTIGS_15045 | (AC)6 | GATTTCGTACACCCCAACAGAT | GCTTAACAGTCAGATTCGTGGA | 372 |
| VMgSSR565 | >CONTIGS_15049 | (TCT)5 | CAACCAACTCACTTCCACTCTG | GTTGAGGATTGAGGGTGCTAAC | 311 |
| VMgSSR566 | >CONTIGS_15075 | (CT)9 | CTCATCATCCCCTGAATTTGAT | AACCTTTCATCTTGTTGCTGCT | 300 |
| VMgSSR567 | >CONTIGS_15117 | (CT)6 | GCCACACACCCTTCTTCATT | GCTGTTGTTGAGTACAGAGCGT | 248 |
| VMgSSR568 | >CONTIGS_15145 | (AAG)6 | CTCTGTGTGTGTTTGGTGAGTG | CAAAAGGAGAAGGACGATGATT | 235 |
| VMgSSR569 | >CONTIGS_15230 | (CTT)8 | AAGGGTTTCTCCAAGTAAAGGC | ACAATGAGGTGGAAGAGAGGAA | 350 |
| VMgSSR570 | >CONTIGS_15344 | (CAA)5 | GCGCTAACTTCTTCACAACTTCA | GTTACCCTGTTCGTTCACGTTC | 293 |
| VMgSSR571 | >CONTIGS_15435 | (AGA)5 | TACTCCTTCCCTCTCTCTCCCT | AGAATGACCCATCATATCGGAA | 204 |
| VMgSSR572 | >CONTIGS_15453 | (TCT)5 | CTCTTTCCTCTTCAACACCACC | GGGTATCTTGCTTACAGTTGGG | 184 |
| VMgSSR573 | >CONTIGS_15515 | (GA)7 | TGAAAGTTGGATTGATGGACAC | GGGTTTGTTGCACATTTTCTCT | 398 |
| VMgSSR574 | >CONTIGS_15520 | (TCTGTC)3 | TGCAGGTACTAATCACATTGCC | TATTGAAAGAGGAAAGGACCGA | 244 |
| VMgSSR575 | >CONTIGS_15533 | (CCGCCT)3 | AGCTCCCTGATTTCACTCCC | CAACTACGGCACCACTTGTCT | 354 |
| VMgSSR576 | >CONTIGS_15648 | (TGC)7 | GAAAGATCACTTCCAATACGGC | CAAACACACCTTCATTGTCCAG | 176 |
| VMgSSR577 | >CONTIGS_15764 | (CAG)5 | AGGGGTAGTAGGAAGGAAGGTG | CATGTAGACAAAAGCCAACAGG | 299 |
| VMgSSR578 | >CONTIGS_15828 | (GGAG)4 | TCCGAGTTTGGTTGCTTGTATT | CCTCTATCCAACATTTCATTCACC | 309 |
| VMgSSR579 | >CONTIGS_16095 | (GGTTCA)3 | TGGTTTCTGTTTGTAATGGTGC | AGTCAGGTCAAGAACAAGCCTC | 269 |
| VMgSSR580 | >CONTIGS_16104 | (GCC)5 | GTCTGCCATAAACAAGCACAAA | GCTCCGAAAGAGAAGAAGTTGA | 288 |
| VMgSSR581 | >CONTIGS_16120 | (TCA)12 | CTACAAGAGGCCAGTGTGAATG | AACCACTTAGGTTCAGCCCATA | 386 |
| VMgSSR582 | >CONTIGS_16317 | (GAAA)4 | GCATGTGGGCTATAAATTGGTT | GACAACAGCATCTATGACAGGC | 268 |
| VMgSSR583 | >CONTIGS_16353 | (GGAAGT)3 | ACAACGCTACTTCCAATTCCAT | TAAATCATCCAACGACTTGCAC | 317 |
| VMgSSR584 | >CONTIGS_16375 | (ATG)5 | GTGCTTTTGGTTGATGAAGTTG | CGTTACCACCCATTTGTATTCC | 280 |
| VMgSSR585 | >CONTIGS_16392 | (GA)7 | AGTCTTCCTGAGAGAGCACGA | ATAGCAATCCTCGTCCGTACA | 150 |
| VMgSSR586 | >CONTIGS_16401 | (TAGA)4 | GGGTAGCATTAGTCACACACACA | CGATTTTGTTCATAGCCTCTCC | 107 |
| VMgSSR587 | >CONTIGS_16557 | (CAC)5 | CTAGAGCAGCGTTTGGGC | AGGGTTGAATGGACTTTAGGGT | 135 |
| VMgSSR588 | >CONTIGS_16566 | (CCAAAA)3 | AAGAGAGCCGTGAACGTAAAAC | GTTGCGGGATAGAAAAGAGTGT | 111 |
| VMgSSR589 | >CONTIGS_16567 | (AAGCTA)3 | CTGTCTGCGATGTATTTTCTGG | CGTGGTCTCTATGTCCATGTTG | 244 |
| VMgSSR590 | >CONTIGS_16607 | (TC)8 | TTTTCCTTCTCTTTCTCTCTCTCTC | GCCACAAGTTTTCTTCCTCC | 214 |
| VMgSSR591 | >CONTIGS_16610 | (GCG)7 | AGAGGAGTTTTGGGGAGACATT | GACTGCCATCACTTATCACCAC | 139 |
| VMgSSR592 | >CONTIGS_16635 | (ATCAAC)3 | GTTGGTGGAACCTGTTAAGACC | AGTGCTGTATTCCGTAGCCATT | 224 |
| VMgSSR593 | >CONTIGS_16730 | (GAACCC)3 | ACTCTTCAAACCATGACGCTCT | AGAAGCAGCACTATGACCATGA | 378 |
| VMgSSR594 | >CONTIGS_16813 | (GCA)6 | CAAAGTAAGATGAACGCTCCCT | GGATTTTCAGGATGTTTGGTGT | 152 |
| VMgSSR595 | >CONTIGS_16815 | (TCT)5 | CCATGAAAATAAGACGGGTGAT | TAGTGGATGTGCGAAGGTATTG | 192 |
| VMgSSR596 | >CONTIGS_16845 | (TGC)5 | GAGAGAGTAAAGGAGGTCCCGT | CCACAAGAGAAGAGGTTATCGG | 271 |
| VMgSSR597 | >CONTIGS_16850 | (CAT)5 | CCCACTGTTGAGACCACTGTAA | CCTTCTAGCCGTGCTTCTTCTA | 349 |
| VMgSSR598 | >CONTIGS_16853 | (TCT)6 | CCCACCTTTTACTTTTCTCTTCC | ATCGTTGTCTCGGTCGATTAG | 349 |
| VMgSSR599 | >CONTIGS_16863 | (TG)14 | CCTAACATTAACAGCCCTTTGG | AGTATTTCCTTCCCATTCAGCA | 159 |
| VMgSSR600 | >CONTIGS_16885 | (TTCA)6 | TAAAGGCCAACAAATGCCTCTT | ATCATTCAAATCCTGCCACAGA | 177 |
| VMgSSR601 | >CONTIGS_16898 | (AACAG)4 | ATGAGAAACAAGGTGAAGGACA | CAAGTAGTTTGGAGCTTGGGTT | 147 |
| VMgSSR602 | >CONTIGS_16935 | (AG)6 | GTTACAATGCACACAAACAGCA | GGACCCTCTTATCGGACTCTAA | 203 |
| VMgSSR603 | >CONTIGS_16937 | (TTTTG)4 | CTGAGTAGCTGGGACTAGGGG | CCTGGGCAACATAGGGAGA | 109 |
| VMgSSR604 | >CONTIGS_16981 | (AG)6 | TTCTTCCTCCAGTTCTCTCTGC | GAAGCTCATCTCCTACACCGAC | 253 |
| VMgSSR605 | >CONTIGS_17000 | (TTC)8 | CGATAACATCTTCGGCATTACA | ATCTGAACCTAGCAGAGGGTTG | 126 |
| VMgSSR606 | >CONTIGS_17005 | (CAAAAA)4 | AATGGTAGGAAAGGTGCAAGAA | TTCGGGCATTTTGTCTGAGTA | 311 |
| VMgSSR607 | >CONTIGS_17020 | (TG)7 | ACATCCTAAACTTCTGCTGCCT | GCGAACTACAACACTCCTGTTT | 221 |
| VMgSSR608 | >CONTIGS_17058 | (CT)6 | GAAACTTCTGATCTCGGCCAC | ACCATTTTGGAAGCATAACTCG | 202 |
| VMgSSR609 | >CONTIGS_17077 | (CTTC)3.25 | TTCTTTCTTTCTCTCTCTCTCTCTCTC | GGTGAAACCCCGTCTCTACTAA | 213 |
| VMgSSR610 | >CONTIGS_17145 | (CTC)5 | ACCCTCACTCTCTCCCTCTCTC | GCCCCTCTTGTTTCTCTTATCC | 297 |
| VMgSSR611 | >CONTIGS_17212 | (CCAGCA)3 | AGTATGTTACCTCAGCGTGCAG | CTGTGCAACCCAATTATCTCAA | 283 |
| VMgSSR612 | >CONTIGS_17214 | (TGTCTC)3 | AGTTCATGTTGTCATCGTCCTG | ACAAAGACCTCCCTGAAGCATA | 291 |
| VMgSSR613 | >CONTIGS_17230 | (CGG)7 | TTCGGTCCATTAGTATGAGCGT | AACAACACTCTGCTCCACTTCA | 268 |
| VMgSSR614 | >CONTIGS_17237 | (CGA)5 | CTACGCCGAGTGCTCCTC | CGAGTCCTTCAACTTCGGTATC | 152 |
| VMgSSR615 | >CONTIGS_17307 | (CT)15 | GGCTTGTATTTGTAGCACAGCA | GGAATCGAAACCAGAGAAGATG | 190 |
| VMgSSR616 | >CONTIGS_17395 | (GT)13 | GCACATTCTCATCAACATTTGG | CAGTGAATAGCAAGTTAAAGCCAC | 121 |
| VMgSSR617 | >CONTIGS_17405 | (CTCAAT)3 | ACCGTCATCACCACAATATCAC | AGTAGGACTGGAGACATGCACA | 196 |
| VMgSSR618 | >CONTIGS_17406 | (AGGGGC)4 | TGATGGTTCTGCTATTGTTTGG | TTAGCCTCCATTCGCTCAAC | 358 |
| VMgSSR619 | >CONTIGS_17454 | (CCCTAC)4 | GCAAAGGTAGTGAGAGGATGGG | GTGGTCATTGGCGGTGCT | 208 |
| VMgSSR620 | >CONTIGS_17504 | (CCAATC)3 | TGGGGAGGATATGGATACGTGA | CCCGAGGGAAAGCCCAAT | 371 |
| VMgSSR621 | >CONTIGS_17513 | (TC)7 | GCCTCAGATGTTTGGAGTTTCT | GAAGGTAGTGCTAGGTGCGAGT | 119 |
| VMgSSR622 | >CONTIGS_17515 | (CGG)7 | CCACCAACTCCTCTCAACTTCT | TTCTCGTTCAGCTCCTCGTAGT | 105 |
| VMgSSR623 | >CONTIGS_17551 | (GCC)5 | CCCTAGAAAGAGGATGAGAGCA | CAACGGAGGACTTTGGATCTAT | 257 |
| VMgSSR624 | >CONTIGS_17627 | (GGAACG)3 | CATTGATGGTTTGATACACAGG | TGCTTCTTCACTTCCTTCACTT | 149 |
| VMgSSR625 | >CONTIGS_17632 | (CAAAAC)3 | CTTTTCATTCACCCAAACCCTA | GTTGATCGGAGAGAGTGAGACC | 325 |
| VMgSSR626 | >CONTIGS_17678 | (CTG)5 | GTCCCAACTCTACAATCCCAAC | GTCGGAGGTAAAGGCCGTA | 400 |
| VMgSSR627 | >CONTIGS_17724 | (CCA)6 | ATGAAAATAAAGGTGGGTGGTG | CCGTGGATCTGGAAGGTAAATA | 318 |
| VMgSSR628 | >CONTIGS_17750 | (TTC)5 | AGTGGGATTTGTTGTAGATGGG | CGTTTTCTCCTCTTAACCCAGA | 213 |
| VMgSSR629 | >CONTIGS_18375 | (TC)6 | AAACCAAACCAACGCCAAC | GCTGCTTCTTCAATGGCTATCT | 112 |
| VMgSSR630 | >CONTIGS_18497 | (CACGGT)3 | ACTTGAATGAATGGGAAACTGC | GCGTGTTGTTGCTATTGTTACC | 233 |
| VMgSSR631 | >CONTIGS_18537 | (AG)7 | ACTGAATGAAAGACGGGTAGGA | GCCACCAACTGAACATAGAACA | 238 |
| VMgSSR632 | >CONTIGS_18582 | (AG)6 | CACTCGCCTCACAAAGGATATT | GGAGTTCACTAATGCCAAAAGG | 383 |
| VMgSSR633 | >CONTIGS_18647 | (GGA)5 | GAGGAGAATACACTGGAGGTGG | AGCTGGATCTCGGCAACA | 262 |
| VMgSSR634 | >CONTIGS_18665 | (TC)6 | GAAGATGTTGACCACTCACAGC | CCAAAATGCAGACGTGGAC | 197 |
| VMgSSR635 | >CONTIGS_18847 | (TGC)5 | AGTCGGGAAGAAATTATGGGAT | CATGAGATGGTCAGGATCAACA | 153 |
| VMgSSR636 | >CONTIGS_18926 | (TGA)5 | AAGTGGAGCTGCCGTAGATAGT | TTAGCATCATCGTCTTCCTCCT | 135 |
| VMgSSR637 | >CONTIGS_19089 | (GAA)5 | TCTAATGGAGAAGCTGAGGAGG | GCTCACATGACAGAACAAGCAT | 348 |
| VMgSSR638 | >CONTIGS_19112 | (TCA)5 | GGGTTCTCACCCTACTGTTAATG | GAGATCAAGACGAGACCGAACT | 127 |
| VMgSSR639 | >CONTIGS_19149 | (GCG)6 | ACGGATTCTCCAACACTACCAC | TTTTCACCACCTCTCACCTTCT | 139 |
| VMgSSR640 | >CONTIGS_19178 | (TC)6 | TTAGATTGCAGGTTTCCATGCT | GTCAGCTCATAGTTCTGGTCCC | 226 |
| VMgSSR641 | >CONTIGS_19334 | (TCGACC)3 | GCGCTTTACTGAGCCTGTC | GTCACGTTGCTGTCGGTC | 139 |
| VMgSSR642 | >CONTIGS_19358 | (GAT)6 | GCACGAGAGATAAAGAAAAGGC | TTGCTTACCCAACCTCTGATCT | 159 |
| VMgSSR643 | >CONTIGS_19430 | (ATGGTG)3 | CACCATGCTCTCTTTCTCTGTG | TTCTCAGTGGTCATTTTGCTGT | 187 |
| VMgSSR644 | >CONTIGS_19494 | (AG)7 | CGGGCTTCACAACGATATAAAA | TTCCCTTTCCTCTCTCTTCCTC | 131 |
| VMgSSR645 | >CONTIGS_19512 | (GGTTTT)3 | TGGTTTTCGGAGGACAGTAAGT | GACAAACAAATCCCCAATGTCT | 273 |
| VMgSSR646 | >CONTIGS_19517 | (ACC)6 | CTCTCCCTCTCCACAACCACT | GGGAACGAGATTACCGAAGAG | 251 |
| VMgSSR647 | >CONTIGS_19518 | (GA)7 | AAAAGGTATGGTTGGCTTGTCT | TATGTTGGACTCTGCCTTGATT | 224 |
| VMgSSR648 | >CONTIGS_19543 | (CA)6 | ATCCTAACAAGCGTCCTACAGC | CAAACGATACAACTCACGGAAG | 242 |
| VMgSSR649 | >CONTIGS_19786 | (TCT)9 | CCTACCAACAGTACACCCCAAT | TCTCTTCTTCTTCCTCTCTGCG | 200 |
| VMgSSR650 | >CONTIGS_19803 | (GCA)5 | GCAGAACATGAGTGGAATGAAA | CCTAACCAGACACACCAGACAA | 396 |
| VMgSSR651 | >CONTIGS_19853 | (ACTC)4 | AATTGTGGTGCTCATACATCCA | CCACCTCCTACTTCAAATCCAT | 337 |
| VMgSSR652 | >CONTIGS_19874 | (GAT)5 | ATTGGTCTATTTGAATCCGCAG | ATTGGTTATGGTGCTAGGGTTG | 203 |
| VMgSSR653 | >CONTIGS_20129 | (CT)6 | TTAGCCTCGTTTTCCTTTGCT | GATGGGATTGGGAATAGAATGA | 109 |
| VMgSSR654 | >CONTIGS_20225 | (CAT)5 | TGGCAACAGTGAGGGAACT | TGTGCTGTCTTGTCTCAGGTTT | 165 |
| VMgSSR655 | >CONTIGS_20358 | (GACGAG)3 | GCAGCTTGTTCTGCACCTG | AGATGAAGGGGATCGACCA | 132 |
| VMgSSR656 | >CONTIGS_20403 | (CT)6 | GAACCACCTTCCTCATTCTCAC | CGATCTGTCATTCGTTCTACCA | 344 |
| VMgSSR657 | >CONTIGS_20424 | (GTA)7 | AGCAGAGTAGCAAAGGAACCAG | GACAGAAACACGTTAGAAGGGG | 290 |
| VMgSSR658 | >CONTIGS_20533 | (TAT)5 | CATCATTGCCTCCACTGTTACT | CATTGGCTACAGACACCACCT | 149 |
| VMgSSR659 | >CONTIGS_20536 | (TCA)6 | TGATGCAGAAACATGGGATAAG | AGTCTTTGGCTTTGAACCTGAA | 380 |
| VMgSSR660 | >CONTIGS_20566 | (TGA)5 | ATACCCCTTTCAACCTTCTGCT | GGAAGAAATCCAAGAATGATCG | 220 |
| VMgSSR661 | >CONTIGS_20637 | (CAA)6 | TCTCCATAATCCCCACTTTCAC | ACACTGCATAGCCAAAATTCCT | 399 |
| VMgSSR662 | >CONTIGS_20648 | (GTGGCC)3 | GAGGAAGAGACCGTAAAGGGTT | CAGTGAGGATGAGCAGAAGTTG | 336 |
| VMgSSR663 | >CONTIGS_20668 | (ATG)5 | AAGAAGCAAAAGGGTCGTGATA | AATCCCACAAAGAACTCCTGAA | 308 |
| VMgSSR664 | >CONTIGS_20669 | (TC)6 | GAGCGTCCCTGTCAAATCTTA | AAGGAGGTAGACCCAGAGAGAGA | 153 |
| VMgSSR665 | >CONTIGS_20694 | (TCGCCG)3 | AATCCAACCTGAAGAAGTCCAA | AACGTAGAGTGGCCTAAAGCTG | 238 |
| VMgSSR666 | >CONTIGS_20951 | (AAGCCC)4 | CACAGCTCCGACACTCTCAC | AATTCAGGTTCAGGTTGAGGAA | 341 |
| VMgSSR667 | >CONTIGS_20953 | (TATG)4 | TTGAGATCCCTTCTCGTCTCTC | CTCCTACAGCGGAAACAACTCT | 340 |
| VMgSSR668 | >CONTIGS_20988 | (CTGCTC)3 | CAAAGGGATTCAGGTTAAGGAA | CGTACTTCTCCACCTTGCTTTT | 203 |
| VMgSSR669 | >CONTIGS_20997 | (AG)9 | GAAAATGAAACAATGACTGCCC | CTTTCCTCGTGTGCTCTCTCTC | 137 |
| VMgSSR670 | >CONTIGS_20998 | (TA)9 | GGACGAAGATAGTTGGATATGTATG | GTGTCTTTGGACAACCAAGGTA | 223 |
| VMgSSR671 | >CONTIGS_21027 | (CT)10 | ATCACCACTGAGAGAGATGGCT | GACAAGAGAATGACACGCAAAA | 324 |
| VMgSSR672 | >CONTIGS_21053 | (TTCTC)4 | TTGTCTTCCTCTTCTCCTTTCG | GATTGTGTTAGGACCCAGGATG | 132 |
| VMgSSR673 | >CONTIGS_21069 | (ATG)5 | ACGCCCTGTAGAACAAATGAGA | AAACTGATTCTGGGATGTTTGG | 250 |
| VMgSSR674 | >CONTIGS_21183 | (AG)12 | AATGAAAGATGGCGAAGAGAAG | GAACATGAATACAAAGCAGCGA | 365 |
| VMgSSR675 | >CONTIGS_21229 | (AAG)9 | TCACCAGTTTTATGCACCAGAG | AGTGTTTTGGATTATGGATGGG | 191 |
| VMgSSR676 | >CONTIGS_21230 | (TTGTGG)3 | AGGTACATTCGGAGGAGGAACT | ACCAACACGGTCAATAGGGATA | 123 |
| VMgSSR677 | >CONTIGS_21387 | (GCT)5 | GACAGAGGCAGCTAATTTGAGG | GAGCAAAAGCCGAGAAATAGAA | 208 |
| VMgSSR678 | >CONTIGS_21418 | (GT)7 | TGTGAATGTGTTAGGAAGAGTGAAG | TGTATTGCAGTGGGAATTGGTA | 348 |
| VMgSSR679 | >CONTIGS_21542 | (AGA)9 | AGCGTGTGTGGTTTTGGTAACT | ATATAATTTCAGCAGCGCGAGA | 220 |
| VMgSSR680 | >CONTIGS_21689 | (TCT)8 | CGCCATCATCTCTATCATCTTG | ATGTGCTGTGGAAGAAAGGAAT | 250 |
| VMgSSR681 | >CONTIGS_21887 | (GA)9 | GGGGTTATGCAGATATGAGAGA | TTCCAACACCAATTTCCTTC | 145 |
| VMgSSR682 | >CONTIGS_21899 | (GTT)5 | TGAAAGCGATTGTTGGTATCAC | CGTGCTCTCTCTCTTCTGTCAA | 228 |
| VMgSSR683 | >CONTIGS_21942 | (CCT)6 | CCAAATGACCACACATAACACC | AAAAGTTGAAAACGAGCGAGAG | 330 |
| VMgSSR684 | >CONTIGS_21943 | (CAG)5 | GCAGCTCCGATGATAGAACTAGA | GTGAGACAAAGGCAAAAGATCA | 388 |
| VMgSSR685 | >CONTIGS_22014 | (CCA)6 | CATATCCACCACCACAAGGAG | CATGTCCAAAATGATGATGTCC | 321 |
| VMgSSR686 | >CONTIGS_22048 | (GGT)6 | CGTAGACAATTTCACCAACACG | ACGTCGAGGAGAGAAGAAACAG | 368 |
| VMgSSR687 | >CONTIGS_22073 | (TCGGG)4 | TTGGCTATGTTTCCGTAGAACC | GTTTTCCCACTCTGCATTCATT | 266 |
| VMgSSR688 | >CONTIGS_22159 | (GGGATC)3 | GTCGAGAAGGAACAGAGCAGA | TAGTTTCTAACATGGCTCGCTG | 138 |
| VMgSSR689 | >CONTIGS_22251 | (GGAGGC)3 | GATTTGACGGTGGAGGAGTTC | AGTATTAAAGCAGAGAAAGCGGTG | 215 |
| VMgSSR690 | >CONTIGS_22301 | (CCA)7 | AGTGGGTAGGTGTGGTAGCTGT | ATGGTTTGTCTTGGTGTTGTTG | 150 |
| VMgSSR691 | >CONTIGS_22303 | (CAAGAT)3 | CCGATACAATTTTCAACCCTTC | GTTCATGTCTTCCTTCTTTGGG | 173 |
| VMgSSR692 | >CONTIGS_22335 | (TTC)7 | GAAATAAGTGTGGCTGAGAGGG | AATCCAATCTACCCAACGACAT | 175 |
| VMgSSR693 | >CONTIGS_22346 | (AGC)5 | GATGGTGATTTGATGGATGTTG | TATTCAGCGAGGCTAGAGAACC | 283 |
| VMgSSR694 | >CONTIGS_22382 | (TCAT)6 | CCAAAGTGCTGGGATTACAGGT | GTGAGCCGAGATCGTGCC | 136 |
| VMgSSR695 | >CONTIGS_22390 | (AG)6 | ACAACTTTACTCACCCACGAGC | CTTCTCCTTCCGCACTTTCTTA | 197 |
| VMgSSR696 | >CONTIGS_22443 | (TC)6 | GGCCACTGTTATTGCTGCTACT | CTTTTCGAGCATTTCCTTCATC | 223 |
| VMgSSR697 | >CONTIGS_22472 | (AATCGG)3 | TTCCCTTAAAAGCAGATGGAAC | CTGTTGTGGAAGATGAAACCAA | 378 |
| VMgSSR698 | >CONTIGS_22547 | (ACAA)4 | AATGTGAACTGAACTGAGCTGG | ATACCTTGTGTGCTGATTTTCG | 144 |
| VMgSSR699 | >CONTIGS_22558 | (GTT)7 | AGCTCCCATCCTGCGAAT | GTGGTTCAAATAATCACTGGCG | 205 |
| VMgSSR700 | >CONTIGS_22653 | (CTCTTC)3 | CCGACATTCTCTTCCTCTATTCC | GCTCCGACCAAATCCATAGTAA | 367 |
| VMgSSR701 | >CONTIGS_22820 | (CCTCTC)5 | CCCTCTCTATTTGCACGAAAAC | AGGTAGGAACCCACTACGGAAT | 342 |
| VMgSSR702 | >CONTIGS_22766 | (TTCCTC)3 | AAGAAGCTCGTCCTCTTCCTTT | GAACCAACTCAACAACCTCTCC | 367 |
| VMgSSR703 | >CONTIGS_22948 | (TC)6 | TACAATCACTCTCCGTGTGGAA | GGTTTTGAAGGGGAAGGTAGAT | 244 |
| VMgSSR704 | >CONTIGS_22978 | (CTTT)4 | CCACGCATAAACACAACAACAT | GTTAAGGTACTCTGCCTCGGAA | 307 |
| VMgSSR705 | >CONTIGS_23120 | (GTTGGT)3 | TTCTTGGAGGAGAATAGCTTGG | AGCAGAGACCCTTTCACCCT | 233 |
| VMgSSR706 | >CONTIGS_23194 | (TCAG)4 | CTTGGTAGCAGCAGTCGGTC | GCTTCTTTTCCTCCCAGTCTTT | 107 |
| VMgSSR707 | >CONTIGS_23205 | (CTGGAG)3 | CTGGGAATAGGAGGTGGTAGTG | AGAACTGGAAGATGAAGATGCC | 102 |
| VMgSSR708 | >CONTIGS_23209 | (CTT)5 | TGGAGAAGAAGGTGAAGAGTGA | AAGAGTGGTGGAGAGAACAAGC | 197 |
| VMgSSR709 | >CONTIGS_23284 | (TGG)8 | AGAAACTGAGGGTACAAGGACG | GAGCAGAGTGAAGGAAATGGTT | 329 |
| VMgSSR710 | >CONTIGS_23322 | (CATCTT)3 | TCTCTTCTTGTTGGCATTCTCA | TCTGAAATTGTTGCTAAGGGTG | 171 |
| VMgSSR711 | >CONTIGS_23330 | (CAC)5 | CAGTCTTTTGCAGAGATGAACA | TGCATCAAATAAAGCAGGC | 132 |
| VMgSSR712 | >CONTIGS_23446 | (AG)8 | CGGGGTGAAATTGATACACAAG | GCTATGGACAGGACCAACAGAG | 332 |
| VMgSSR713 | >CONTIGS_23458 | (AG)6 | CTTCTCGGAACCTGATGCTACT | CCGCCACTTATGTATCTGGAAT | 299 |
| VMgSSR714 | >CONTIGS_23564 | (GGA)5 | TTGAGAAGGACGAGAAGAAAGG | TCCTCACACACTCACACAGTCA | 272 |
| VMgSSR715 | >CONTIGS_23574 | (TCCGAT)4 | GACATGAGACCGAGGGTAAGAC | TGACGACTCAGCCTAACGAAT | 246 |
| VMgSSR716 | >CONTIGS_24342 | (GTTGAA)3 | GAACCTGAAGCTGTGGCTGT | GGCGGCTCTCTCTCTACTTATTC | 185 |
| VMgSSR717 | >CONTIGS_24367 | (GGAAGA)4 | AAATAGCGCAGAGAGACCAAAG | TTATGGAAGAAGTGGAAGGTCG | 254 |
| VMgSSR718 | >CONTIGS_24377 | (ATC)8 | CAGCATTACATCTCCTCTGTCG | TGTGTTGTAGCGGACTATTTGG | 225 |
| VMgSSR719 | >CONTIGS_24443 | (TTCTCT)3 | GGAGAGCAAAATCCTTGAAATG | GGTGAGAAGAGAGAGATGGGAA | 364 |
| VMgSSR720 | >CONTIGS_24632 | (GCGCTG)3 | ATGAACATGACTTCCCCTGAAT | GGCATAGACCTGCATCGAAT | 141 |
| VMgSSR721 | >CONTIGS_24702 | (AGA)5 | GAATTTGCGAGAAGAGGAAGAA | GATAAGGCGATGCAGAAGAAAG | 150 |
| VMgSSR722 | >CONTIGS_24716 | (CA)8 | AGGTCGTAGAGAATGACGCTGT | CTCTTCACACACAGACCAAACC | 231 |
| VMgSSR723 | >CONTIGS_24763 | (TA)8 | CTTCATGTGTTCAGGGTTTTGA | TGAAAGATAGCAGCCCAGTTCT | 284 |
| VMgSSR724 | >CONTIGS_24813 | (GT)10 | ACTAAGCACACACATGCCCATA | TAAACAGCCATCATCACAAACC | 139 |
| VMgSSR725 | >CONTIGS_24887 | (GTGA)4 | GTTACTCGGACCAGCCTCATAA | AAAAGAACTGGCAAGAAACCAC | 252 |
| VMgSSR726 | >CONTIGS_24892 | (GC)6 | TGCTGATTACGCAGTCTTCTTC | AGTTGCTCCATCACATCCAG | 148 |
| VMgSSR727 | >CONTIGS_24952 | (TC)7 | AATACAAGGAAACAAGGGCAAG | AACTTACAATCAAACCGTGGCT | 170 |
| VMgSSR728 | >CONTIGS_25047 | (CA)7 | TGTCTCTGTCTCTGTCACTCTTTCTC | TCTGTTCAGGAGCTGTGTGAAT | 100 |
| VMgSSR729 | >CONTIGS_25085 | (CCG)7 | CCTCCCACTACTGATTGCCTAA | GCCGTAATTCACCCATTTGTA | 374 |
| VMgSSR730 | >CONTIGS_25102 | (GGC)5 | TTGAAGAGTTTTGCAGTGTTGG | GTTTGACCTTCCTCCTTTGATG | 189 |
| VMgSSR731 | >CONTIGS_25310 | (AAG)5 | CGTAAGGTTGTTGTTGTGGAAA | AAAGCAGGTTCTGGAATGAAAC | 381 |
| VMgSSR732 | >CONTIGS_25337 | (CT)6 | TCTGGTCTCAGGCACAAAGTTA | AGTAGAGAAATGGCTTCTTCGG | 120 |
| VMgSSR733 | >CONTIGS_25495 | (GCT)5 | CTCATCGGCCTTCATAATCTTC | CACAACAACACAACACTCAGCA | 294 |
| VMgSSR734 | >CONTIGS_25835 | (AG)6 | CGTCTGTGTACGTGGCATAAAT | CTGCATTGTGTGTTGCTTTTCT | 258 |
| VMgSSR735 | >CONTIGS_25905 | (CA)9 | GTCACTGGAGGGGAGCTGT | TGATGGAAAGGTGTGAGAGAAA | 226 |
| VMgSSR736 | >CONTIGS_25950 | (TTG)7 | ACCGTTGTTGTTGCTGTTGTTA | GGTGGCATACACTCGAAACTACT | 229 |
| VMgSSR737 | >CONTIGS_25970 | (CGTCGC)3 | ACCTATAAAGCTCGACCAGGC | GGAAAGAAGAGAGAGCGGAAAT | 145 |
| VMgSSR738 | >CONTIGS_26088 | (GA)9 | CTTTTGGGGTTGGAAGAGTGT | CTCGTACAGGCGAACAACATAA | 288 |
| VMgSSR739 | >CONTIGS_26176 | (ATA)5 | GGAATCGGACTTGGAAGAATAA | CATTGTCACTTTGCACCTTACC | 133 |
| VMgSSR740 | >CONTIGS_26224 | (AGG)5 | CATGTAGAACACACGCCTTACAA | AGTTTACCACTCTCCTCCACCA | 294 |
| VMgSSR741 | >CONTIGS_26231 | (TCC)5 | ATCCCAACCATACGTTTAATGG | CCTGAGAGGAATTTTGGTCAAC | 387 |
| VMgSSR742 | >CONTIGS_26328 | (GCTG)4 | TTGCTTCCAAAATCTCTCTGTG | TTGTTCCCAGTGATAAGGGAGT | 363 |
| VMgSSR743 | >CONTIGS_26396 | (GA)6 | GCTTCCTCACATGGCAGAG | GGGGCCTGCATTAAAGTAAAA | 146 |
| VMgSSR744 | >CONTIGS_26500 | (CGT)5 | CACTCACTCACTCACTCTCGGT | CGCTCAGGTTATCCACTCTAGG | 153 |
| VMgSSR745 | >CONTIGS_26576 | (AGG)5 | ATTCTTTCCTCTTCCTCCGAAA | CGTTACCGTTTACTGTTCGTCC | 103 |
| VMgSSR746 | >CONTIGS_26602 | (TGATCG)3 | GAGTGGTAGTGGGAATGGGATA | AACAACTAGACGCCGAGAGAAG | 123 |
| VMgSSR747 | >CONTIGS_26646 | (GGT)5 | GGAAGCCGAGGGAGGATG | AAATGCTGGCCCTTCTCAATTA | 127 |
| VMgSSR748 | >CONTIGS_26767 | (CCA)6 | CCTCCCACTTCCTCCTCTACA | GAGAATGGATTGATCTTGACCC | 207 |
| VMgSSR749 | >CONTIGS_26773 | (GTC)6 | CTATCACTTCCCTCATCAACCC | TTTAGAAGGGCACCACTCACTT | 245 |
| VMgSSR750 | >CONTIGS_26887 | (CGA)5 | CTGGAAAATGGTACATAGCACG | ATGAGAGAAAGGGAAAAGGAGG | 149 |
| VMgSSR751 | >CONTIGS_26945 | (GTCT)4 | CAACGCAACCAGCTAATAAACTC | CCTATCGTTCATACCTCTTCCG | 194 |
| VMgSSR752 | >CONTIGS_26983 | (GGCCGA)3 | ACCAGCTTCTCTTCCTCCTCTT | TCTTCCCTCTTCTCCTTCTCCT | 149 |
| VMgSSR753 | >CONTIGS_27102 | (TGATTC)3 | AGCTCCCTTTTGGAAGATATGC | GACCAACACCTCTGACTGTGAA | 196 |
| VMgSSR754 | >CONTIGS_27140 | (AC)7 | TTGGTGGCCTTTGATTGTAA | CTGAATGGCAAGATTTTAGGTG | 119 |
| VMgSSR755 | >CONTIGS_27167 | (TGG)5 | ATTCATCCTTGTCTGTTGTTGG | CTGCACCTCCACTGTTATTCAT | 159 |
| VMgSSR756 | >CONTIGS_27262 | (CAG)7 | TAACTCATCAGCATTGACAGCC | CGGAATGTGGAACCTATTTCTC | 219 |
| VMgSSR757 | >CONTIGS_27297 | (ATTT)4 | GATTATAGGCATGAGCCACCAT | AGGAGAATCGCTTGAACCTG | 172 |
| VMgSSR758 | >CONTIGS_27351 | (CT)6 | TCAAGTTCACAAGTGCCAAAAC | GAGAGTCCCCAAATGAAACAAC | 372 |
| VMgSSR759 | >CONTIGS_27559 | (AC)6 | CTTATGTGTATTTCGGCCTGTG | CTGATGATTCCAGGGTACAGC | 231 |
| VMgSSR760 | >CONTIGS_27610 | (CATCTT)3 | TGGGAATAGTGTGTAAGCAACG | GTAGAAGACGCGAAGAGGAAAA | 241 |
| VMgSSR761 | >CONTIGS_27635 | (GCT)5 | CGTTGGATTTGGCGTTACC | AATGGATGAGTTCGGTGTTTTG | 275 |
| VMgSSR762 | >CONTIGS_27720 | (CCGGTT)3 | GGATTTTAGGGTTTCAGGGTTT | TAGTGGGAGGAGGACAACATTC | 357 |
| VMgSSR763 | >CONTIGS_27724 | (GT)7 | CTCAGGGTCATTGTGAGGGTAG | TATCTGCTCTGGCTGAAGTGAA | 103 |
| VMgSSR764 | >CONTIGS_27737 | (AT)6 | GTGGGTATAGCAGCATTTGTGA | GGTGCATAAGAAAACACCAGAA | 319 |
| VMgSSR765 | >CONTIGS_27778 | (GTGGGA)3 | CGTGGGAGGAGTGTGATGATA | TATTATTGGACGCTTCATTCCC | 156 |
| VMgSSR766 | >CONTIGS_27853 | (GAG)5 | AGACGCTTTGATGGAAGAAGAA | CTCCACTCCTTGTTGCTGTTTT | 106 |
| VMgSSR767 | >CONTIGS_27859 | (CAA)5 | TGAACCTTGTTATGTGCATGG | AAGCCTGATATGAATCTGCAAG | 165 |
| VMgSSR768 | >CONTIGS_27891 | (TCTAAA)3 | CAGCTTCATCAAAAGGTAGCAG | TTTTCTGGGGACATAGAGGAAA | 169 |
| VMgSSR769 | >CONTIGS_28076 | (GTT)5 | GAGACAGAAACAGCATAAATGTTCC | GAGATTGGCATATTCAGCATCA | 167 |
| VMgSSR770 | >CONTIGS_28151 | (CTTTTT)4 | GAAGGAAAAGGAGGAGGAGAAG | GGCAAAGGATGAAGAACTGAGA | 283 |
| VMgSSR771 | >CONTIGS_28448 | (TCC)5 | GAAGGTGAAGAGGGGAAGAAG | AGTGCAGATATTACAGGATGGAGA | 220 |
| VMgSSR772 | >CONTIGS_28505 | (GATTGG)3 | GACGGCATCGTTTTGATTTT | CAACAACTTTCTTCATCCCACA | 128 |
| VMgSSR773 | >CONTIGS_28587 | (CTT)6 | AGAGAAGGTAATGAAGGGGCTC | AAGGAAGTGTTTGCGAGAAAAG | 337 |
| VMgSSR774 | >CONTIGS_28592 | (CTT)5 | CCTCTCTCTTTCTTCCCACCTT | CCTGTGAGCTTTTGATGTTCTG | 228 |
| VMgSSR775 | >CONTIGS_28726 | (GCA)5 | AGGAGGAGTTTTCTTTTGTTCG | GGCTGCTGTTGTTGTGGT | 183 |
| VMgSSR776 | >CONTIGS_28740 | (CAAA)4 | ATAAAACACCGAGCAGCAACAT | TGATACCTCAAGGCAACCTCTC | 289 |
| VMgSSR777 | >CONTIGS_28774 | (CTCACC)3 | TTTTCACCTTCATACCCATTCC | AGCGACAGCTACCATCTTCAC | 160 |
| VMgSSR778 | >CONTIGS_28891 | (AAG)7 | AGCCGAGGTTGGTAAAGTAAGG | TACACTATTCCCTCCCCTCTCC | 168 |
| VMgSSR779 | >CONTIGS_28915 | (TTG)6 | TGATGCTTCTTATCTCCACGAA | TAGGACAACCAAGTGAACAACG | 112 |
| VMgSSR780 | >CONTIGS_28964 | (TTTC)4 | GAAGGTATGGCAAGGTAGGACA | ACAACCTGCTCTCACAAACTGA | 116 |
| VMgSSR781 | >CONTIGS_29064 | (GAT)5 | TTGCTTTACTCGGACAGTGATG | TCCTCTTCTTGGTTTTCTTTGC | 169 |
| VMgSSR782 | >CONTIGS_29111 | (CTTCAA)3 | GTTTCCCTGATGCTTTCATGTT | CATGCTTGGTGAGAGACAAAAG | 201 |
| VMgSSR783 | >CONTIGS_29209 | (GCC)5 | TGTATAATGATCCGCAGCACC | GAACATCGTAATGAGGAGGAGG | 222 |
| VMgSSR784 | >CONTIGS_29281 | (GCGATT)4 | CTCTTTATAGCCTGCCACAACC | ATCTTCTCCCTCAATCAAACCA | 359 |
| VMgSSR785 | >CONTIGS_29285 | (TGG)5 | CTGTGATTTTGGAGCTTTTGC | CCTCCTTCATAGTACCAGCATGT | 141 |
| VMgSSR786 | >CONTIGS_29288 | (CGC)5 | TTTCTGACCCGTACAAACCC | AGGCAGCTTTCCTCGACAT | 350 |
| VMgSSR787 | >CONTIGS_29291 | (CTTCAA)3 | TCCAAGGAAGAAGATGCAAGTA | AGCATGGAAAGAAGGAAGTGAG | 169 |
| VMgSSR788 | >CONTIGS_29434 | (AG)6 | CGCTCACTTTTCCTCTCTGTTC | TGTTATTGCCATCGACCCTTAT | 118 |
| VMgSSR789 | >CONTIGS_29686 | (GA)12 | CTCTTCGGTGAGTTCTTTGGAC | GTAGACATTCAGGAGCAGGAGG | 353 |
| VMgSSR790 | >CONTIGS_29716 | (TC)6 | TTCTATAACTCTGGAATGAGGCTG | TATATCCATACCCCTTGCCC | 149 |
| VMgSSR791 | >CONTIGS_17750 | (TTC)5 | AGTGGGATTTGTTGTAGATGGG | CGTTTTCTCCTCTTAACCCAGA | 213 |
| VMgSSR792 | >CONTIGS_17860 | (GT)13 | AAGTGCTGGGATTACAGGCAT | CTGAGTGACAGAGCAAGACCCT | 166 |
| VMgSSR793 | >CONTIGS_17923 | (TGG)6 | CCTACGAGGTTTTGAGTGATCC | TGCAAGGCAACCACTTCTCTA | 345 |
| VMgSSR794 | >CONTIGS_17966 | (GATT)5 | GAAGGAACATAAGTGGTGGGAG | ATTACAAAAGAGGAGGGGCTTC | 399 |
| VMgSSR795 | >CONTIGS_17984 | (TAA)6 | AATCTTGGCACCACCACCT | GAAACGAGACTGGGGTCTACAT | 173 |
| VMgSSR796 | >CONTIGS_18030 | (AT)12 | CTTTCTTAGCACCCTGATTTCG | AGTGTGTGTGTGTATGTGTGTGTG | 174 |
| VMgSSR797 | >CONTIGS_18055 | (ACCATC)3 | TTCTGGCAATAAACTCATGTGG | ATCAGCACCTCTCAACATTCCT | 239 |
| VMgSSR798 | >CONTIGS_18165 | (TC)11 | GAGCGAGAAGAAGCAGAAATGT | AAGAGCAGAGAGGGGAAGAAG | 342 |
| VMgSSR799 | >CONTIGS_18204 | (CCATTC)4 | TCTCCCCACCTCTATACTCCTG | ATTCTTTGAGGGATGATGGTTG | 198 |
| VMgSSR800 | >CONTIGS_18222 | (CA)8 | ACTTGAGTTCGAGAGGTTCGAT | GTATTCGCCTAAGTTTGAACGC | 198 |
| VMgSSR801 | >CONTIGS_18229 | (CT)9 | TGAAACAATGCCCAATACAGTC | GAAAACACATCACGAGGAAGGT | 124 |
| VMgSSR802 | >CONTIGS_18339 | (TG)6 | ATGCTAGTCGAAATCCATCACA | ACTTTCTATCACACACGCCATT | 158 |
| VMgSSR803 | >CONTIGS_29873 | (CCGGCT)3 | CCAGACATCAACTTCAACGTGT | CAGTCCTCACCTTTCCTTGGTA | 198 |
| VMgSSR804 | >CONTIGS_30130 | (GTTAGG)3 | ATAATTGCTGCTGTTGATGGTG | TGTCGTGCTGTTCTTCTTCAAT | 102 |
| VMgSSR805 | >CONTIGS_30415 | (CGC)6 | GCGCTATTTGGCGACTACAC | AAACTCCCGTTCCCTTCTATGT | 176 |
| VMgSSR806 | >CONTIGS_30423 | (GCTCAT)3 | TATTGGAGGTGTGGAAGCAAG | AATTGGGATTAGGACAGAACCA | 296 |
| VMgSSR807 | >CONTIGS_30628 | (TTAT)4 | CAGAGTAGCTGGGACTACAGGC | GCTTGGGCAACATAGTAAGACC | 110 |
| VMgSSR808 | >CONTIGS_30674 | (CCG)5 | CTCTGCGAAGGAAAAGTCAGTC | GAACCGCCTCCAGTCCTC | 118 |
| VMgSSR809 | >CONTIGS_30793 | (TTC)5 | AAGTTTTACATGGCACCAGCTT | GAACCTTTTACCCTTCGGTGTAG | 219 |
| VMgSSR810 | >CONTIGS_30936 | (TG)6 | TCTCGCTGGAACCACCTTCTATG | CATGCCTTGACCCCACCC | 100 |
| VMgSSR811 | >CONTIGS_31042 | (CCATGT)3 | AGATAGAAGGCGAAGCGTTATG | GAGAGAGGTGAAGGTGGAAAAG | 145 |
| VMgSSR812 | >CONTIGS_31218 | (TC)6 | GTAAATTGTTCCCTCCCCTCTC | TCCCAGGTGATTCCTATTCATT | 183 |
| VMgSSR813 | >CONTIGS_31358 | (TTCT)4 | TTGTCAGTATCCGAGGAAAGGT | CTTGCCATGTTTCTCACCCTAT | 380 |
| VMgSSR814 | >CONTIGS_31551 | (AAT)14 | TGTAGCGTTCAGAGAGCCAATA | CTAGTGTGGTGTGGGACGAAG | 190 |
| VMgSSR815 | >CONTIGS_31561 | (TCT)5 | TATGTTGATTGTTCCCACCAGA | AACGCTAGACACAAGCTCCAGT | 248 |
| VMgSSR816 | >CONTIGS_31684 | (CA)14 | CCTGGCACACAGTAGAAGCTC | TTCCTCGTCTTGTCTCGTCTTT | 144 |
| VMgSSR817 | >CONTIGS_31839 | (CTT)6 | TTTCCTTGTGTGTATTCCCTCC | TCTGATTCTCACCACCGTTCT | 101 |
| VMgSSR818 | >CONTIGS_32079 | (GCCACA)3 | TTCTTCTCAATCCTCCACACCT | GCTTTGGCGATAGTGGTCTTAC | 363 |
| VMgSSR819 | >CONTIGS_32092 | (TCA)6 | ATCAGTACCTTGCCGTTTTCTC | TTTGTGAGAGAGTTGGAGGATG | 261 |
| VMgSSR820 | >CONTIGS_32302 | (TC)12 | CTCTCTCTCTCTAGCTCTCGCC | TGAAGGATCAGAACACAGCTTC | 101 |
| VMgSSR821 | >CONTIGS_32338 | (CAG)5 | GTGCAAACACTCAAAGCAACAC | GGCTGGGACTATTCTGGTTCAT | 135 |
| VMgSSR822 | >CONTIGS_32348 | (AGAAAA)3 | CGGAGTATAGACAGTGGCAGAA | ATAACATCGCATCGCACTACAT | 207 |
| VMgSSR823 | >CONTIGS_32366 | (TA)8 | ATCGAGTGAGTTTTGCTGAAAG | TCTATCTTTTCCCATGTCCTAGC | 253 |
| VMgSSR824 | >CONTIGS_32475 | (CTC)5 | TAAGTCAGGAACGTCGGTATCA | TACTGCAAACCATAAGTGCCC | 100 |
| VMgSSR825 | >CONTIGS_32652 | (TG)8 | CTAGGATGGGACGAACATGG | GCGGTGGGTGTTGTTGTATT | 156 |
| VMgSSR826 | >CONTIGS_32748 | (TA)6 | TGGAGAGGAGCTGTAATTTTGC | TGAGTTGTGAAAGGGGAAATG | 259 |
| VMgSSR827 | >CONTIGS_32753 | (CT)8 | CCCAAACACACAACACAAAAGT | ATGCTTCTGATTATGCCCATCT | 340 |
| VMgSSR828 | >CONTIGS_33051 | (TGG)5 | AAAGGGGACAAGAAAGAACCTC | ATTCCCACCACCTGAAACTG | 165 |
| VMgSSR829 | >CONTIGS_33182 | (TA)7 | TTTACCGTGATACTGAGACTAAAGC | CATGATCCACCACCATTTTC | 130 |
| VMgSSR830 | >CONTIGS_33220 | (TAC)5 | ACTCCACCTTATTGTCCAGCAT | TTTGTGAGTGTGCCTTATAGCG | 301 |
| VMgSSR831 | >CONTIGS_33383 | (AACAT)4 | TGGAGAAAGGGAGAGTATTTCG | CACGTCAACACCGTCCTTC | 225 |
| VMgSSR832 | >CONTIGS_33388 | (GT)19 | GAGAATGCCACTTATGCCTCTT | GGTCCTCTCTGTCTCCCAGAT | 235 |
| VMgSSR833 | >CONTIGS_33432 | (TAAA)4 | CATTAGATGCGGTCCAGGTAA | TGAGATGCACCCTACTACATCG | 193 |
| VMgSSR834 | >CONTIGS_33466 | (CA)13 | AGCCCCTGTATTAGCCAGAGT | CAGGATCAGTCTTGTTTCACCT | 294 |
| VMgSSR835 | >CONTIGS_33476 | (TTCAAT)4 | AGAGACGCATGACACGAAAC | TAAGGTAGGGAAAAGGGGTAGC | 234 |
| VMgSSR836 | >CONTIGS_33522 | (CA)7 | CCATAACCGAAAACAGAACCAT | TGAGGGAGGAATTAGGAATCAA | 215 |
| VMgSSR837 | >CONTIGS_33893 | (CG)6 | GAGCGAGCACGAGATGGT | AAGTTCCCGTCGAAAATCAG | 191 |
| VMgSSR838 | >CONTIGS_33939 | (AG)6 | GGAACAAGTACACCGGAAACTC | GACCTCATTCACCTCCTTCAAC | 116 |
| VMgSSR839 | >CONTIGS_33967 | (CCGAAC)3 | ATCATACACTTGGAGAAGGGAG | GATAGTCTCAACCGTCACAAAAG | 149 |
| VMgSSR840 | >CONTIGS_34017 | (ACC)5 | CCAGGAAGATTAAGGGACACAG | CTGACTACAAAACGTGTGGGAA | 235 |
| VMgSSR841 | >CONTIGS_34090 | (TA)8 | CATAACCGTGTGAGCCAATTCT | GGAGTATCCAGAAGAAGGGAGG | 352 |
| VMgSSR842 | >CONTIGS_34281 | (CAG)10 | GGTTCCCAGAGACAAATTCAAG | GAGCAAGAGGTTGTGCTGTTT | 216 |
| VMgSSR843 | >CONTIGS_34307 | (GGA)6 | CAAGGAGACAGGGAGGAGAGT | ACAACAACCACCACCTCTACG | 125 |
| VMgSSR844 | >CONTIGS_34338 | (ATGATC)3 | GTGTTGTTTCGTGAGGATGG | GTTGGTTGTCTCAATAGGAGGG | 203 |
| VMgSSR845 | >CONTIGS_34444 | (CCTTCT)3 | ATTCGTCTTCGATCTCCTCCTT | AGAAGGGTTTGAGTGGTCAGAG | 164 |
| VMgSSR846 | >CONTIGS_34482 | (ACG)5 | GACTCCTTCCTCATCGGCTC | AGCAGGTTCTTGCCCGTATC | 176 |
| VMgSSR847 | >CONTIGS_34512 | (AG)6 | CAGCAGAGGCAAACCCTTC | CCAAGTCTCTCTCTCTCCGATG | 202 |
| VMgSSR848 | >CONTIGS_34701 | (CAG)5 | GCAATTCAGCAATCCGTATCTT | AAACCAGAGTGTTGCCTTTGAT | 197 |
| VMgSSR849 | >CONTIGS_34848 | (GC)6 | GAAATCACCGGCTTCCCG | CGCAGGATCTCGTGGAATTG | 176 |
| VMgSSR850 | >CONTIGS_35047 | (TG)6 | TCAAAGGTTTAGCAAGAGTGGC | CGTAGCCAACTTTACAACCAGC | 296 |
| VMgSSR851 | >CONTIGS_35188 | (TG)6 | TGAGATGGGTGGAAAACTCTACT | CTCAGCACTGTTTATTTGGTGG | 212 |
| VMgSSR852 | >CONTIGS_35193 | (GAAA)4 | CCCAATCCAAAACCAAAGAGA | TCAGAAACAGGAGTATCGAGGG | 148 |
| VMgSSR853 | >CONTIGS_35318 | (CCCTGC)4 | CATGGACACCAAAAGAGGGTAT | AAAACAAACCATCCTCAACAGG | 137 |
| VMgSSR854 | >CONTIGS_35442 | (GGT)5 | GTGGTGGAGATGGGTAGTGGTA | GGTCCCTAATGGCTTCTTCTG | 195 |
| VMgSSR855 | >CONTIGS_35446 | (TG)6 | AGGTGCTCAGGATTGGGG | AGAGGGAAGTGAGGCCATCT | 170 |
| VMgSSR856 | >CONTIGS_35574 | (TC)13 | GCTTCAGCACTTCAGCAATCT | GAACAAAGGGGAGAGTAAGCAA | 117 |
| VMgSSR857 | >CONTIGS_35884 | (TCG)5 | CTTTCGGTCCTCTTCCTCTTC | CTACTCCCGACCTCAAACAAAC | 230 |
| VMgSSR858 | >CONTIGS_36136 | (GGTCTG)3 | GTGCCGAGCGTGAACAGC | GCGAACAGCTACGGATCAGC | 166 |
| VMgSSR859 | >CONTIGS_36285 | (TAGA)4 | AGGGTTATGCCTACTGAGGTGA | CCCTTCCTCCCAAAATCAAT | 231 |
| VMgSSR860 | >CONTIGS_36289 | (CAAA)5 | GCCATGTATTGTAAGGCACATC | CAACAGACCTTTTAGGAGGGTG | 181 |
| VMgSSR861 | >CONTIGS_36342 | (GA)6 | ATGAGAAGCATAGGTGGGAGAG | TCCATCTTCAAAGCTACCAACA | 146 |
| VMgSSR862 | >CONTIGS_36504 | (AC)6 | ACCATCCATATTCCTCCATCAC | ACCATCTTTCTGTTGAGCCTGT | 176 |
| VMgSSR863 | >CONTIGS_36965 | (GGT)9 | GTGGTGAAGGAGGAAGAAACAG | AGTGCTCCAAGACACAACAGAA | 198 |
| VMgSSR864 | >CONTIGS_37080 | (TCT)7 | TAGCATTGGACTGTGCTGATCT | GGCAACAACAGAGGATACTTGA | 171 |
| VMgSSR865 | >CONTIGS_37122 | (ATC)5 | CCTTCACTTCTTGTGACCAACTC | GCTAGTGTGCCCTTGAGGTATT | 140 |
| VMgSSR866 | >CONTIGS_37227 | (CCAACA)3 | GATACGACACCACCACAAACC | TAACCAGATTCAAGCAATGACG | 179 |
| VMgSSR867 | >CONTIGS_37301 | (TC)9 | TGTCTGAACTTGCTGGTATGCT | CTTACACCATGAGAGTGGCCTT | 239 |
| VMgSSR868 | >CONTIGS_37404 | (CAC)5 | AGTTTCCACAACCCAAGATTCA | GCGCTGAAGGTGATAGAGTTG | 285 |
| VMgSSR869 | >CONTIGS_37568 | (TG)7 | GAAAGGTGCTGTTTGAGATGAC | TAATGGCTTCCTTCCCTACATC | 210 |
| VMgSSR870 | >CONTIGS_37839 | (AAAAC)4 | CGAAGAGCCAGAAAGGAAGTAA | AGTCCGCAAACATAGCGTTTAT | 144 |
| VMgSSR871 | >CONTIGS_37935 | (AG)6 | GGTTTCTTCATTCTTCTCTTCG | TTTTCAGTCAGCACACTCCTTA | 245 |
| VMgSSR872 | >CONTIGS_37971 | (CGG)6 | ACACCCTAGTCTCCGTCGC | TTATTCTCTCTCTCCCTCCACG | 105 |
| VMgSSR873 | >CONTIGS_38063 | (TTC)5 | GTGAGGAAGAGGGAGAAGTTGA | AGTGCTTGGCATTGAAGGAT | 237 |
| VMgSSR874 | >CONTIGS_38066 | (AGA)6 | CAGTTCCAAACCAGTATGCTCA | GTAAGTGCTCCTCCCATTCAAG | 111 |
| VMgSSR875 | >CONTIGS_38139 | (GCC)6 | GGTGCTGCCACTTCCGCT | CTATTCCCTCTGCCTGCCAAGT | 158 |
| VMgSSR876 | >CONTIGS_38220 | (GGC)5 | CGCTGTTCGGTGAGCTTT | CTAGCAGGGTAGCTGCGATAA | 148 |
| VMgSSR877 | >CONTIGS_38443 | (CAC)5 | GAGGCTGTAGTAGCTGAAGGGA | TTCCACGATTTGGTATTGACTG | 277 |
| VMgSSR878 | >CONTIGS_38477 | (TA)7 | GCTGCTGCTCTTCACAAACTC | GTGGACCTTTACAAGCCCTACTT | 346 |
| VMgSSR879 | >CONTIGS_38573 | (AGA)5 | AGTGACAGGGTTGAAGAGAAGC | CCACCTATTTCCAAGGAGACTG | 142 |
| VMgSSR880 | >CONTIGS_38643 | (TCT)5 | GCTTTCATCCTAATGCTTCTTC | CTCTCACCTTGTTGTTTTCTCC | 213 |
| VMgSSR881 | >CONTIGS_38674 | (CT)7 | GGGAATGAGTGCTTTTGCTTTA | ATCTGCTTCTGTGAATGCCAC | 163 |
| VMgSSR882 | >CONTIGS_38722 | (TCAGCC)3 | CCACCCCTTCCCCAAAAG | GCTGACCCTAGATCACAGAGCA | 232 |
| VMgSSR883 | >CONTIGS_38748 | (CAG)8 | AGGATCTTCCACCCAGTATGAG | CTGAAGTTGGGGAAAACAGAAG | 192 |
| VMgSSR884 | >CONTIGS_38992 | (GC)6 | TGGTTTCTATCAATGCGCTCTA | TTCAAACAACGGTGCCAG | 191 |
| VMgSSR885 | >CONTIGS_39017 | (ACGGCG)3 | CTCAAGCATTCCCTTTCTCGT | CAGAGTCCCCGATCAGCA | 104 |
| VMgSSR886 | >CONTIGS_39160 | (TC)10 | CCTTCTCAATATGTCTAACCCCA | GTAGCAGCAGCATGTAAGGATG | 202 |
| VMgSSR887 | >CONTIGS_39162 | (TC)7 | GGTCCATGTGAACAACAGAATAC | CCTCTTTCCAGGGCTGTG | 175 |
| VMgSSR888 | >CONTIGS_39347 | (GA)6 | AGAAAAGGGGAAGCAAGGTACA | CCTGGTGGGAGGATTTTAAGTG | 157 |
| VMgSSR889 | >CONTIGS_39391 | (CTT)5 | CTTGTTGGTGGTGTTGTTGTTT | AACATCGGTGATCTCTTTGGAG | 249 |
| VMgSSR890 | >CONTIGS_39616 | (TAAAAA)3 | CAGCCCAAAATCCATTAAGGT | GGTGCCATGTATCAGGTGTAGA | 182 |
| VMgSSR891 | >CONTIGS_39898 | (TC)6 | TGACAACGACTGTGTAAGCAGA | TAAGTCATCAGGAATGCAGGG | 149 |
| VMgSSR892 | >CONTIGS_39934 | (CT)6 | TCTTTGACTCCGTCTTTGTCTCC | GTTGGCAGAGGCAGGGAA | 117 |
| VMgSSR893 | >CONTIGS_40040 | (AG)7 | AGAGATGTAACGATGGTGGCTT | CCTTTCAACTTTCAACCTTTCC | 303 |
| VMgSSR894 | >CONTIGS_40408 | (CCG)5 | CTGCGTCACTTCGCTTTGATA | CAGCTTATCCTGCCTCTCAGTC | 219 |
| VMgSSR895 | >CONTIGS_40421 | (GA)6 | GGGAAGACAGGGAAAGTGATTA | TCAGCAGAAACTAGGACACAGC | 282 |
| VMgSSR896 | >CONTIGS_40442 | (CTT)7 | CGCAGTACCAGAACGTCATAAA | TCTGCCTGCCAAGGACAT | 368 |
| VMgSSR897 | >CONTIGS_40925 | (TATTAC)3 | ACTAGCAACATCCTCACCCCTA | CGGTTTCTTTGGAAATATGCTC | 143 |
| VMgSSR898 | >CONTIGS_41100 | (AAT)6 | AGTCCATTTGGCTATCCCTTTC | AATTGATCTGGTATTCCTTGCG | 169 |
| VMgSSR899 | >CONTIGS_41266 | (TGC)5 | CCTGGTGCTGAACGAAAGTTA | GGTTTTCTGGGTCTGCTTCTTT | 132 |
| VMgSSR900 | >CONTIGS_41357 | (AG)6 | AGGTAATTGAGCCAGTCAGAGC | TTATTTCGGGCTAACAGAGGTG | 246 |
| VMgSSR901 | >CONTIGS_41360 | (TGG)5 | ATATTGGGCATAGGAAAGAGGC | GAGGAGGGTGAGTTTGTTGTTC | 198 |
| VMgSSR902 | >CONTIGS_42043 | (TTTTTC)5 | CAGCGACAATTCCTGCTCTAA | TGTGCCTTGATGCTTAGTTACG | 163 |
| VMgSSR903 | >CONTIGS_42858 | (CA)5 | CCCTTTCCTGAGATTTCCATT | TCTGTAGCAGCATTCTCGTTGT | 148 |
| VMgSSR904 | >CONTIGS_42988 | (GC)5 | GGTGATCCTTCTGTGCTGG | GTGATGTCGAACTCCTCGG | 194 |
| VMgSSR905 | >CONTIGS_43114 | (TG)7 | TTGTGTGAGACCAAAATAGGTG | TCTTTGCCCCTTGTAGTTGA | 100 |
| VMgSSR906 | >CONTIGS_43117 | (TC)5 | CTCTCCGACTACCTCCAGTCTC | GAAGAATTTGAGGGCGAGC | 102 |
| VMgSSR907 | >CONTIGS_43171 | (AAG)5 | GGCTACAAACATCCCCACATA | TAACATACTTCAGCCACGAAGG | 158 |
| VMgSSR908 | >CONTIGS_43286 | (AG)6 | AGGAGGCTGCAATGTTGGAC | GCCTTTGCTAATTCCTGAACTTGT | 166 |
| VMgSSR909 | >CONTIGS_43308 | (GAA)7 | GCTGGTCTCTGATTTCACCTTC | AGTTACCCACACCACTCTCTCC | 255 |
| VMgSSR910 | >CONTIGS_43469 | (AAAAAG)3 | ATAAGGTGAGGCTGAAGCTGAA | GGCTCTTACATGGTATCAAATCCT | 187 |
| VMgSSR911 | >CONTIGS_43514 | (CTC)5 | TCCTTCTCTCTCTTCCCGTTCT | GGAGTGTTTGGGCTTGTGAT | 129 |
| VMgSSR912 | >CONTIGS_43631 | (GGCTGG)3 | TGATCCCATGTTTGTGTTTCTC | CCCAACCTACCTCCTTGGTAAT | 125 |
| VMgSSR913 | >CONTIGS_43846 | (AATT)4 | TTTGACTGATAAAATGCCCC | GGTTTAGCTCTTGTTTCTCCAA | 113 |
| VMgSSR914 | >CONTIGS_43905 | (TG)6 | CGAATGTGATGCTAAAGAGTAGAGT | CTTTCAACGATTCCTCCTTCT | 191 |
| VMgSSR915 | >CONTIGS_44029 | (CTG)5 | GCTTCGATGACGGTGCTG | CGAGCCGATAGTCAGGATAATG | 279 |
| VMgSSR916 | >CONTIGS_44068 | (AC)7 | GCATCATTTTCCTCTCTGCTG | GCTTGCACGAGTGTGTGTG | 140 |
| VMgSSR917 | >CONTIGS_44105 | (GTTT)5 | GGCTTTGTTGGCAGGTTTT | GCACACCTAATCAAGGGTTCAC | 108 |
| VMgSSR918 | >CONTIGS_44123 | (GT)6 | GGACTGGAGTGGTGAGAAGGTA | TCAGATTGTTGTCTTTGTTGGG | 106 |
| VMgSSR919 | >CONTIGS_44372 | (TTG)5 | GGCGAATGTTATCAGAAGAAGC | AGTGAAACCACACGTACACCG | 106 |
| VMgSSR920 | >CONTIGS_44553 | (TG)6 | GATGGAATCTGAGTGGTGGTTT | CTGTGCATCAACCTCATGTCTT | 134 |
| VMgSSR921 | >CONTIGS_44932 | (TGA)5 | CGAGTGAGACTAGGGACTCATGT | GAGGAATAAGCCGGTCTACATCT | 176 |
| VMgSSR922 | >CONTIGS_45730 | (CA)6 | CCTCTTTCTCTTTCGGTCTCTG | ATGTACCAAGCGTGACAAGG | 153 |
| VMgSSR923 | >CONTIGS_46100 | (AAT)5 | GAATCTGCCATTCACACCTAACT | AAGAACATTCGGCCATAAACTG | 207 |
| VMgSSR924 | >CONTIGS_46349 | (GATGCC)3 | TACGACCGTTACATGGGACA | AGGGAAGAACTTACTGGTCTGC | 229 |
| VMgSSR925 | >CONTIGS_46456 | (AAAGAA)3 | GCTCCTTTCTTGTCTTCATGCT | TCCAGCAAGTTCAGGGAAAAT | 159 |
| VMgSSR926 | >CONTIGS_46644 | (CA)6 | GTGAGCGGATATGCAGTGTG | AGTCAGTTGGTACATGCCTTGA | 218 |
| VMgSSR927 | >CONTIGS_46688 | (CAA)5 | GAAAACAGCTACACTCTTGGGG | AGATACACAGGGTTGGATTGCT | 139 |
| VMgSSR928 | >CONTIGS_46877 | (CCA)5 | TCATGCCCTAGATGTTCGC | AATTGCTGAAAGTGCTGGTACA | 229 |
| VMgSSR929 | >CONTIGS_46885 | (GA)6 | TAACTTTCTATGTTGGCCTGCG | AAGGCATTCACTCAAACACCTC | 120 |
| VMgSSR930 | >CONTIGS_47099 | (TG)8 | CTGTTTATATGCCCCAAAGAGC | CAAATATGAGGAGGGAGAACCA | 203 |
| VMgSSR931 | >CONTIGS_47314 | (CAG)5 | ATCGTCTACAAGGGTCCTCCTC | CACTTGATCTTGCTGAGAATCG | 131 |
| VMgSSR932 | >CONTIGS_47821 | (TA)6 | CATCCTTCATTCCTCTCTCCTC | CGCTACACTTACCCATACAACG | 175 |
| VMgSSR933 | >CONTIGS_48189 | (CT)7 | TTTCTGTTCCAAGGATAAGGGA | AGAGAGAGAGAGAGCACAAGCG | 161 |
