# Supplementary material for: De novo Assembly, Characterization of Immature Seed Transcriptome and Development of Genic-SSR Markers in Black Gram [Vigna mungo (L.) Hepper]
Source: PLoS One. 2015 Jun 4;10(6):e0128748. doi: 10.1371/journal.pone.0128748 (PMC4456365; doi:10.1371/journal.pone.0128748)
Supplement: S3 Table — (DOCX) [file pone.0128748.s006.docx]

**S3 Table. Number of alleles per locus and polymorphic information content (PIC) value of genic-SSR markers used for studying allelic variation among black gram genotypes.**

| **Genic-SSR primer code** | **No. of alleles /locus** | **Polymorphic information content (PIC)** |
| --- | --- | --- |
| VMgSSR-1 | 2 | 0.20 |
| VMgSSR-2 | 2 | 0.41 |
| VMgSSR-3 | 2 | 0.20 |
| VMgSSR-4 | 1 | 0.00 |
| VMsSSR-5 | 3 | 0.28 |
| VMgSSR-6 | 2 | 0.21 |
| VMgSSR-16 | 2 | 0.20 |
| VMgSSR-18 | 2 | 0.20 |
| VMgSSR-19 | 2 | 0.20 |
| VMgSSR-20 | 2 | 0.50 |
| VMgSSR-24 | 3 | 0.45 |
| VMgSSR-29 | 2 | 0.50 |
| VMgSSR-30 | 3 | 0.21 |
| VMgSSR-31 | 3 | 0.28 |
| VMgSSR-53 | 2 | 0.31 |
| VMgSSR-54 | 2 | 0.28 |
| VMgSSR-78 | 2 | 0.20 |
| VMgSSR-79 | 2 | 0.11 |
| VMgSSR-80 | 2 | 0.45 |
| VMgSSR-81 | 2 | 0.20 |
| VMgSSR-82 | 2 | 0.50 |
| VMgSSR-83 | 2 | 0.21 |
| VMgSSR-87 | 2 | 0.28 |
| VMgSSR-94 | 2 | 0.11 |
| VMgSSR-96 | 2 | 0.20 |
| VMgSSR-115 | 3 | 0.28 |
| VMgSSR-116 | 2 | 0.21 |
| VMgSSR-203 | 3 | 0.38 |
| VMgSSR-332 | 2 | 0.20 |
| VMgSSR-460 | 2 | 0.21 |
| VMgSSR-814 | 1 | 0.20 |
| VMgSSR-842 | 2 | 0.28 |
| Average | 2 | 0.26 |
