# Supplementary material for: De novo Assembly, Characterization of Immature Seed Transcriptome and Development of Genic-SSR Markers in Black Gram [Vigna mungo (L.) Hepper]
Source: PLoS One. 2015 Jun 4;10(6):e0128748. doi: 10.1371/journal.pone.0128748 (PMC4456365; doi:10.1371/journal.pone.0128748)
Supplement: S4 Table — (DOCX) [file pone.0128748.s007.docx]

**S4 Table.** **Transferability of black gram genic SSR markers to other *Vigna* species**

| Locus | *V. mungo* | *V. mungo var. silvestris* | *V. radiata* | *V.*  *unguiculata* | *V.*  *angularis* | *V.*  *umbellata* | *V.*  *glabrescens* | *V.*  *aconitifolia* | *V.*  *trilobata* | *V.*  *Vexillata* |
| --- | --- | --- | --- | --- | --- | --- | --- | --- | --- | --- |
| VMgSSR-1 | + | + | + | + | + | - | - | - | - | - |
| VMgSSR-2 | + | + | + | + | + | + | + | + | - | - |
| VMgSSR-3 | + | + | + | + | + | - | - | - | - | - |
| VMgSSR-4 | + | + | + | + | + | + | - | - | - | - |
| VMgSSR-5 | + | + | + | + | + | + | - | + | + | - |
| VMgSSR-6 | + | + | + | - | + | - | - | - | + | + |
| VMgSSR-16 | + | + | - | + | + | + | - | - | - | - |
| VMgSSR-18 | + | + | + | + | - | + | - | + | + | - |
| VMgSSR-19 | + | + | + | + | + | + | + | + | - | - |
| VMgSSR-20 | + | + | + | + | + | - | - | + | + | - |
| VMgSSR-23 | + | + | + | + | + | - | + | - | - | - |
| VMgSSR-24 | + | + | + | + | - | + | + | - | - | + |
| VMgSSR-29 | + | + | + | - | + | + | + | + | + | + |
| VMgSSR-30 | + | + | + | + | + | + | + | - | + | - |
| VMgSSR-31 | + | + | + | + | + | - | + | + | - | - |
| VMgSSR-52 | + | + | + | + | + | + | + | + | - | - |
| VMgSSR-53 | + | + | + | + | - | + | + | - | + | - |
| VMgSSR-54 | + | + | + | - | + | - | - | - | - | - |
| VMgSSR-55 | + | + | - | + | + | + | + | - | + | + |
| VMgSSR-78 | + | + | + | + | + | + | + | + | + | + |
| VMgSSR-79 | + | + | + | + | - | + | + | + | + | + |
| VMgSSR-80 | + | + | - | + | + | + | + | + | - | - |
| VMgSSR-81 | + | - | - | + | - | - | - | - | - | - |
| VMgSSR-82 | + | + | + | + | - | - | - | + | + | + |
| VMgSSR-83 | + | + | + | + | - | - | - | - | - | - |
| VMgSSR-84 | + | + | + | + | - | - | - | + | + | - |
| VMgSSR-85 | + | + | - | + | + | + | + | + | + | - |
| VMgSSR-87 | + | + | + | - | + | + | + | + | + | - |
| VMgSSR-94 | + | + | + | + | + | - | - | + | + | + |
| VMgSSR-95 | + | + | - | + | - | + | + | - | - | - |
| VMgSSR-96 | + | + | + | - | + | + | + | + | - | - |
| VMgSSR-114 | + | + | - | + | - | - | - | - | + | - |
| VMgSSR-115 | + | + | - | - | + | + | + | + | + | - |
| VMgSSR-144 | + | + | + | + | - | - | - | - | + | - |
| VMgSSR-201 | + | + | + | + | + | + | - | - | - | + |
| VMgSSR-293 | + | + | + | + | + | + | + | + | - | - |
| VMgSSR-511 | + | + | - | + | - | + | + | - | + | + |
| VMgSSR-616 | + | + | - | + | + | + | - | - | - | - |

+ = Present; - = absent
